# A New Model Based on Adaptation of the External Loop to Compensate the Hysteresis of Tactile Sensors

**DOI:** 10.3390/s151026170

**Published:** 2015-10-15

**Authors:** José A. Sánchez-Durán, Fernando Vidal-Verdú, Óscar Oballe-Peinado, Julián Castellanos-Ramos, José A. Hidalgo-López

**Affiliations:** 1 Universidad de Málaga, Andalucía Tech, Departamento de Electrónica, ETSI Informática, Campus de Teatinos, 29071 Málaga, Spain; E-Mails: jsd@u ma.es (J.A.S.-D.); oballe@uma.es (Ó.O.-P.), julian@elca.uma.es (J.C.-R.), jahidalgo@uma.es (J.A.H.-L.); 2 Instituto de Investigación Biomédica de Málaga (IBIMA), 29010 Málaga, Spain

**Keywords:** tactile sensors, asymmetrical hysteresis nonlinearity compensation

## Abstract

This paper presents a novel method to compensate for hysteresis nonlinearities observed in the response of a tactile sensor. The External Loop Adaptation Method (ELAM) performs a piecewise linear mapping of the experimentally measured external curves of the hysteresis loop to obtain all possible internal cycles. The optimal division of the input interval where the curve is approximated is provided by the error minimization algorithm. This process is carried out off line and provides parameters to compute the split point in real time. A different linear transformation is then performed at the left and right of this point and a more precise fitting is achieved. The models obtained with the ELAM method are compared with those obtained from three other approaches. The results show that the ELAM method achieves a more accurate fitting. Moreover, the involved mathematical operations are simpler and therefore easier to implement in devices such as Field Programmable Gate Array (FPGAs) for real time applications. Furthermore, the method needs to identify fewer parameters and requires no previous selection process of operators or functions. Finally, the method can be applied to other sensors or actuators with complex hysteresis loop shapes.

## 1. Introduction

Tactile sensors are arrays of force sensing units or taxels. They are used in robotics to detect contact with objects [1,2,3], for instance in handling applications to improve the dexterity of artificial robotic hands [4,5]. Many different approaches have been proposed to make these sensors. Most of them are based on piezoresistive [6,7,8] or capacitive principles [9,10,11], although other transduction principles such as optical [12] or piezoelectric [13] have also been exploited. However, all these sensors show common errors in its operation such as hysteresis, nonlinearity and mismatching [14,15], so it is necessary to compensate these errors to obtain a more precise response. In addition, this compensation has to be carried out in highly demanding real time tasks, so smart tactile sensors with local electronics powerful enough to process the large amount of data from the sensor array in real time are required. The authors have proposed smart tactile sensors with local electronics based on FPGAs [16,17]. These devices consist of logic blocks and dedicated modules that allow the implementation of complex arithmetical operations, and their main advantage is the parallel execution of processes [18]. Therefore, this approach seems suitable to implement the compensation algorithms in real time control tasks. However, the complexity of the involved mathematical operations affect the speed and consumption of power and system resources, so another significant goal is to reduce both as much as possible.

Virtually all sensors and actuators based on smart materials present undesired complex hysteretic nonlinearities when driven with sufficiently high amplitudes. To compensate for these nonlinearities, as in piezoelectric actuators, many efforts have been made including the feed-forward control as the most common approach [19,20]. The main idea of this compensation is to develop a mathematical model of the hysteresis that can be inverted; the inverted model can then be connected in cascade before the actuator input in order to obtain a linearized response. Likewise, in the case of using a tactile sensor, the inverted model can be placed after the sensor output to compensate the hysteretic behavior and obtain a linearized output. In the case of working with a sensor array, it is also possible to reduce the mismatching between different taxels, since all their outputs are equilibrated at the same time as the hysteresis is compensated. Therefore, the goal is to obtain an accurate model of hysteresis of the sensor and compensate for all these errors.

The hysteresis modeling methods commonly used are phenomenological. Other physics-based models require complex differential equations which need high computing power and a long resolution time, so they are more difficult to implement in the local electronics of smart sensors [21]. However, the phenomenological methods are built from experimental data without considering the physical properties of the actuators or sensors [22]. The most common method to compensate for hysteresis in actuators and sensors is the Preisach model [23,24]. This model is difficult to implement due to the large amount of data required to achieve a good approximation. The Prandtl-Ishlinskii model (PI) [25,26] is a subclass of the Preisach model that has become popular because, unlike the Preisach, its inverse can be calculated analytically and its implementation is much simpler. The classical model of Prandtl-Ishlinskii (CPI) approaches the sensor response with a weighted sum of hysteresis play operators [27] but it can only be used to model symmetrical hysteresis curves. In order to model asymmetrical hysteresis loops, different alternatives have been proposed such as the generalized model of Prandtl-Ishlinskii (GPI) [28], which is based on the use of two different envelope functions to combine with play operators. As a special case of this GPI model, a Prandtl-Ishlinskii model that combines two different asymmetrical play operators is proposed in [29] to independently characterize the ascending and descending branches of a piezoelectric actuator hysteresis loop. Another alternative for the modeling and compensation of the asymmetrical hysteresis nonlinearities is a modified Prandtl-Ishlinskii model (MPI) described in [30], which replaces the linear input function of the classical play operator by a generalized input function based on a third-order polynomial. We can also find hysteresis compensation methods based on the use of two dominant continuous functions, one ascending and another descending, which converge to a turning point without memory saturation [31,32]. The dominant functions here are built from high order polynomials, and the whole model is built from these functions through a nonlinear transformation of the coordinate axis (herein we call it POLY model).

This paper presents a new method to model and compensate hysteresis nonlinearities based on the adaptation of the external loop (herein we call it ELAM). It is a phenomenological model, which builds two continuous and monotonic curves, one increasing and the other decreasing, from linear interpolation of the experimentally measured hysteresis external loop. From these two curves, all internal hysteresis loops are approximated using a different procedure depending on whether it is in an increasing or decreasing branch. Moreover, the approximation is made in two intervals defined by a so called split point, with different adaptations of the external loop in each interval. In order to evaluate the effectiveness of the method, the accuracy of the proposed model is compared with that achieved by other methods referred to above. The methods used for this purpose are the generalized model of Prandtl-Ishlinskii (GPI), the modified model of Prandtl-Ishlinskii (MPI) and that based on polynomial fitting [28,30,31].

A discussion about the number of parameters to be estimated in each model, the computational complexity, and the achieved accuracy is undertaken. The ELAM model is shown as the most accurate method, so its inverse is calculated and the hysteresis nonlinearities of the complete tactile sensor array are compensated. The results confirm that it is a very efficient method to be implemented in real-time control systems using smart tactile sensors. The ELAM method is also applicable to all types of hysteresis loops obtained from other sensors or actuators, and it can provide more accurate models than other methods when the hysteresis loops show complex shapes.

The remainder of this paper is organized as follows. Section 2 shows the tactile sensor and the set-up used to obtain the experimental data. Section 3 explains the different modeling hysteresis methods used to compare with that proposed. Section 4 introduces the so called ELAM method. Section 5 deals with the parameter identification of the models. Section 6 shows the results and related discussions. Finally, some concluding remarks are provided in Section 7.

## 2. Experimental Section

### 2.1. PCB Based Sensor

**Figure 1:**
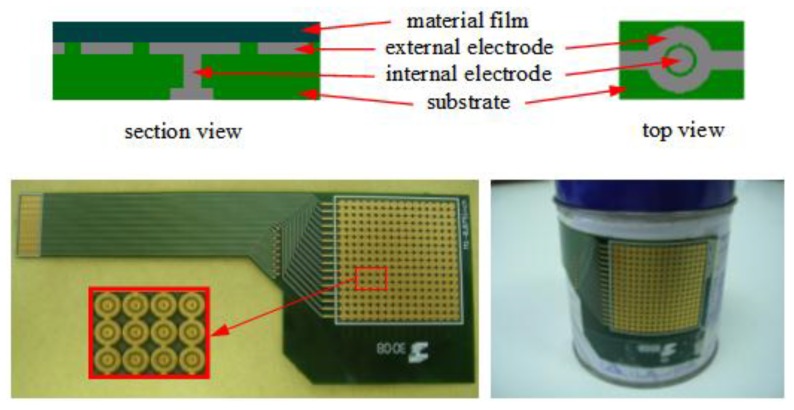
Tactile sensor based on a flexible printed circuit board.

The tactile sensor used in this article consists of a set of electrodes and addressing tracks fabricated on a flexible printed circuit board (PCB). Atop of these electrodes, a thin film of conductive polymer, such as piezoresistive material is placed. Specifically, a conductive water-based ink of this polymer is deposited by spin-coating technique on a flexible plastic sheet, obtaining a smooth, homogeneous and conductive thin film [33,34]. The most interesting thing about this process is that it is cheap and allows the manufacturing of flexible and low cost tactile sensors. The sensor consists of 16 × 16 taxels and a spatial resolution of 2.54 mm. Figure 1 shows a section view and a top view of a taxel, besides a picture of the complete array of the tactile sensor. The resistance between two electrodes associated to each taxel changes when the exerted force against the taxel changes. The readings of the whole tactile array are registered by means of well-known interface electronics [33] designed to achieve a good static performance, so electro-mechanic relays are used to implement the switches to select the rows as the array is scanned. The data acquisition is achieved with the NI-USB 6259 device from National Instruments (National Instruments Corporation, Austin, TX, USA). Sixteen analog inputs are multiplexed to scan up to 16 × 16 taxels in our testing platform.

**Figure 2:**
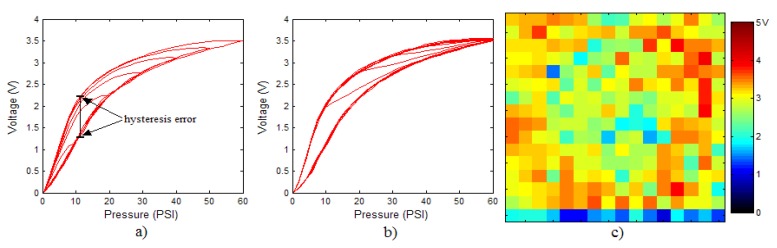
(**a**) Descending behavior of hysteresis curves; (**b**) Ascending behavior of hysteresis curves; (**c**) Mismatching presented by the tactile sensor at a pressure of 40PSI.

**Figure 3:**
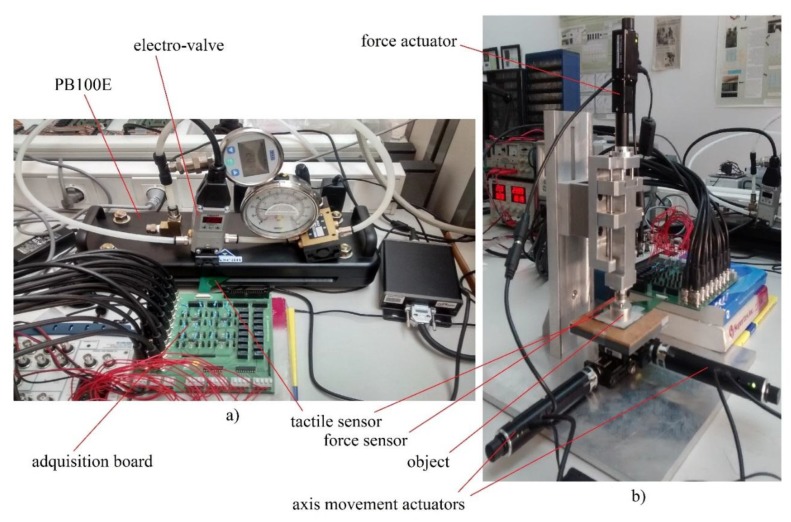
(**a**) Pneumatic commercial setup Tekscan PB100E; (**b**) Motorized stage.

In order to measure the hysteresis of the tactile sensor based on a PCB, a sequence of uniform pressures on the entire matrix of the tactile sensor is exerted. Six consecutive loading-unloading cycles with different points of return for the same ascending curve (see Figure 2a), and with different starting points to rise from the same descending curve (see Figure 2b), are applied. Thus, the descending and ascending behavior of the sensor are respectively characterized. The cycles are performed with an increase of 2 psi between pressures, and in Table 1 the pressure sequences are shown. These hysteresis curves represent the average of the output produced by all of the tactile sensor taxels after repeating each cycle five times. The time interval between the new pressure level being exerted and voltage output being registered by the acquisition board is 2 s. The platform of pneumatic calibration Tekscan PB100E (Teckscan Inc., South Boston, MA, USA) (Figure 3a) is used to apply the uniform pressures over the tactile sensor matrix. In order to quantify the hysteresis exhibited by the sensor, the hysteresis error as the difference in sensor output voltage to the same applied pressure is identified when these pressures are exerted on the ascending and descending branches of the cycles. The maximum and average hysteresis errors are referenced to the highest output value to obtain a percentage of the error relative to full scale. The maximum error due to hysteresis is 25.3% of full scale output, while the average error is 10.0% of full scale out. In Figure 2c, the frame obtained at a pressure of 40 PSI is shown as an example to illustrate the mismatching of the output obtained with the taxels of the tactile sensor.

**Table 1:** Pressure sequences to measure hysteresis loops of the tactile sensor based on a printed circuit board (PCB).

**Pressure Sequence to Measure Descending Curves (see Figure 2a):**																					
PSI:	0➔	60➔	0➔	50➔	0➔	40➔	0➔	30➔	0➔	20➔	0➔	10➔	0								
**Pressure Sequence to Measure Ascending Curves (see Figure 2b):**																					
PSI:	0➔	60➔	10➔	60➔	0➔	60➔	20➔	60➔	0➔	60➔	30➔	60➔	0➔	60➔	40➔	60➔	0➔	60➔	50➔	60➔	0

### 2.2. Measurement Setup

In order to analyze the behavior of the tactile sensor output two different measurement platforms are used. The first is based on a pneumatic commercial equilibration/calibration device (Tekscan PB100E Teckscan Inc., South Boston, MA, USA [35]) (see Figure 3a) to obtain readings of the whole tactile matrix under the same uniform pressure. The sensor is placed in a slot of a chamber where one side is rigid and the other is a flexible wall. When the chamber is pressurized the wall exerts an even pressure on the sensor. An electro-valve Pneumax 171E2N.T.D.0009 (Pneumax S.p.a., Milano, Italy) [36], which allows the flow from a compressor, is added to set the desired pressure selected in a computer software. 

The second platform is used to register the sensor response to pressure exerted by an object with a known shape on the sensor surface. A piece of fabric between the object and the sensor was added to improve the contact. With the purpose of applying a force on the object, a motorized platform is used, which is composed of a T-NA08A50 linear actuator ( Zaber, Vancouver, BC, Canada) and two T-LA60A actuators of Zaber Technologies (Zaber, Vancouver, BC, Canada) [37] (see Figure 3b). The T-NA08A50 actuator (Zaber, Vancouver, BC, Canada) allows a force to be exerted on the *z*-axis, while the T-LA60A actuators allow movement of the platform along the *x* and *y* axes. One sensor Nano17 from ATI Industrial Automation (ATI Industrial Automation, Apex, NC, USA) [38] was added at the tip of the motor in the vertical direction to register the actual force exerted on the objects and then on the sensor.

## 3. Hysteresis Models

**Figure 4:**
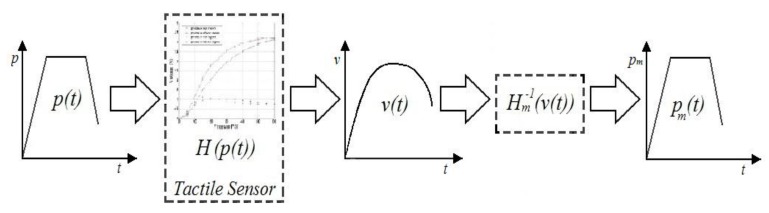
Hysteresis compensation scheme for a tactile sensor.

Figure 4 illustrates the procedure to compensate the hysteresis followed by the four methods that are compared in this paper. The pressure exerted against a taxel is identified by *p*(*t*), and *v*(*t*) is its output voltage. *H*(*p*(*t*)) represents the output with the corresponding measured hysteresis at the sensor. The goal is to find a model *H_m_*(*p*(*t*)) which fits the experimental data *H*(*p*(*t*)) with the greatest possible accuracy, low computational cost, and which can be inverted to obtain *H_m_*^−1^(*v*(*t*)). Once this model is inverted, it is possible to obtain *p_m_*(*t*) which ideally is equal to *p*(*t*). Each of the four methods to compare uses a different mathematical expression of *H_m_*(*p*(*t*)) and employs a number of parameters which must be identified by an error minimization method (see Section 5).

The parameter identification process will identify the set of parameters that configure each of the hysteresis models *H_m_*(*p*(*t*)) which are compared in this study. Due to the fact that they are phenomenological models, a set of M samples of measured output values of the tactile sensor {*y*(*t*_1_),…, *y*(*t_j_*),…*y*(*t_M_*)} are used to computationally derive the vector of parameters X minimizing the following error function [39].

(1) $$J\left(X\right)=\sum_{j=1}^{M}{\left(y_{m}\left(t_{j}\right)-y\left(t_{j}\right)\right)}^{2}$$

where *y_m_* are the samples of the output of each hysteresis model *H_m_*(*p*(*t*)) with 1≤j≤M. In the next sections the models for *H_m_*(*p*(*t*)) are called *H_GPI_*(*p*(*t*)), *H_MPI_*(*p*(*t*)), *H_POLY_*(*p*(*t*)) and *H_ELAM_*(*p*(*t*)), and their output values are named $y_{GPI}\left(t\right)$, $y_{MPI}\left(t\right)$, $y_{POLY}\left(t\right)$ and $y_{ELAM}\left(t\right)$ respectively.

### 3.1. Generalized Prandtl-Islinskii Model (GPI)

**Figure 5:**
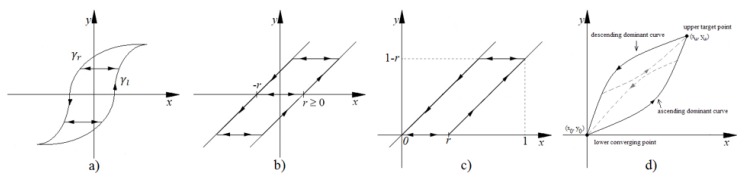
(**a**) Generalized play operator; (**b**) Classical play operator; (**c**) OSP operator; (**d**) Dominant curves.

The generalized model of Prandtl-Ishlinskii (GPI) [28] provides a model for *H*(*p*(*t*)) in the scheme of Figure 4, which we call *H_GPI_*(*p*(*t*)). This model is derived from a weighted superposition of a set of generalized play operators with different threshold values *r* with $r\in R_{0}^{+}$. Since the hysteresis curve of the sensor (see Figure 2a) is asymmetrical, it is necessary to use these generalized operators (see Figure 5a) instead of classical play operators (see Figure 5b). Note that an increase of input corresponds to an increase of output along the curve $\gamma_{l}$, while a decrease of input corresponds to a decrease of output along the curve $\gamma_{r}$. Both curves can be different, so that asymmetrical loops have to be modeled. The only conditions are that these so called envelope functions, $\gamma_{l}$ and $\gamma_{r}$, be monotone and continuous functions with $\gamma_{l}-r\leq \gamma_{r}+r$.

If a finite number *n* of operators is used [28], the output of the generalized Prandtl-Ishlinskii model can be approximated, by the expression.

(2) $$H_{GPI}(p(t))=y_{GPI}\left(t\right)=\sum_{i=0}^{n}p\left(r_{i}\right)\cdot H_{r_{i}}\left[x\right]\left(t\right)$$

where *p*(*r*) is the density function and H is the generalized play operator in Figure 5a defined from the envelope functions $\gamma_{l}$ and $\gamma_{r}$ as

(3) $$H_{r_{i}}\left[x\right]\left(t\right)=H\left(x,y,r_{i}\right)=\max\left\{\gamma_{l}\left(x\right)-r_{i},\min\left\{\gamma_{r}\left(x\right)+r_{i},y\right\}\right\}$$

with *y* as the output value of the generalized play operator for the previous value of *x*.

Therefore, the generalized Prandtl-Ishlinskii model will be obtained from the set of parameters *X* that define the envelope functions $\gamma_{l}$ and $\gamma_{r}$, the density function *p*(*r*) and the threshold values of the play operators r. The GPI model output $y_{GPI}\left(t\right)$ is used in Equation (1) to obtain the parameters *X* (see Section 5).

### 3.2. Modified Prandtl-Ishlinskii Model (MPI)

The modified Prandtl-Ishlinskii model (MPI) uses the classical play operators for modeling asymmetrical hysteresis such as the one presented by our tactile sensor (see Figure 2a). Although the classical Prandtl-Ishlinskii model (CPI) can only model symmetrical hysteresis curves, this MPI model proposes replacing the linear input function of the CPI model by a generalized input function. Thus, the asymmetric hysteresis nonlinearities can be determined, not only by the weighted superposition of a set of classical play operators but also by the generalized input function [30]. The main advantages of this model relative to the GPI are, as it continues using the classical play operators, that the mathematical description is simpler, the number of parameters to be identified is smaller and its inverse can be calculated analytically from the inverse of CPI model.

The classical Prandtl-Ishlinskii model (CPI) [27] is based on the combination of classical play operators (see Figure 5b) with different thresholds *r*. The modification of the play operator by a one-side operator (OSP) is proposed in [30] when the sensor or actuator only works with positive excitation signals. Then, the play operator (OSP) (see Figure 5c) is expressed as

(4) $$H_{OSPr_{i}}\left[x\right]\left(t\right)=H_{OSP}\left(x,y,r_{i}\right)=\max\left\{x-r_{i},\min\left\{x,y\right\}\right\}$$

Using a finite number *n* of OSP operators the output of the MPI model is

(5) $$H_{MPI}(p(t))=y_{MPI}\left(t\right)=g(x(t))+\sum_{i=0}^{n}b\left(r_{i}\right)\cdot H_{OSPr_{i}}\left[x\right]\left(t\right)$$

where $b\left(r_{i}\right)=p(r_{i})(r_{i}-r_{i-1})$, *p*(*r*) is a density function and the function *g*(*x*(*t*)) replaces the linear input function $p_{0}\cdot x(t)$ in the CPI model to achieve the modeling of asymmetric hysteresis [30].

### 3.3. Polynomial Based Model (POLY)

Methods have been proposed to compensate the hysteresis using mathematical structures that are not built with play operators. This is the case of the model described in [31], which is based on the construction of the model of hysteresis of a piezoelectric actuator from the external curves of the hysteresis data. This external loop consists of an ascending dominant curve when the input values increase, and a descending dominant curve when the input values decrease. All ascending curves converge at one point called upper target point, while all descending curves converge on the same lower converging point (see Figure 5d). The rest of the hysteresis curves can adopt their shape from these dominant curves, which can be expressed as two monotonically continuous functions, *f_ra_*(*x*) and *f_rd_*(*x*), respectively. Third-order polynomials to implement these dominant functions are proposed in [31], so in this paper, we call this model POLY.

The ascending curve function for any ascending trajectory starting from point $\left(x_{1},y_{1}\right)$ and ending at point $\left(x_{2},y_{2}\right)$, is

(6) $$y_{POLY\_a}(x)=y_{1}+\frac{y_{u}-y_{1}}{f_{ra}(x_{u})-f_{ra}(x_{0})}\cdot (f_{ra}(m\cdot x+(1-m)\cdot x_{u}))-f_{ra}(x_{0}))$$

where $m=(x_{u}-x_{0})/(x_{u}-x_{1})$, $\left(x_{0},y_{0}\right)$ is the lower converging point and $\left(x_{u},y_{u}\right)$ is the upper converging point of the dominant curves (see Figure 5d).

Similarly, the descending curve for any trajectory starting point $\left(x_{1},y_{1}\right)$ and ending at point $\left(x_{2},y_{2}\right)$, is

(7) $$y_{POLY\_d}(x)=y_{1}+\frac{y_{0}-y_{1}}{f_{rd}(x_{0})-f_{rd}(x_{u})}\cdot (f_{rd}(n\cdot x+(1-n)\cdot x_{0}))-f_{rd}(x_{u}))$$

where $n=(x_{0}-x_{u})/(x_{0}-x_{1})$.

The output of the model can be expressed as

(8) $$H_{POLY}(p(t))=y_{POLY}(x(t))=\left\{ \begin{array}{l} y_{POLY\_a}(x(t))\;if\;x(t_{i})\geq x(t_{i-1})\\ y_{POLY\_d}(x(t))\;if\;x(t_{i})<x(t_{i-1}) \end{array}\right.$$

## 4. External Loop Adaptation Model (ELAM)

The analysis of the GPI, MPI and POLY methods exposed in the latter sections, shows the presence of play operators, exponential functions, logarithmic, hyperbolic tangent, exponentiation and high degree polynomials, which anticipate a complicated implementation in devices such as FPGAs. Therefore, development of a method based on simple mathematical operations is essential to achieve fast and efficient real time tactile sensors. Furthermore, if one considers the possibility of working with arrays with a large number of taxels (for example, 16 × 16 = 256), each of which must be compensated in real time with its own model of hysteresis, depending on the application, compensation should be performed as fast as possible or with minimal resource consumption and high accuracy.

### 4.1. Direct External Loop Adaptation Model

**Figure 6:**
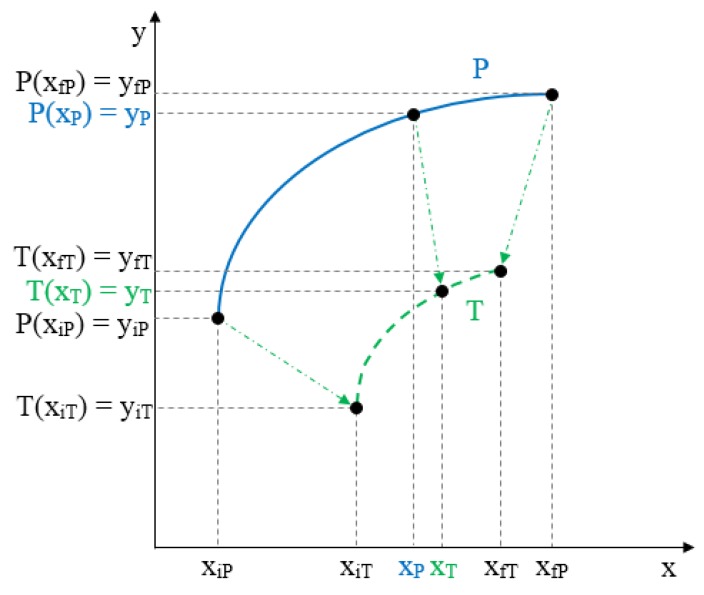
General linear mapping of a pattern curve *P* into a target curve *T*.

The newly proposed approach is based on an adaptation of the external loop of the hysteresis curves to build all the inner hysteretic cycles. Linear interpolation of experimental data from this external loop (see Figure 2) is done to obtain the so called pattern curves, one for the ascending external trajectory and another for the descending external trajectory, so we call them $P_{a}$ and $P_{d}$ respectively. The adaptation is made by piecewise linear mapping of the pattern curves into the input interval of the internal target curves. Figure 6 illustrates the linear mapping of a generic pattern curve *P* between $\left(x_{iP},y_{iP}\right)$ and $\left(x_{fP},y_{fP}\right)$ into the target interval defined by the points $\left(x_{iT},y_{iT}\right)$ and $\left(x_{fT},y_{fT}\right)$ to obtain the target curve *T*.

For each input value $x_{T}$ in $\left[x_{iT},x_{fT}\right]$, the corresponding value $x_{P}$ in $\left[x_{iP},x_{fP}\right]$ obtained by linear mapping is:

(9) $$x_{P}=X_{P}(x_{T},x_{iT},x_{fT},x_{iP},x_{fP})=x_{iP}+\frac{\left(x_{fP}-x_{iP})\cdot (x_{T}-x_{iT}\right)}{\left(x_{fT}-x_{iT}\right)}$$

Now $y_{P}=P(x_{P})$ in $\left[y_{iP},y_{fP}\right]$ is mapped into $y_{T}$ in $\left[y_{iT},y_{fT}\right]$ as

(10) $$y_{T}=T(x_{T})=Y_{T}(y_{P},y_{iT},y_{fT},y_{iP},y_{fP})=y_{iT}+\frac{\left(y_{P}-y_{iP})\cdot (y_{fT}-y_{iT}\right)}{\left(y_{fP}-y_{iP}\right)}$$

A key aspect of the ELAM model is that the target curve is split into pieces and the mapping in Equations (9) and (10) is done in each piece. It has been split into two segments in this work that are defined by the split point $\left(x_{s},y_{s}\right)$ with $x_{iT}<x_{s}<x_{fT}$ and $y_{iT}<y_{s}<y_{fT}$. As a first simple approach, we propose the following linear expressions to find the split point:

(11) $$x_{s}=\alpha \cdot (x_{fT}-x_{iT})+x_{iT}$$

(12) $$y_{s}=\beta \cdot (y_{fT}-y_{iT})+y_{iT}$$

where 0≤α≤1 and 0≤β≤1. The location of this point is determined by an error minimization algorithm as explained below, and the parameters α and β are the same for all target curves. This strategy allows different mappings to be performed at the left and right of the split point to achieve a more accurate fitting of the curve.

**Figure 7:**
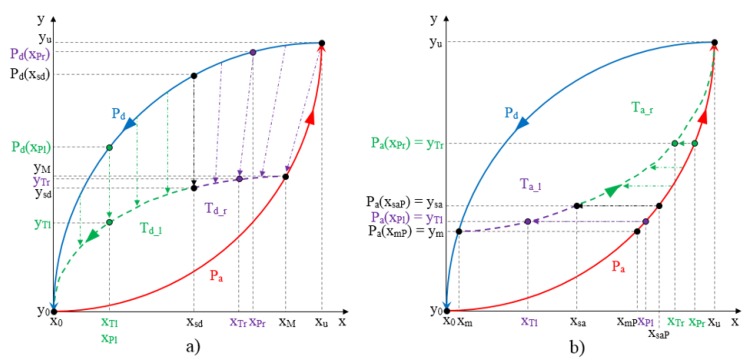
(**a**) Construction of descending curves for ELAM model; (**b**) Construction of ascending curves for ELAM model.

Figure 7a,b illustrate the construction of the descending and ascending trajectories respectively of the hysteresis loops with the ELAM method. As shown in Figure 2a, all descending hysteresis curves converge at the same point ($\left(x_{0},y_{0}\right)$ in Figure 7). Similarly, all ascending curves in Figure 2b converge at the same target point $\left(x_{u},y_{u}\right)$ in Figure 7). The target subcycles are formed by one descending curve, $T_{d}$ in Figure 7a, and another ascending curve, $T_{a}$ in Figure 7b. For the construction of internal hysteresis loops, it is necessary to know the starting points of $T_{a}$ and $T_{d}$, which we call $\left(x_{m},y_{m}\right)$ and $\left(x_{M},y_{M}\right)$, respectively.

Moreover, the split point divides each trajectory into two segments. Specifically, the descending curve $T_{d}$ is composed of $T_{d\_r}$ and $T_{d\_l}$ (see Figure 7a) as

(13) $$T_{d}\left(x\right)=\left\{ \begin{array}{l} T_{d\_r}\left(x\right)\;x_{sd}\leq x<x_{M}\\ T_{d\_l}\left(x\right)\;x_{0}\leq x<x_{sd} \end{array}\right.$$

The coordinates of the split point $\left(x_{sd},y_{sd}\right)$ can be expressed from Equations (11) and (12) as

(14) $$x_{sd}=\alpha_{d}\cdot x_{M}$$

(15) $$y_{sd}=\beta_{d}\cdot y_{M}$$

with $x_{sd}\in [x_{0},x_{M}]$

In order to obtain $T_{d\_r}(x)$ and $T_{d\_l}(x)$ in Equation (13), the linear mapping in Figure 6 is carried out by replacing the initial and final values of the pattern curve *P* and of the target curve *T* in the Equations (9) and (10) (see Figure 6), by the initial and final values corresponding to the pattern curve and the target curve used in the construction of each of the segments. To do this, another split point has to be established in the pattern curve. From inspection of the experimental curves, it is set to $\left(x_{sd},P_{d}(x_{sd})\right)$ for the descending curve, *i.e.*, the split points of both the pattern and target curves share the *x* coordinate.

Therefore, since the descending curve begins at the returning point $\left(x_{M},y_{M}\right)$, and converge to the point $\left(x_{0},y_{0}\right)$ in Figure 7a, if $x_{sd}\leq x_{Tr}<x_{M}$, $P_{d}$ is mapped to the trajectory $T_{d\_r}$ and the output value is obtained from Equations (9) and (10) respectively through the expressions.

(16) $$x_{Pr}=X_{P}(x_{Tr},x_{sd},x_{M},x_{sd},x_{u})=x_{sd}+\frac{\left(x_{u}-x_{sd})\cdot (x_{Tr}-x_{sd}\right)}{\left(x_{M}-x_{sd}\right)}$$

and

(17) $$y_{Tr}=T_{d\_r}\left(x_{Tr}\right)=Y_{T}\left(P_{d}(x_{Pr}),y_{sd},y_{M},P_{d}(x_{sd}),y_{u}\right)=y_{sd}+\frac{\left(y_{M}-y_{sd})\cdot (P_{d}(x_{Pr})-P_{d}(x_{sd})\right)}{\left(y_{u}-P_{d}(x_{sd})\right)}$$

On the other hand, if $x_{0}\leq x_{Tl}<x_{sd}$ the curve $P_{d}$ is mapped to the trajectory $T_{d\_l}$ from the Equations (9) and (10) to obtain (see Figure 7a)

(18) $$x_{Pl}=X_{P}(x_{Tl},x_{0},x_{sd},x_{0},x_{sd})=x_{Tl}$$

and

(19) $$y_{Tl}=T_{d\_l}\left(x_{Tl}\right)=Y_{T}\left(P_{d}(x_{Pl}),y_{0},y_{sd},y_{0},P_{d}(x_{sd})\right)=y_{0}+\frac{\left(P_{d}(x_{Pl})-y_{0})\cdot (y_{sd}-y_{0}\right)}{\left(P_{d}(x_{sd})-y_{0}\right)}$$

Note that the operation to the left of the split point is simply the scaling of the pattern curve.

The output values in Equations (17) and (19) depend on the coordinates of the split point $\left(x_{sd},y_{sd}\right)$ , which depend on $\alpha_{d}$ and $\beta_{d}$ in Equations (14) and (15). An error minimization algorithm can fit the experimental data of the internal cycles in the values provided by Equations (17) and (19) using $\alpha_{d}$ and $\beta_{d}$ as parameters to estimate. This is done as explained in Section 5, and once $\alpha_{d}$ and $\beta_{d}$ are estimated, the coordinates of the split point are readily obtained from Equations (14) and (15) for any other possible descending curve in an internal cycle and the whole descending trajectory is given by Equations (17) and (19).

Similarly, the ascending curve $T_{a}$ begins at the point of return $\left(x_{m},y_{m}\right)$ and converge to the point $\left(x_{u},y_{u}\right)$ (see Figure 7b), and it is composed by $T_{a\_r}$ and $T_{a\_l}$ as

(20) $$T_{a}\left(x\right)=\left\{ \begin{array}{l} T_{a\_r}\left(x\right)\;x_{sa}<x\leq x_{u}\\ T_{a\_l}\left(x\right)\;x_{m}<x\leq x_{sa} \end{array}\right.$$

In this case, the adaptation of the curve $P_{a}$ from the external loop to the desired trajectory $T_{a}$ is performed. The split point $\left(x_{sa},y_{sa}\right)$ is calculated as

(21) $$x_{sa}=\alpha_{a1}\cdot {\left(x_{u}-x_{m}\right)}^{2}+\alpha_{a0}\cdot (x_{u}-x_{m})+x_{m}$$

(22) $$y_{sa}=\beta_{a}\cdot (y_{u}-y_{m})+y_{m}$$

with $x_{sa}\in [x_{m},x_{u}]$. Note that the parameter $\alpha_{a1}$ introduces here a quadratic dependence of the split point location on the length of the interval $\left[x_{m},x_{u}\right]$ that improves the fitting of the target ascending curves. In this trajectory, the linear dependence given in Equation (11) does not provide an accurate enough model, so the next level of complexity to try is the quadratic dependence. Moreover, the split points of the pattern curve $P_{a}$ and the target curve $T_{a}$ are $\left(x_{saP},y_{sa}\right)$ and $\left(x_{sa},y_{sa}\right)$ respectively, where $x_{saP}$ is obtained from $P_{a}(x_{saP})=y_{sa}$, as Figure 7b illustrates. Therefore, both split points do not now share the same *x* coordinate as in Figure 7a, but the same *y* coordinate.

For $x_{sa}<x_{Tr}\leq x_{u}$, the pattern curve $P_{a}$ is mapped into the target curve $T_{a\_r}$ (see Figure 7b) from the Equations (9) and (10) respectively, so we obtain

(23) $$x_{Pr}=X_{P}(x_{Tr},x_{sa},x_{u},x_{saP},x_{u})=x_{saP}+\frac{\left(x_{u}-x_{saP})\cdot (x_{Tr}-x_{sa}\right)}{\left(x_{u}-x_{sa}\right)}$$

and

(24) $$y_{Tr}=T_{a\_r}\left(x_{Tr}\right)=Y_{T}\left(P_{a}(x_{Pr}),y_{sa},y_{u},P_{a}(x_{saP}),y_{u}\right)=P_{a}(x_{Pr})$$

On the other hand, if $x_{m}<x_{Tl}\leq x_{sa}$, the pattern curve $P_{a}$ is mapped into the target curve $T_{a\_l}$ (see Figure 7b) from the Equations (9) and (10) respectively, so we obtain

(25) $$x_{Pl}=X_{P}(x_{Tl},x_{m},x_{sa},x_{mP},x_{saP})=x_{mP}+\frac{\left(x_{saP}-x_{mP})\cdot (x_{Tl}-x_{m}\right)}{\left(x_{sa}-x_{m}\right)}$$

and

(26) $$y_{Tl}=T_{a\_l}\left(x_{Tl}\right)=Y_{T}\left(P_{a}(x_{Pl}),y_{m},y_{sa},P_{a}(x_{mP}),P_{a}(x_{saP})\right)=P_{a}(x_{Pl})$$

where $x_{mP}$ is obtained from $P_{a}(x_{mP})=y_{m}$.

Taking into account that $x_{saP}=P^{-1}(y_{sa})$, the output values in Equations (24) and (26) depend on the coordinates of the split point $\left(x_{sa},y_{sa}\right)$, which depend on $\alpha_{a1}$, $\alpha_{a0}$ and $\beta_{a}$ in Equations (21) and (22). Again, the error minimization algorithm can fit the experimental data of the internal cycles in the values provided by Equations (24) and (26) using $\alpha_{a1}$, $\alpha_{a0}$ and $\beta_{a}$ as parameters to estimate. Once $\alpha_{a1}$, $\alpha_{a0}$ and $\beta_{a}$ are estimated, the coordinates of the split point are readily obtained from Equations (21) and (22) for any other possible ascending curve in an internal cycle and the whole ascending trajectory is given by Equations (24) and (26).

Summarizing, once the set of parameters $X=\left\{\alpha_{d},\beta_{d},\alpha_{a1},\alpha_{a0},\beta_{a}\right\}$ is obtained from the error minimization algorithm, the output of the complete ELAM model is given by

(27) $$y_{ELAM}(x(t))=T(x)=\left\{ \begin{array}{l} T_{d}\left(x\right)=\{\begin{array}{l} T_{d\_r}\left(x\right)\;x_{sd}\leq x<x_{M}\\ T_{d\_l}\left(x\right)\;x_{0}\leq x<x_{sd} \end{array}\}\;x(t_{i})<x(t_{i-1})\\ T_{a}\left(x\right)=\left\{\begin{array}{l} T_{a\_r}\left(x\right)\;x_{sa}<x\leq x_{u}\\ T_{a\_l}\left(x\right)\;x_{m}<x\leq x_{sa} \end{array}\right\}\;x(t_{i})\geq x(t_{i-1}) \end{array}\right.$$

where $T_{d\_r}(x)$, $T_{d\_l}(x)$, $T_{a\_r}(x)$ and $T_{a\_l}(x)$ are given by Equations (17), (19), (24) and (26), respectively, and replacing *y_m_(t)* by *y_ELAM_(t)* in Equation (1), the *H_ELAM_(p(t))* model for Figure 4 is:

(28) $$H_{ELAM}(p(t))=y_{ELAM}\left(x(t)\right)$$

### 4.2. Inverse External Loop Adaptation Model

The purpose of building a model of the hysteresis of the sensor is that the inverse model can be applied in cascade with the sensor output to compensate its hysteretic behavior. It is, therefore, necessary to invert the ELAM model proposed in this paper in such a way that for any output value of the tactile sensor $y_{out}=T(x_{T})$ we can calculate the input value $x_{T}$. The inverse model construction is performed similarly to the direct model described in the previous section. The following procedure is actually the same that is described in the previous section but with a swap of the coordinate axes. Since the split point is the same, the inverted curves are obtained just inverting the mapping in each piece. The inverted mapping is readily performed from Equations (9) and (10) as follows. Firstly, the output value of the pattern curve *P* that corresponds to the value of the sensor output $y_{out}=T(x_{T})$ is obtained from the Equation (10) as

(29) $$y_{P}=Y_{T}^{-1}(T(x_{T}),y_{it},y_{ft},y_{ip},y_{fp})=y_{iP}+\frac{\left(T(x_{T})-y_{iT})\cdot (y_{fP}-y_{iP}\right)}{\left(y_{fT}-y_{iT}\right)}$$

Then, the value $x_{P}$ is obtained through inverse linear interpolation as $x_{P}=P^{-1}(y_{P})$, and the value of $x_{T}$ is obtained from the Equation (9) as

(30) $$x_{T}=X_{P}^{-1}(x_{P},x_{iT},x_{fT},x_{iP},x_{fP})=x_{iT}+\frac{\left(x_{P}-x_{iP})\cdot (x_{fT}-x_{iT}\right)}{\left(x_{fP}-x_{iP}\right)}$$

The Equations (29) and (30) define the linear mapping for the inverse model ELAM, so the equations of the ascending or descending curves in the inverse model can be obtained from them in a similar way as that followed to build the direct model.

**Figure 8:**
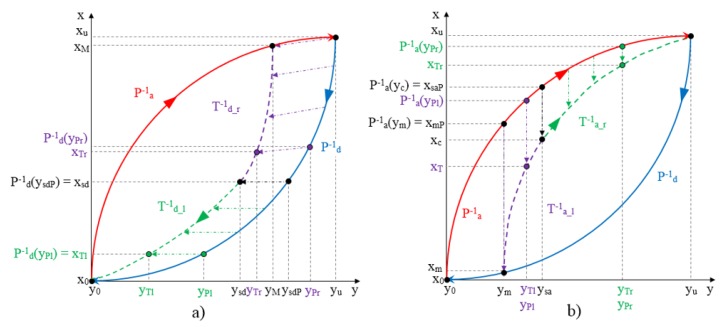
(**a**) Construction of descending curves for inverted ELAM. (**b**) Construction of ascending curves for inverted ELAM.

Figure 8a shows a schematic of the construction of the inverse model for descending curves. These curves, as in the direct model, are determined by the return point $\left(x_{M},y_{M}\right)$ and the split point $\left(x_{sd},y_{sd}\right)$. For input values $y_{sd}\leq y_{Tr}<y_{M}$, the pattern curve $P_{d}^{-1}$ is mapped to the target trajectory $T_{d\_r}^{-1}$. For this process, it is necessary to know the point $y_{sdP}$ such that $P_{d}^{-1}(y_{sdP})=x_{sd}$. Note that since the split point is the same as in the direct model, $y_{sdP}$ can be obtained as $y_{sdP}=P_{d}(x_{sd})$. For each value of $y_{Tr}$ in $T_{d\_r}^{-1}$ there is an associated value $y_{Pr}=P(x_{Pr})$ in $P_{d}^{-1}$ which can be deduced from Equation (29) as

(31) $$y_{Pr}=Y_{T}^{-1}(y_{Tr},y_{sd},y_{M},y_{sdP},y_{u})=y_{sdP}+\frac{\left(y_{u}-y_{sdP})\cdot (y_{Tr}-y_{sd}\right)}{\left(y_{M}-y_{sd}\right)}$$

The value of $T_{d\_r}^{-1}$ is deduced from the Equation (30) knowing that $x_{Pr}=P^{-1}(y_{Pr})$ and its value is

(32) $$x_{Tr}=T_{d\_r}^{-1}(y_{Tr})=X_{P}^{-1}\left(x_{Pr},x_{sd},x_{M},x_{sd},x_{u}\right)=x_{sd}+\frac{\left(x_{M}-x_{sd})\cdot (x_{Pr}-x_{sd}\right)}{\left(x_{u}-x_{sd}\right)}$$

For input values $y_{0}\leq y_{Tl}<y_{sd}$, the $P_{d}^{-1}$ curve is mapped to the $T_{d\_l}^{-1}$ trajectory, so it is necessary to determine the value $y_{Pl}$ in $P_{d}^{-1}$ corresponding to each value of $y_{Tl}$. From Equations (29) and (30) we obtain:

(33) $$y_{Pl}=Y_{T}^{-1}(y_{Tl},y_{0},y_{sd},y_{0},y_{sdP})=\frac{y_{sdP}\cdot y_{Tl}}{y_{sd}}$$

(34) $$x_{Tl}=T_{d\_l}^{-1}\left(y_{Tl}\right)=X_{P}^{-1}(x_{Pl},x_{0},x_{sd},x_{0},x_{sd})=x_{Pl}=P_{d}^{-1}\left(y_{Pl}\right)$$

Figure 8b shows a schematic of the construction of the inverse model for ascending trajectories starting at the return point $\left(y_{m},x_{m}\right)$ and which converge at the point $\left(y_{u},x_{u}\right)$.

For input values $y_{m}<y_{Tl}\leq y_{sa}$, a mapping of the external curve $P_{a}^{-1}$ to the internal trajectory $T_{a\_l}^{-1}$ is performed. To do so, from the general Equations (29) and (30) we obtain:

(35) $$y_{Pl}=Y_{T}^{-1}(y_{Tl},y_{m},y_{sa},y_{mP},y_{saP})=y_{Tl}$$

and

(36) $$x_{Tl}=T_{a\_l}^{-1}\left(y_{Tl}\right)=X_{P}^{-1}(P_{a}^{-1}(y_{Pl}),x_{m},x_{sa},x_{mP},x_{saP})=x_{m}+\frac{\left(P_{a}^{-1}\left(y_{Pl}\right)-x_{mP}\right)\cdot \left(x_{sa}-x_{m}\right)}{\left(x_{saP}-x_{mP}\right)}$$

where the point in the pattern curve which corresponds to each value $y_{Tl}$ in the target curve has the same *y* value and it is called $y_{Pl}=P(x_{Pl})$.

For input values $y_{sa}<y_{Tr}\leq y_{u}$, a mapping of the external curve $P_{a}^{-1}$ is performed along the *y* axis to the internal trajectory $T_{a\_r}^{-1}$. From the general Equations (29) and (30), the resulting expressions are

(37) $$y_{Pr}=Y_{T}^{-1}(y_{Tr},y_{sa},y_{u},y_{saP},y_{u})=y_{Tr}$$

and

(38) $$x_{Tr}=T_{a\_r}^{-1}\left(y_{Tr}\right)=X_{P}^{-1}(P_{a}^{-1}(y_{Pr}),x_{sa},x_{u},x_{saP},x_{u})=x_{sa}+\frac{\left(P_{a}^{-1}(y_{Pr})-x_{saP}\right)\cdot \left(x_{u}-x_{sa}\right)}{\left(x_{u}-x_{saP}\right)}$$

where the point in the pattern curve which corresponds to each value $y_{Tr}$ in the target curve has the same *y* value and it is called $y_{Pr}=P(x_{Pr})$.

Summarizing, the complete inverse ELAM model is expressed as

(39) $$T^{-1}(y)=\left\{ \begin{array}{l} T_{d}^{-1}\left(y\right)=\{\begin{array}{l} T_{d\_r}^{-1}\left(y\right)\;y_{sd}\leq y<y_{M}\\ T_{d\_l}^{-1}\left(y\right)\;y_{0}\leq y<y_{sd} \end{array}\}\;y(t_{i})<y(t_{i-1})\\ T_{a}^{-1}\left(y\right)=\left\{\begin{array}{l} T_{a\_r}^{-1}\left(y\right)\;y_{sa}<y\leq y_{u}\\ T_{a\_l}^{-1}\left(y\right)\;y_{m}<y\leq y_{sa} \end{array}\right\}\;y(t_{i})\geq y(t_{i-1}) \end{array}\right.$$

## 5. Parameter Identification

The four methods explained in the previous section are used to model hysteresis loops (see Figure 2a,b) of the tactile sensor. As a step prior to the construction, the validation and the comparison of the proposed models, it is necessary to carry out the identification of the parameters to adapt the models to the experimental data as accurately as possible. Many identification algorithms have been proposed for this purpose such as the least squares method, genetic algorithms and the particle swarm optimization method [40,41,42]. In this paper, we use genetic algorithms because they implement a parallel procedure able to simultaneously explore a wide range of solutions using probabilistic operators [43]. This feature allows them to discard local minima that do not correspond to an optimal solution. In the following, the different methods to obtain the parameters for the results in Section 6 are described.

### 5.1. GPI

The GPI model obtained from Equation (2) is completely defined by the number of generalized play operators used, the thresholds of the operators, the density function and the envelope functions.

Regarding of the number of operators *n*, from a theoretical point of view, the selection of a larger number of operators should obtain a more accurate approximation of the hysteresis loops. However, in real applications, it is found that further increase of the number of operators does not improve the fitting accuracy significantly. Since the complexity of the model is increased with the number of operators employed, it is advisable to use the smallest number of operators. According to the experiments reported in [44] for the same tactile sensor of this paper, it is enough to use a number of *n* = 4 play operators to obtain a good approximation.

For the results of this paper, the thresholds of the operators and the density function are obtained from [28] and their equations are

(40) $$r_{i}=\alpha \cdot i$$

where *i =* 0,1,2*,…,n* and α is a positive real constant, and

(41) $$p(r)=\rho \cdot e^{-\tau \cdot r_{i}}$$

where *ρ* > 0 and τ are real constants. 

Note that the density function *p(r)* vanishes for high values of the thresholds *r* and that there is no general criterion for its selection. Generally, it is completely selected by the designer. Once the structure of the density function *p(r)* is fixed, the parameters involved in the density function shall be determined by identification from experimental data.

The choice of the envelope functions used in the Equation (3) is decisive for a good fitting. Based on the results reported by other studies [44], which analyze and compare the use of different envelope functions, these functions have been chosen according to the following expressions in this paper:

(42) $$\gamma_{l}(x)=a_{3}-a_{0}\cdot e^{\left(a_{2}-a_{1}\cdot x\right)}$$

(43) $$\gamma_{r}(x)=b_{3}-b_{0}\cdot e^{\left(b_{2}-b_{1}\cdot x\right)}$$

where $a_{0}>0,\;a_{1}>0,\;a_{2},a_{3},\;b_{0}>0,\;b_{1}>0,\;b_{2},b_{3}\;$ are real constants.

These envelope functions based on exponential functions provide a good fitting with a medium computational complexity compared to other alternatives such as those based on hyperbolic tangent functions.

### 5.2. MPI

The MPI model is also based on play operators, but of classical type in this case. Therefore, as with the model GPI, it will be necessary to select a number of operators *n* to use, thresholds for such operators, and a density function. The selection criteria for these items in the GPI model are also valid for the MPI model. In this article, we chose these elements according to what is proposed in [30], so a number of 10 classical play operators is selected. The expressions of the threshold operators *r_i_* and the density function *p(r)* are

(44) $$r_{i}=\frac{i-1}{n}{\left\|v(t)\right\|}_{\infty }$$

with *i =* 1,2,…,*n* and ${\left\|v(t)\right\|}_{\infty }=1$ in the normalized case, and

(45) $$p(r_{i})=\rho \cdot e^{-\tau \cdot {\left(r_{i}-1\right)}^{2}}$$

where *ρ* > 0 and *τ* are real constants.

To achieve the asymmetry observed in the hysteresis loop of the tactile sensor, it is necessary to define the generalized function *g(x(t))* of the Equation (5). According to the proposal option in [30], this generalized function is chosen as a third-order polynomial

(46) $$g(x(t))=a_{3}\cdot x^{3}+a_{2}\cdot x^{2}+a_{1}\cdot x$$

The selection of the generalized input function is not unique and other forms can also be chosen, but the use of a third-order polynomial seems a good choice to approximate curves of different shapes. Furthermore, third-degree polynomials are generally recognized as an effective choice to describe the hysteresis loops [45,46]. Actually, this polynomial is the one that achieves the best fitting to the hysteresis curve of the tactile sensor in this paper.

### 5.3. POLY

Regarding the POLY model, the only necessary parameter identification corresponds to the choice of coefficients of the polynomials *f_ra_* and *f_rd_*. Although third-order polynomials are proposed in [31] to be used in the model, sixth-order polynomials have been selected for the results of this paper. Therefore, the polynomials are

(47) $$f_{ra}(x)=\sum_{i=0}^{6}a_{i}\cdot x^{6-i}$$

(48) $$f_{rd}(x)=\sum_{i=0}^{6}b_{i}\cdot x^{6-i}$$

Better results were achieved with tenth-order polynomials, but the choice of sixth-order polynomials seems most appropriate taking into account the balance between precision and the number of parameters required.

### 5.4. ELAM

The identification process for the ELAM model consists of determining the α_d0_, β_d0_, α_a1_, α_a0_ and β_a0_ parameters that allow the split points to be obtained in the Equations (14), (15), (21) and (22), respectively. The number of parameters to be identified is much lower than in the other models, so this identification process has a lower computational cost and the time employed is significantly shorter in this model.

It is remarkable that the GPI, MPI and POLY models require a prior step to choose the suitable envelope functions or polynomials to be used in the construction of the hysteresis loops. Moreover, in the case of models based on the Prandtl-Ishlinskii method, it is necessary to select the number of play operators and the structure of the density function. All this work is performed by the designer based on the results obtained in a selection process in order to find the best fit to the experimental data. Depending on the complexity of the hysteresis curve of the sensor, this process may be more or less costly. The proposed ELAM model does not require this previous step, because the pattern curves are obtained by linear interpolation of experimental data. In addition, the model provides the output from piecewise linear mapping of the pattern curves. Therefore, if the number of pieces to perform linear mapping or interpolation grows, the error always decreases and the model is robust against overfitting [47].

The parameters identified for each model that allow the best adaptation of the models to hysteresis data obtained experimentally for the descending and ascending curves are shown in Table 2 and Table 3, respectively (see Figure 2a,b).

**Table 2:** Parameters of the models for the descending hysteresis curves of the tactile sensor.

GPI				MPI		POLY				ELAM	
*n*	4	*b*_1_	0.0825	n	10	*a*_0_	1.36e^-9^	*b*_0_	9.04e^−10^	*α_a_*_1_	−0.0072
*a*_0_	0.5754	*b*_2_	−1.9648	ρ	1.6492	*a*_1_	−2.74e^-7^	*b*_1_	−1.44e^−7^	*α_a_*_0_	0.6031
*a*_1_	0.0524	*b*_3_	0.2972	τ	0.1942	*a*_2_	2.13e^-5^	*b*_2_	6.93e^−6^	*β_a_*_0_	0.3382
*a*_2_	1.1216	α	1.7531	*a*_1_	3.96e^−5^	*a*_3_	−7.69e^-4^	*b*_3_	5.29e^−6^	*α_d_*_0_	0.7957
*a*_3_	1.6350	ρ	4.4935	*a*_2_	0.1410	*a*_4_	0.0109	*b*_4_	−0.0100	*β_d_*_0_	0.9991
*b*_0_	13.0421	τ	1.5428	*a*_3_	−0.0049	*a*_5_	0.0704	*b*_5_	0.3132		
						*a*_6_	−0.0244	*b*_6_	−0.1864		

**Table 3:** Parameters of the models for the ascending hysteresis curves of the tactile sensor.

GPI				MPI		POLY				ELAM	
*n*	4	*b*_1_	0.1168	n	10	*a*_0_	1.65e^−9^	*b*_0_	1.20e^−9^	*α_a1_*	−0.0072
*a*_0_	1.9620	*b*_2_	−0.1206	ρ	3.0226	*a*_1_	−3.37e^−7^	*b*_1_	−2.08e^−7^	*α_a0_*	0.6031
*a*_1_	0.0477	*b*_3_	−3.2440	τ	0.2255	*a*_2_	2.66e^−5^	*b*_2_	1.21e^−5^	*β_a0_*	0.3382
*a*_2_	−0.1324	α	3.4384	*a*_1_	3.68e^−5^	*a*_3_	−9.93e^−4^	*b*_3_	−2.05e^−4^	*α_d0_*	0.7957
*a*_3_	3.7234	ρ	0.8893	*a*_2_	0.1502	*a*_4_	0.0157	*b*_4_	−0.0057	*β_d0_*	0.9991
*b*_0_	1.6743	τ	−0.1617	*a*_3_	−0.0047	*a*_5_	0.0281	*b*_5_	0.2762		
						*a*_6_	−0.0108	*b*_6_	−0.1651		

## 6. Results and Discussion

### 6.1. Results for the Output of a Single Average Taxel

The hysteresis loop in Figure 2a,b is really challenging to model because it is clearly asymmetrical, with high nonlinearity, and quite different ascending and descending external curves. Furthermore, the ascending curves inside the hysteresis loop do not have a similar shape to the external ascending curves, as can be seen in Figure 2b.

**Figure 9:**
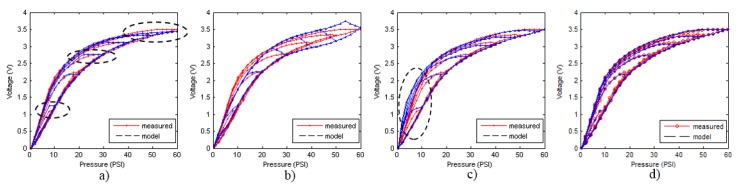
Models for descending curves. (**a**) GPI model with n = 4 play operators; (**b**) MPI model with third-order polynomial and n = 10 OSP operators; (**c**) POLY model with sixth-order polynomial; (**d**) ELAM model.

**Figure 10:**
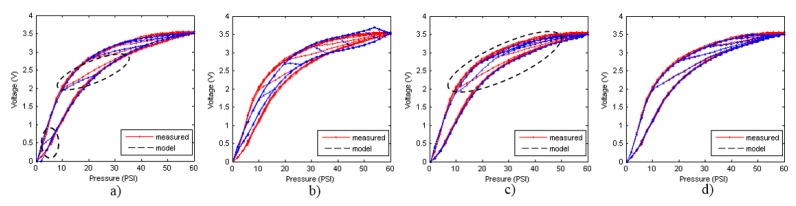
Models for ascending curves. (**a**) GPI model with n = 4 play operators; (**b**) MPI model with third-order polynomial and n = 10 OSP operators; (**c**) POLY model with sixth-order polynomial; (**d**) ELAM model.

Figure 9a–d and Figure 10a–d show the models of the descending and ascending hysteresis curves, respectively obtained with the four methods implemented in this work. In addition, Table 4 and Table 5 show the values of the average and maximum errors for each model with respect to the experimental data, both in absolute value and in percentage of the full scale. The error is evaluated according to the following expression.

(49) $$error=\frac{1}{N}\cdot \sum_{i=1}^{N}\left|\left(y_{m\_i}-y_{i}\right)\right|$$

where *y_m_i_* are the model samples, *y_i_* are the experimental samples and *N* is the number of samples. The column labeled *Best* refers to the minimum error value obtained after *J(X)* in Equation (1) is minimized during the parameter identification process.

**Table 4:** Average errors and maximum errors of hysteresis models for descending curves.

Model	Best	Average Error (V)	Average Error % FS	Max. Error (V)	Max. Error % FS
GPI	0.53	0.04	1.15	0.15	4.34
MPI	2.82	0.09	2.63	0.35	10.05
POLY	4.14	0.07	1.99	0.70	19.98
ELAM	0.33	0.031	0.89	0.12	3.57

The first thing that can be observed in Figure 9 and Figure 10 is that the worse fit to the experimental data was obtained with the MPI model. This model, as explained in Section 3.2, consists of a set of symmetric classical play operators and a generalized function to model the asymmetry of the hysteresis loop. It is very difficult to find a single generalized function that fit to both external curves efficiently. Tests have been done with polynomial of different degrees, and the best result was achieved with the third-order polynomial in Equation (46). Since the results from this model are not good for the hysteresis curves in Figure 2, probably because classical play operators were primarily intended for adjustment of symmetrical loops, no further comment will be made about it.

**Table 5:** Average errors and maximum errors of hysteresis models for ascending curves.

Model	Best	Average Error (V)	Average Error % FS	Max. Error (V)	Max. Error % FS
GPI	1.21	0.04	1.12	0.30	8.47
MPI	7.45	0.10	2.94	0.35	9.77
POLY	2.27	0.06	1.64	0.22	6.15
ELAM	0.37	0.020	0.58	0.11	3.16

The GPI model shows a very good performance for the descending curves, although some significant deviations are observed and circled in Figure 9a. The model is saturated for high values of the input signal, so that the adjustment in this area is not good. This behavior is repeated at the start of each inner descending curve. Regarding the ascending curves, the model shows a poor performance in fitting them, as Figure 10a depicts. The main reason behind these deviations is the difficulty in finding envelope functions that fit precisely to the external curves in the complete input range.

With respect to the POLY model, it shows a great difficulty in adjusting the descending curves (see Figure 9c). It is noted that the descending curves of the model are shifted to the left with respect to the experimental data. This is because it is not possible to find the polynomial in Equation (48) to fit the external descending curve of the tactile sensor. As for the ascending curves (see Figure 10c), the model cannot adjust the internal curves well for the same reason.

The ELAM models for the ascending and descending curves, are shown in Figure 9d and Figure 10d respectively. It can be observed that the adjustment of the descending curves is better than with the other methods. The curves do not saturate as with the GPI model (see Figure 9a). The split point introduced in the ELAM model provides a larger flexibility than that of the POLY model to approximate highly nonlinear curves. Regarding the ascending curves, the ELAM model is also the one that achieves the best fitting, even in the internal ascending branches, where other models have large errors.

**Figure 11:**
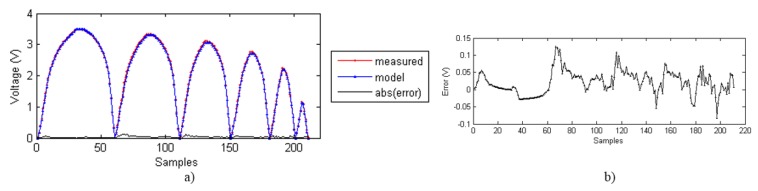
(**a**) Measured data, ELAM model and absolute value of the error; (**b**) Error zoom.

**Figure 12:**
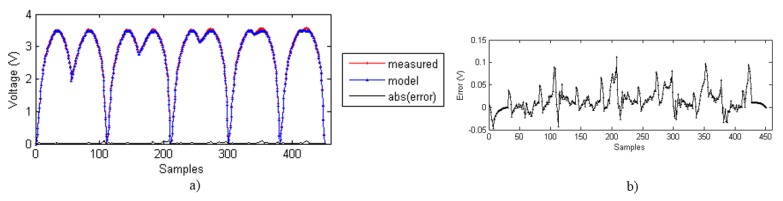
(**a**) Measured data, ELAM model and absolute value of the error; (**b**) Error zoom.

Figure 11a shows the output voltage from samples measured and obtained with the ELAM method for different descending trajectories (see Figure 2a), as well as the error, whose zoom is displayed at Figure 11b. Figure 12 shows the same data for different ascending curves (see Figure 2b). The error is below 0.1% of the full scale sensor output in both cases. Therefore, the ELAM method provides the model that achieves the best fitting of the experimental data. The samples are the output voltage values obtained every two seconds. The number of measured samples is 211 for the descending curves and 451 for the ascending curves.

Furthermore, regarding the interest stated in the introduction in implementing the compensation algorithms in the local electronics based on an FPGA, the ELAM model is the most suitable because its complexity is similar to the POLY model but lower than that of the other two models based on Prandtl-Ishlinskii operators. Specifically, the GPI model shows a fit to the experimental curves close to that achieved with the ELAM model, but the mathematical operators involved are much more complex. The ELAM method is based on simple mathematical operations such as additions and multiplications, while the GPI and MPI models use exponential functions for the density function and for the envelope functions. These operations are more difficult to implement and require the use of more logical resources in devices such as FPGAs. The parameter identifying process in Section 5 is also simpler in the ELAM method. Finally, the GPI, MPI and POLY methods require of a prior selection step of the functions to be used in the construction of the models, while the ELAM method does not need it because it uses the experimental data for the construction of the hysteresis loops. Only the way to obtain the split point has to be determined, and simple linear (see Equations (11) and (12)) or quadratic (see Equation (21)) expressions provide good results.

**Figure 13:**
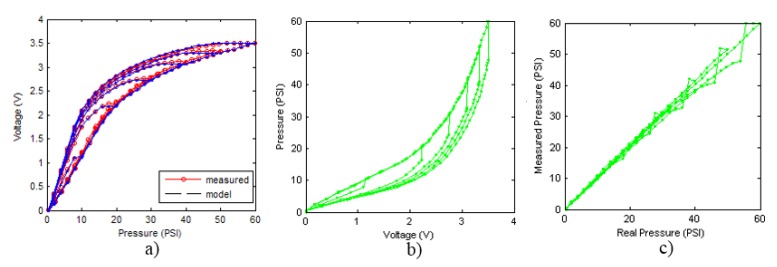
Compensation for descending curves with ELAM method (**a**) ELAM direct model; (**b**) ELAM inverse model; (**c**) Sensor output pressure *versus* real pressure after ELAM compensation.

**Figure 14:**
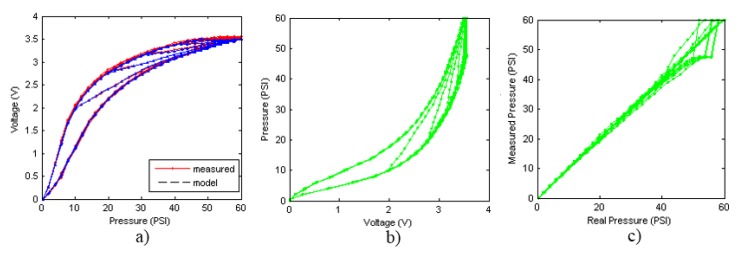
Compensation for ascending curves with ELAM method (**a**) ELAM direct model; (**b**) ELAM inverse model; (**c**) Sensor output pressure *versus* real pressure after ELAM compensation.

Therefore the ELAM model is inverted according to equations set out in Section 3.5 and it is used for compensating hysteresis nonlinearities presented by the experimental curves of Figure 2a,b. Figure 13 shows the output of the sensor for a set of loading-unloading cycles when it is compensated with the ELAM method. Figure 13a shows the direct ELAM model and the curves measured experimentally. Figure 13b displays the inverse ELAM model, wherein the pressure calculated by the model associated to the sensor output voltage is represented. Figure 13c displays the pressure calculated by the ELAM model *versus* the real exerted pressure, *i.e.*, it shows the compensation of the descending cycles of the sensor hysteresis. The same data are shown in Figure 14 for the ascending curves. It is noteworthy that the performance of the ELAM model has been assessed from experimental data instead of using other simulated input that could provide artificially good results. In this respect, the errors observed in Figure 13c and Figure 14c are due to noise in the flat areas of the experimental curves (note that the descending curves are saturated for high input pressure values). These errors limit the useful range of the sensor for a given resolution of the measurement.

### 6.2. Results with Tactile Sensor Matrix

This section shows the application of the ELAM method to compensate the hysteresis of a tactile sensor composed of 256 taxels distributed in 16 rows and 16 columns. Each taxel is an independent sensing unit that must be modeled individually, and its hysteresis nonlinearity has to be compensated with its ELAM model. Table 6 shows the frames obtained for different uniform pressures exerted on the sensor. A large mismatching between taxels is observed. Two frames measured for the same input pressure are compared in the same column of the table, one for an ascending sequence (top) and the other for a descending sequence (bottom). Table 7 shows the same frames once the compensation with the ELAM method is applied. It can be observed that not only the hysteresis nonlinearities but also the mismatching between different taxels are compensated. The value of the relative standard deviation with respect to the full scale output is shown for each frame to quantify the improvement regarding the mismatching after the compensation process. In addition, the difference between the frames in the ascending and descending paths is displayed, and its mean value with respect to the full scale output illustrates the improvement regarding the hysteresis.

**Table 6:** Hysteresis loop frames measured before compensation.

UP ➔	4.01 PSI	10.25 PSI	20.20 PSI	30.06 PSI	40.21 PSI	49.93 PSI	59.93 PSI
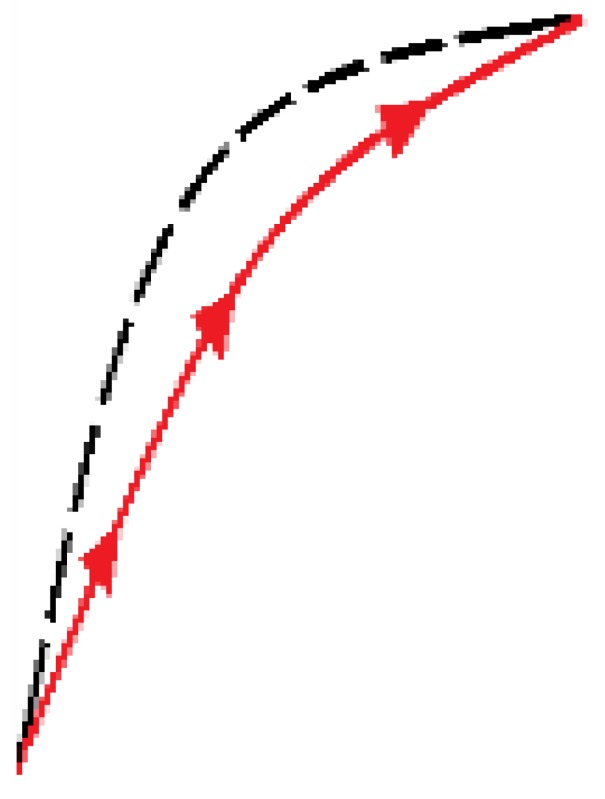	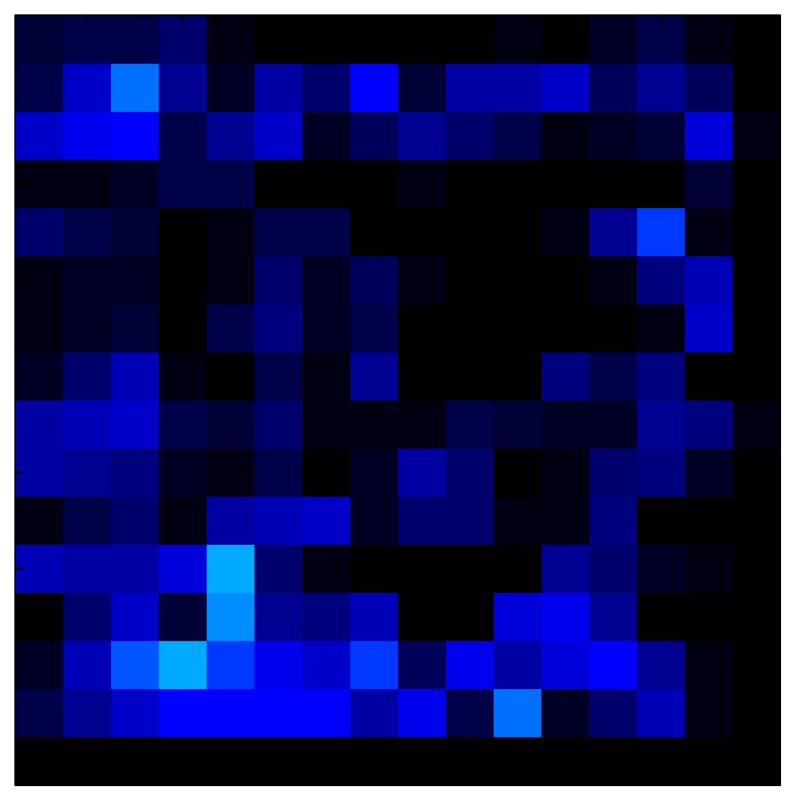	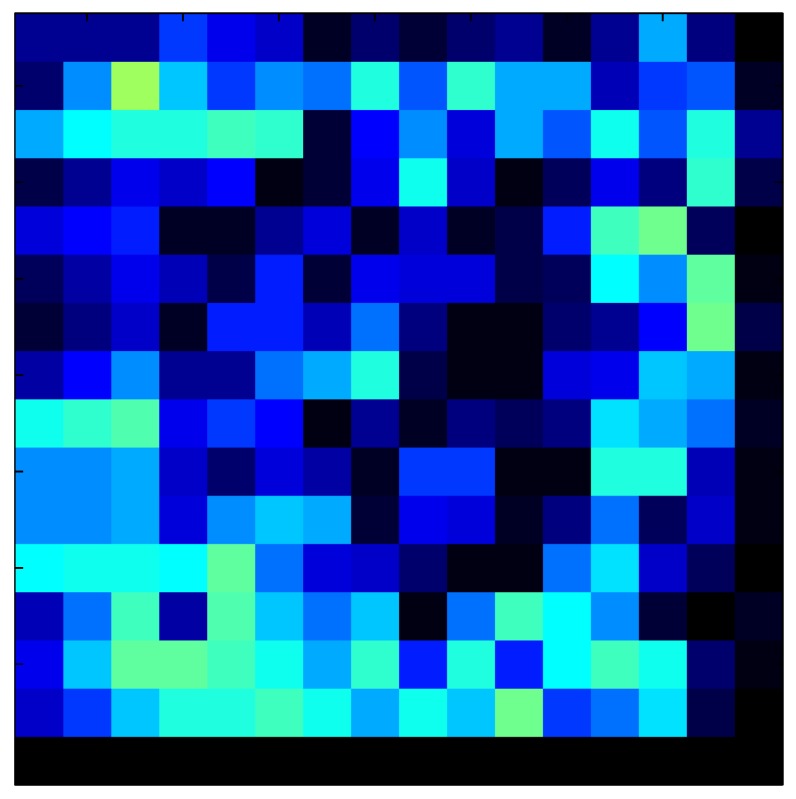	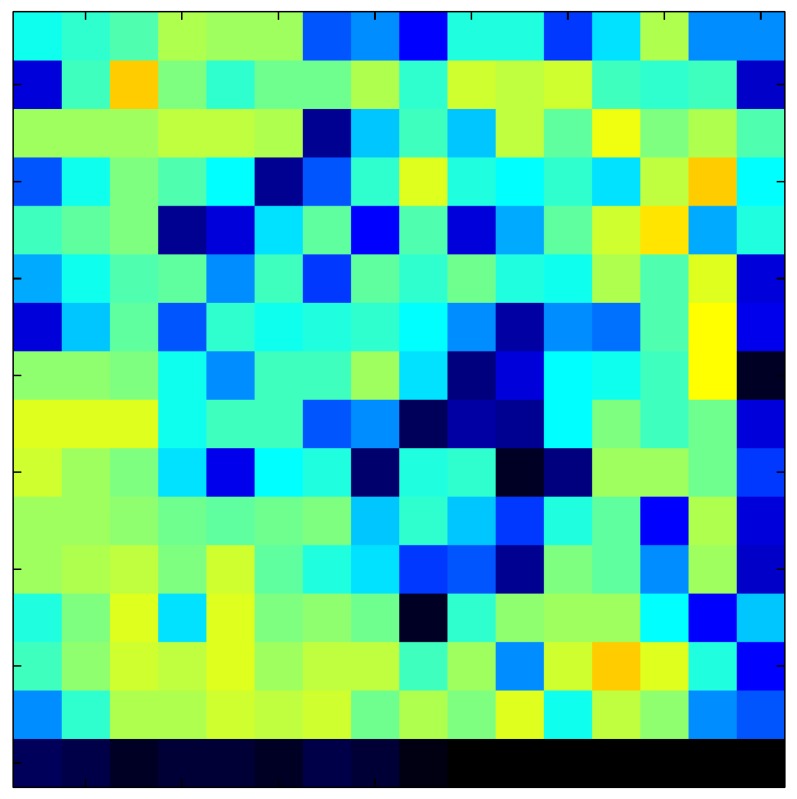	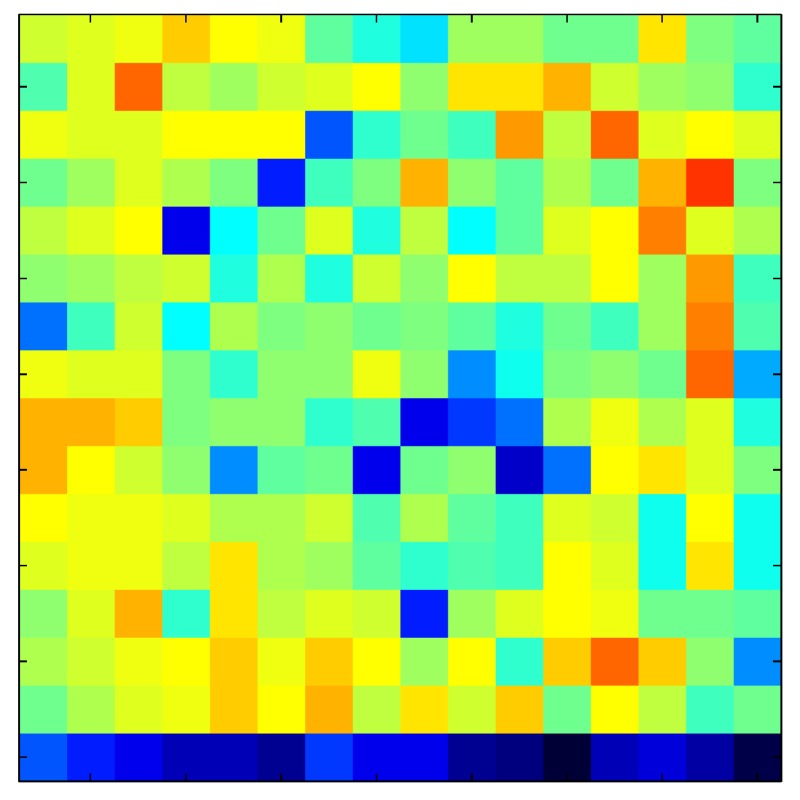	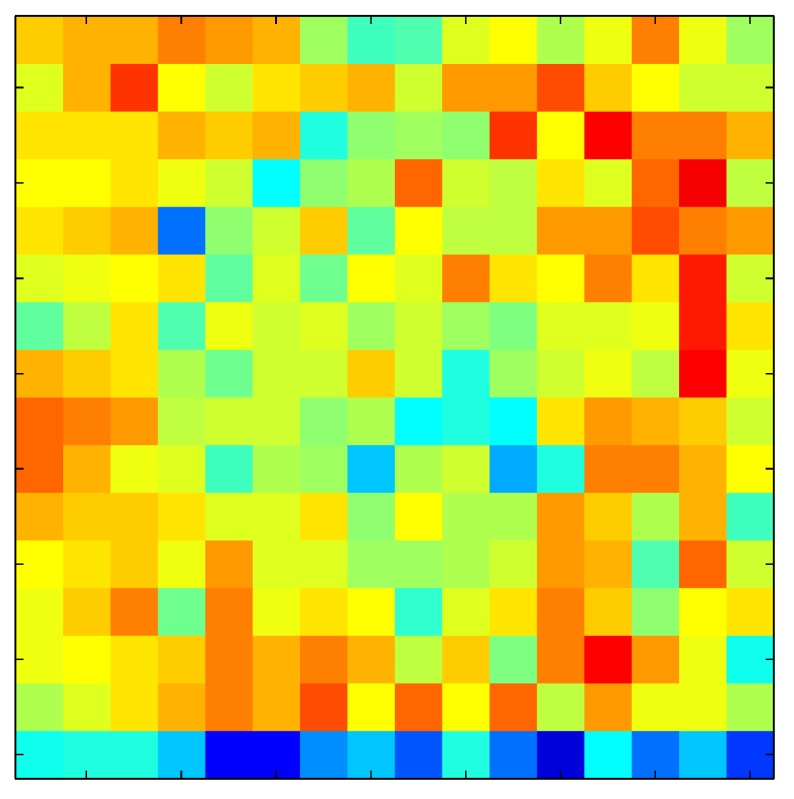	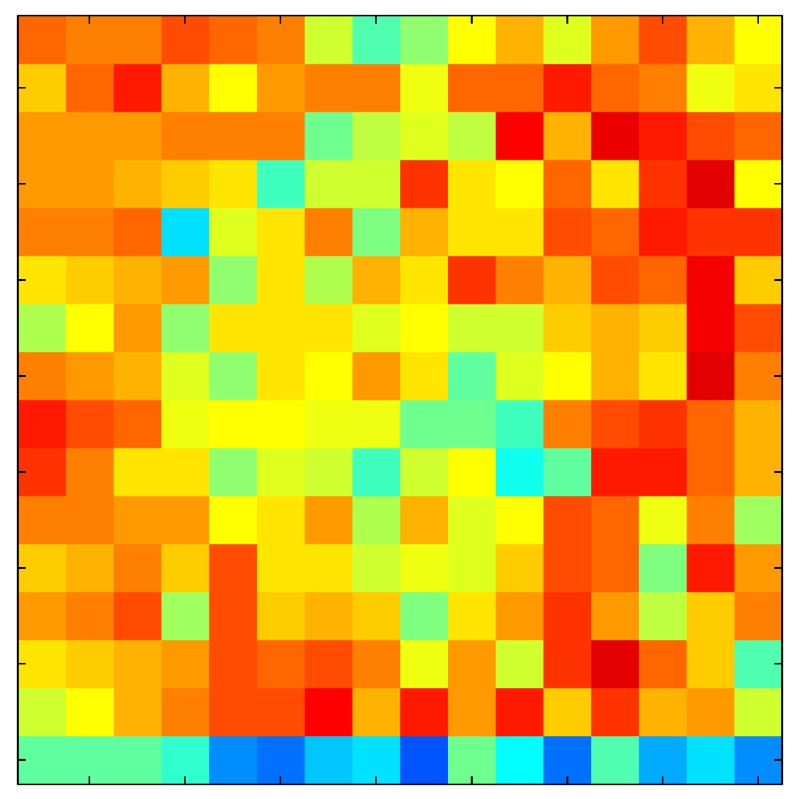	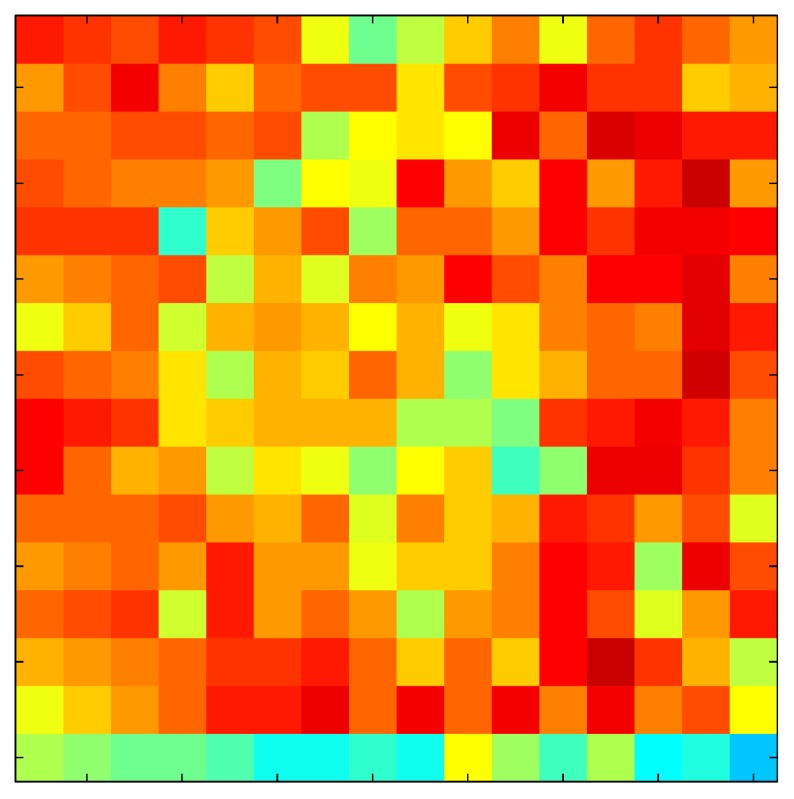
std(x)/FS:	0.09	0.16	0.18	0.15	0.13	0.12	
DOWN ➔	4.01 PSI	10.05 PSI	19.86 PSI	30.01 PSI	40.02 PSI	49.59 PSI	
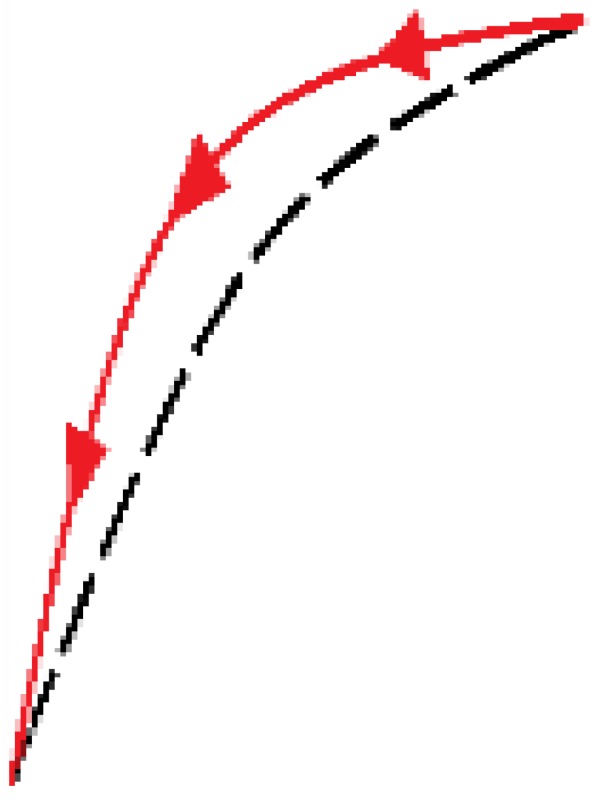	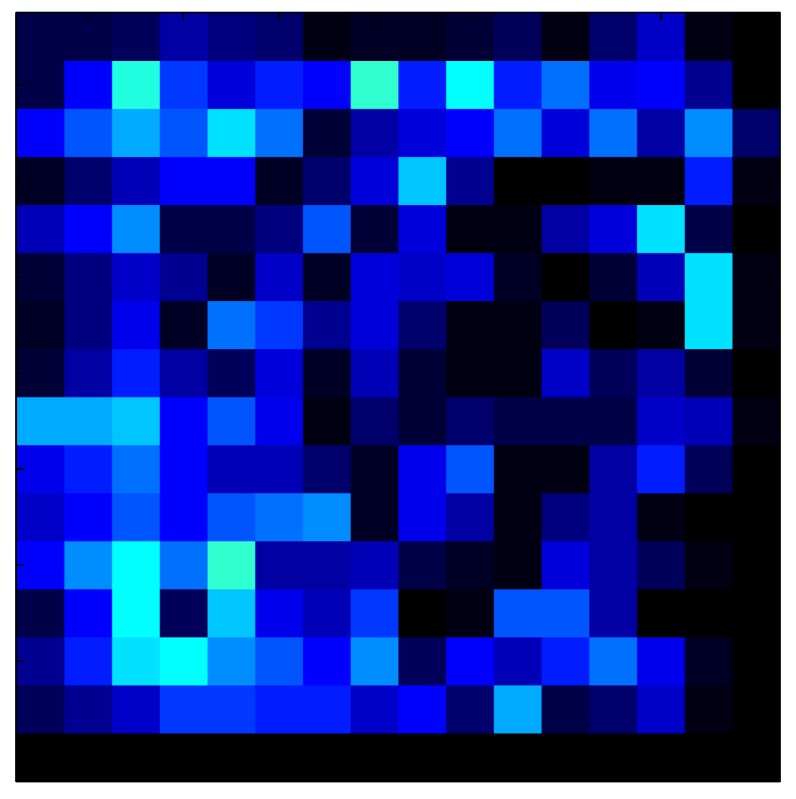	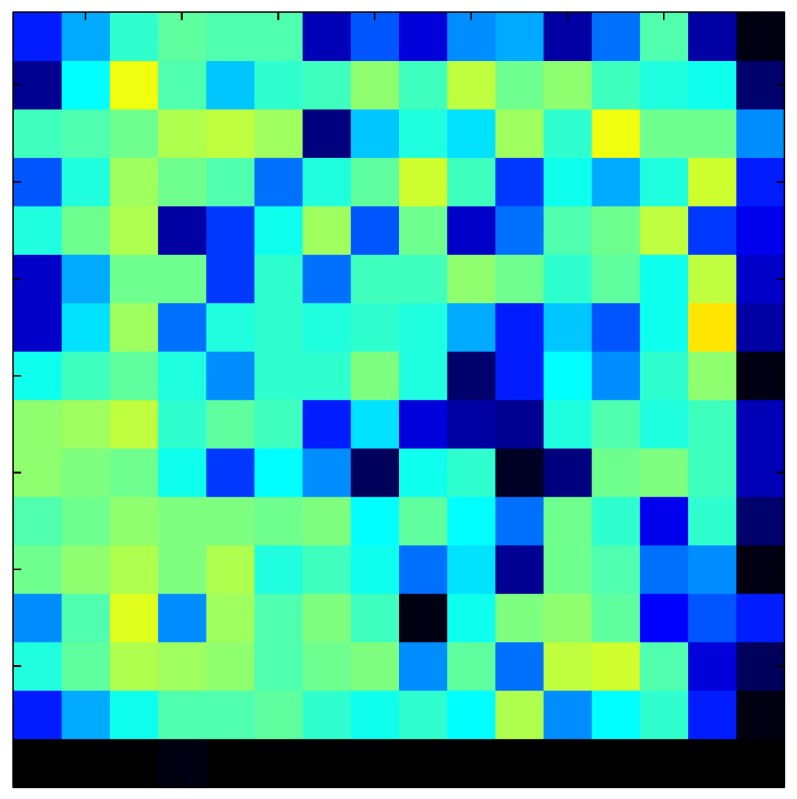	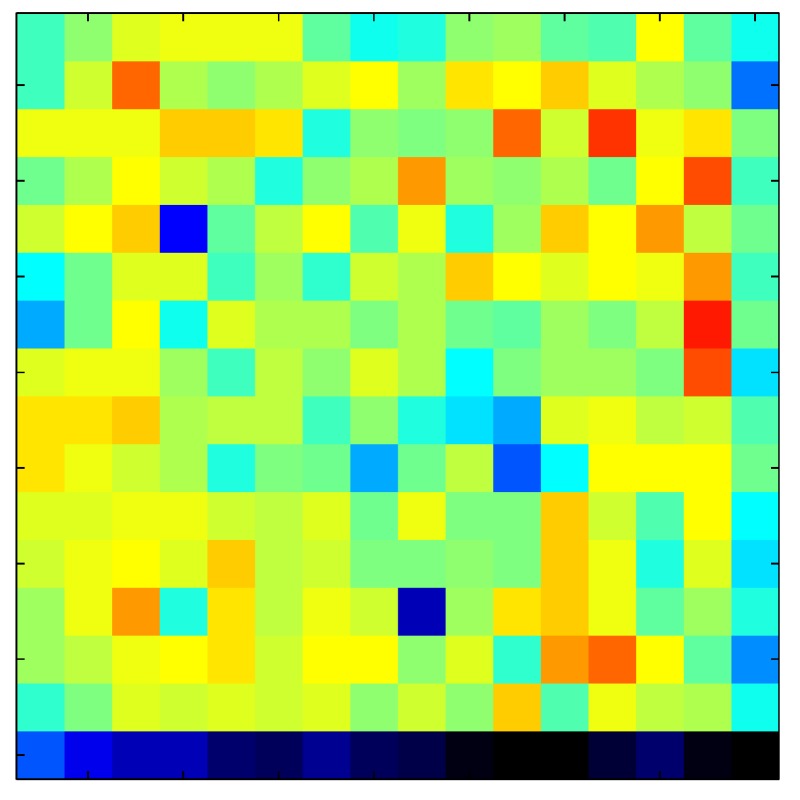	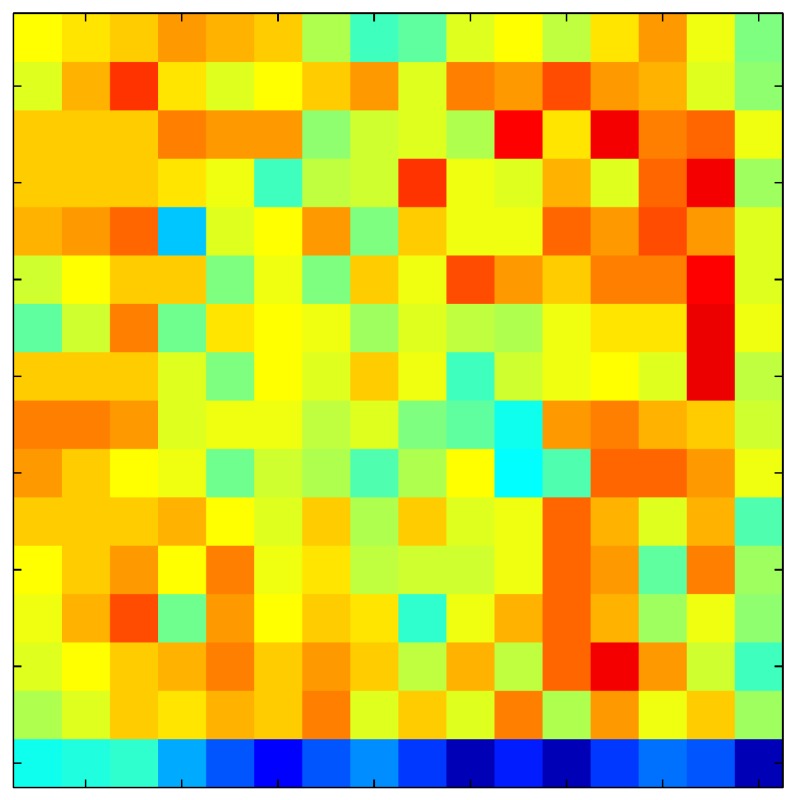	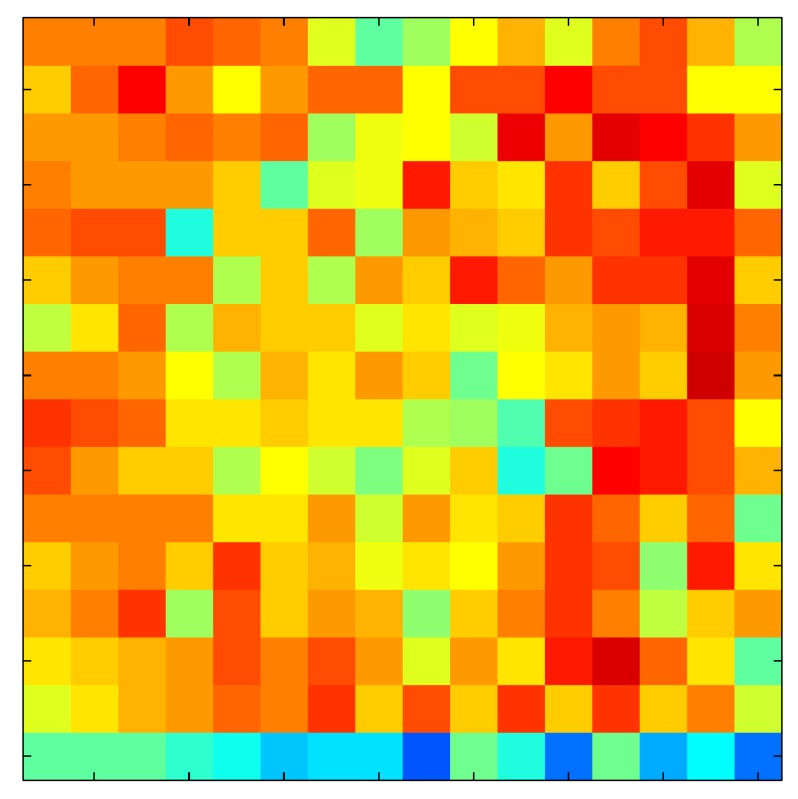	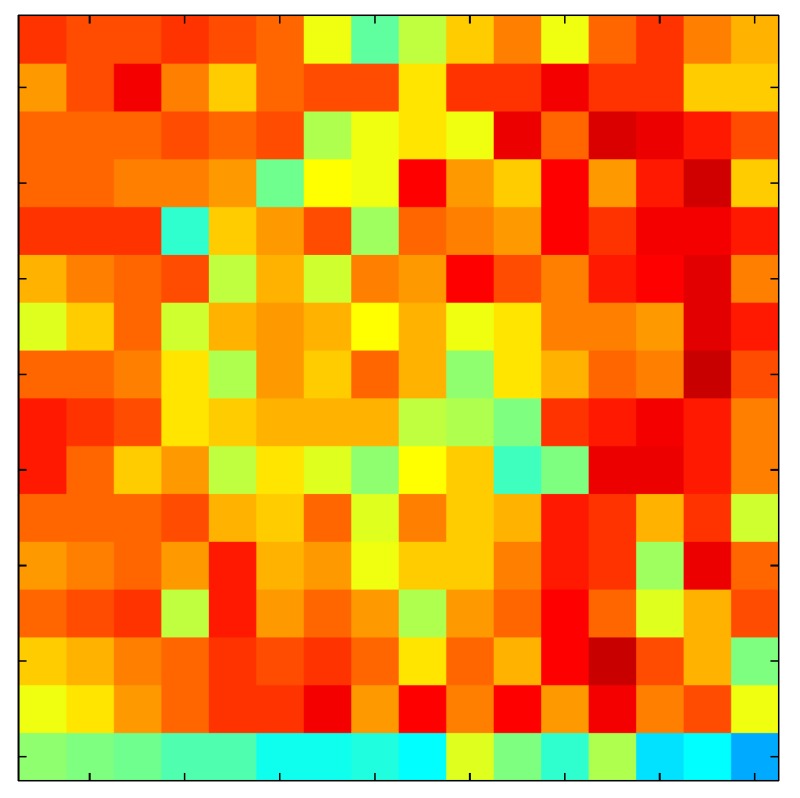	
std(x)/FS:	0.13	0.18	0.16	0.13	0.12	0.11	0.11
Absolute Distance	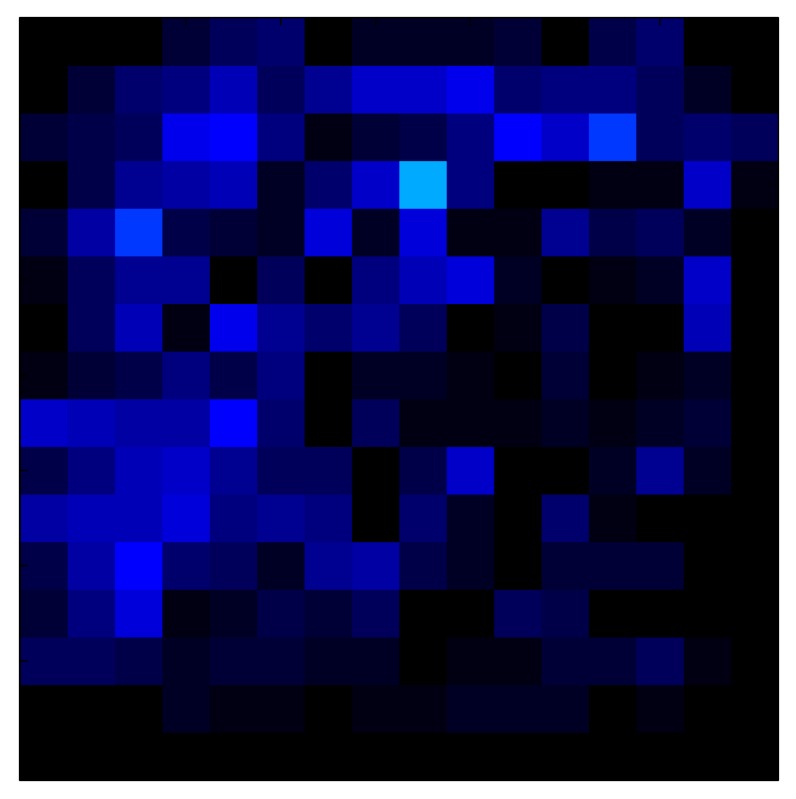	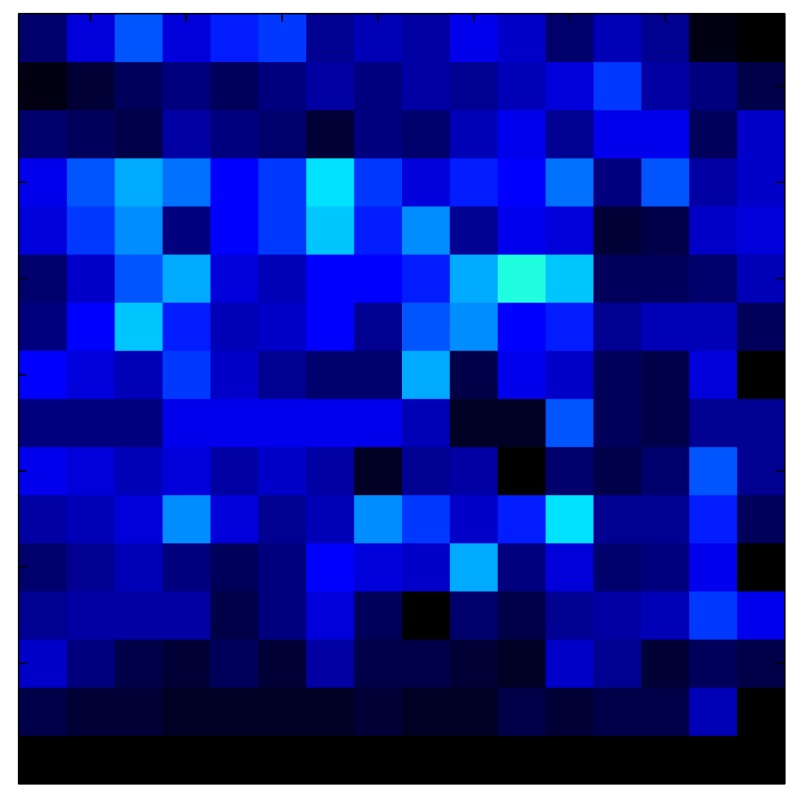	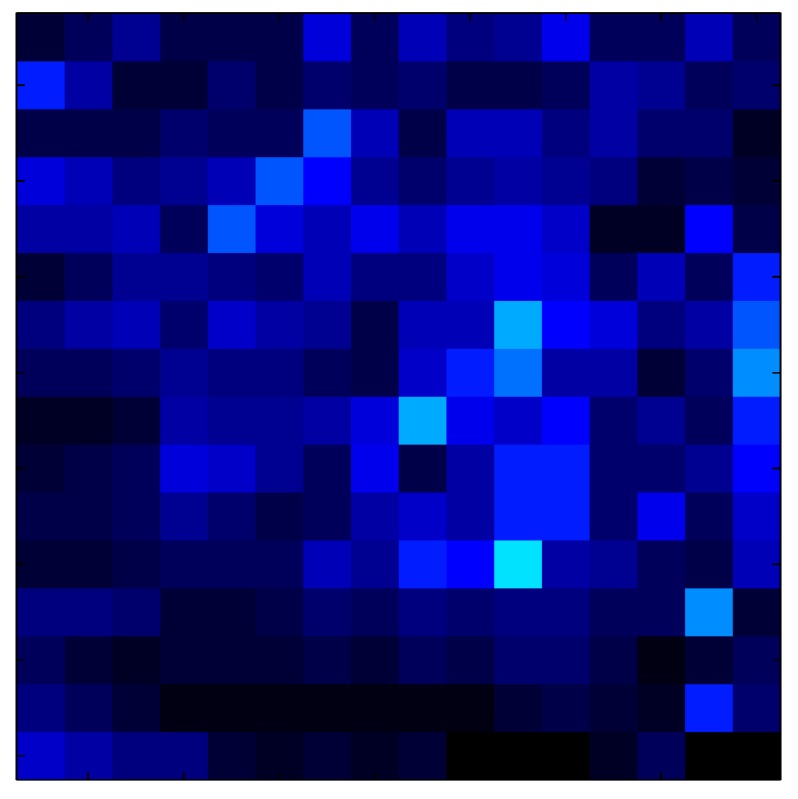	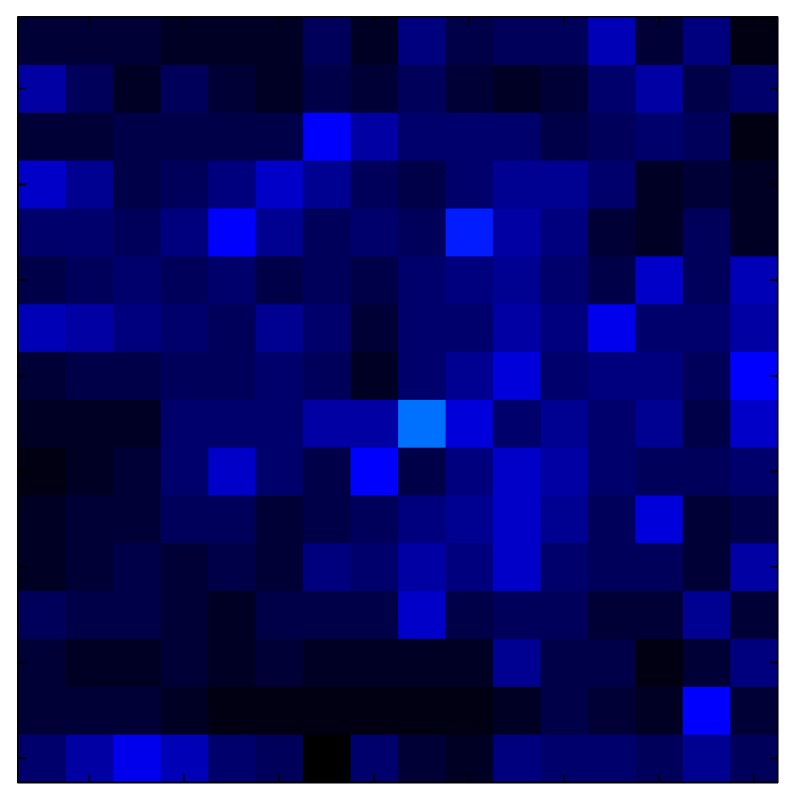	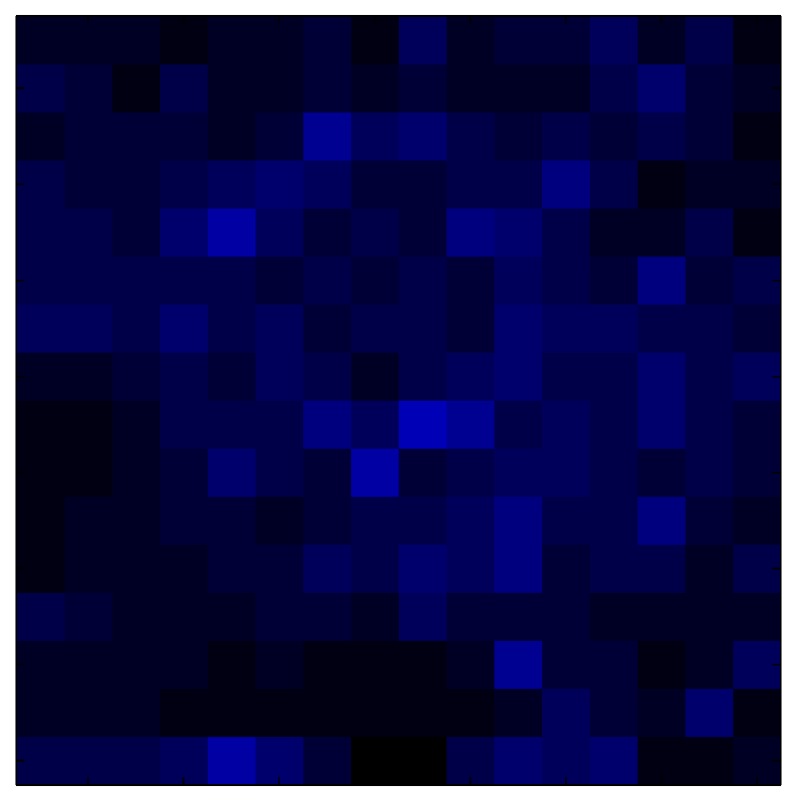	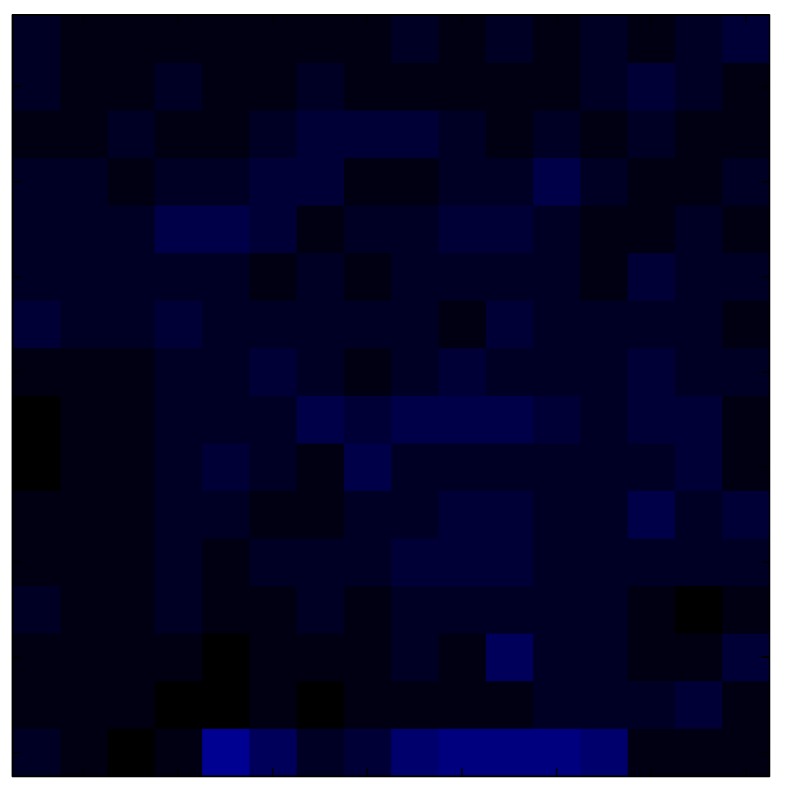	
:$\bar{x}/\mathrm{FS}$:	0.08	0.17	0.14	0.11	0.07	0.04	

**Table 7:** Hysteresis loop frames after compensation with ELAM method.

UP ➔	4.01 PSI	10.25 PSI	20.20 PSI	30.06 PSI	40.21 PSI	49.93 PSI	59.93 PSI
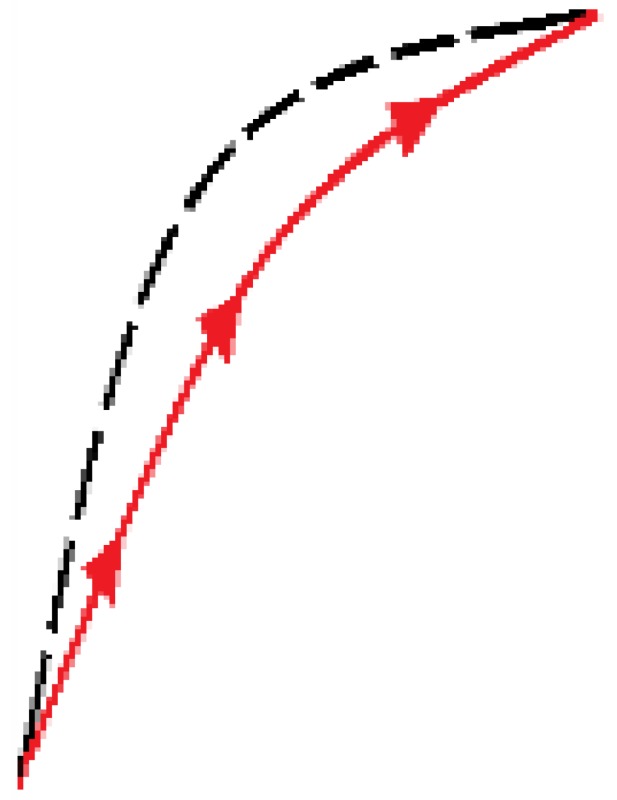	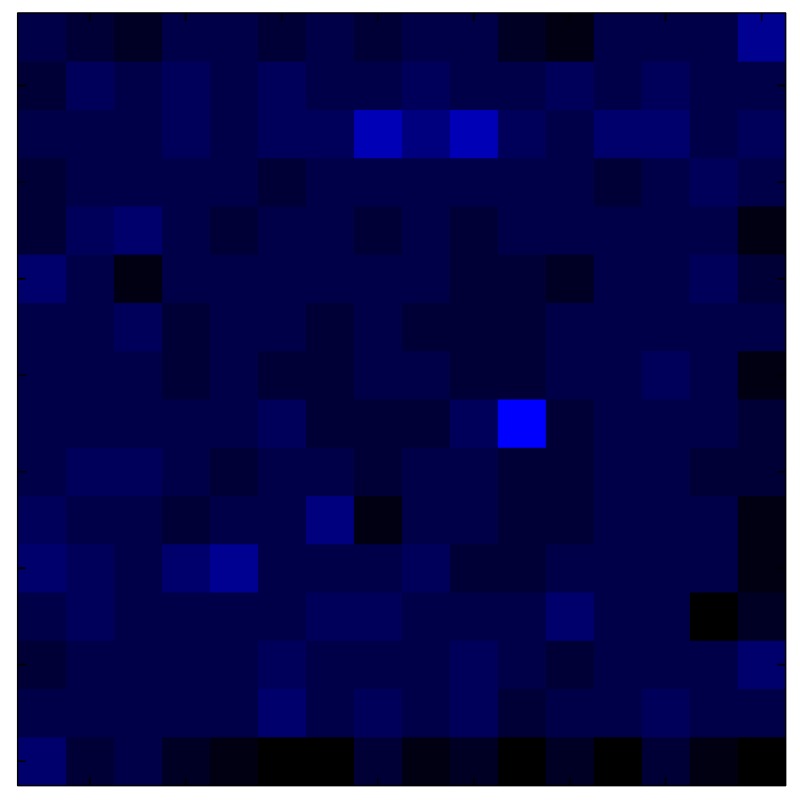	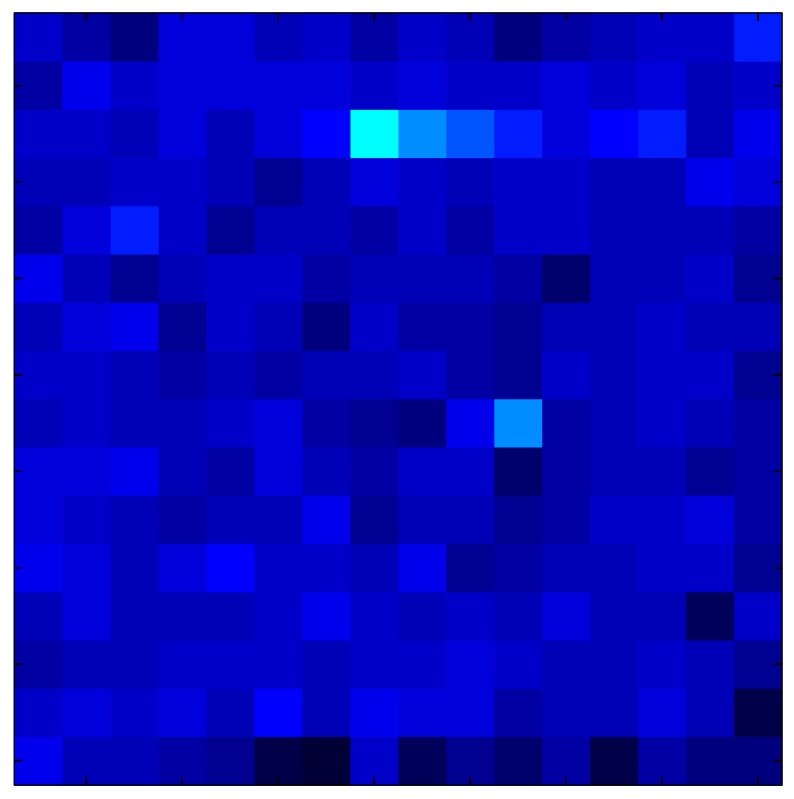	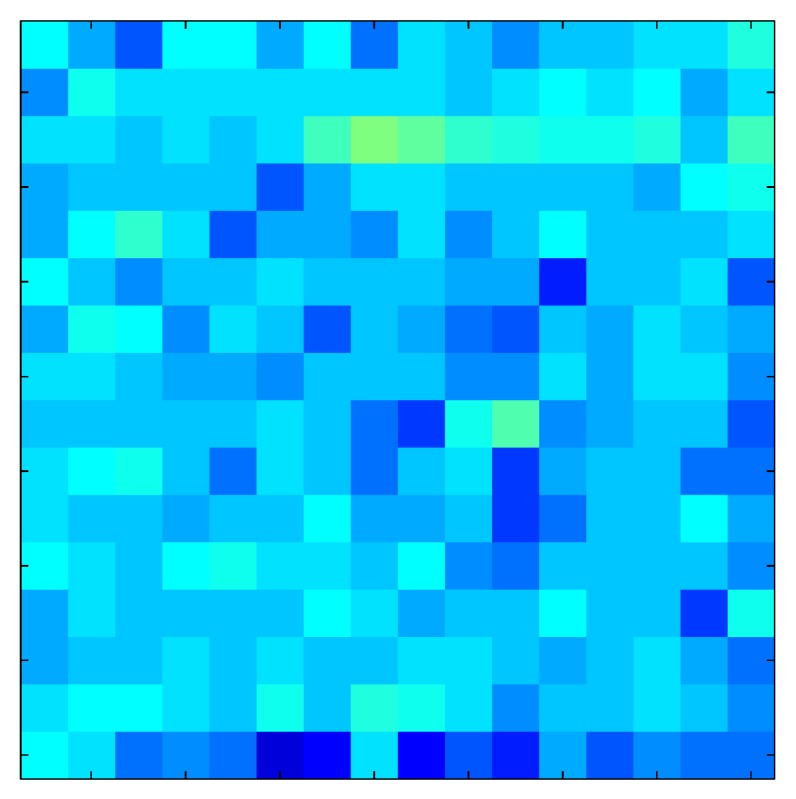	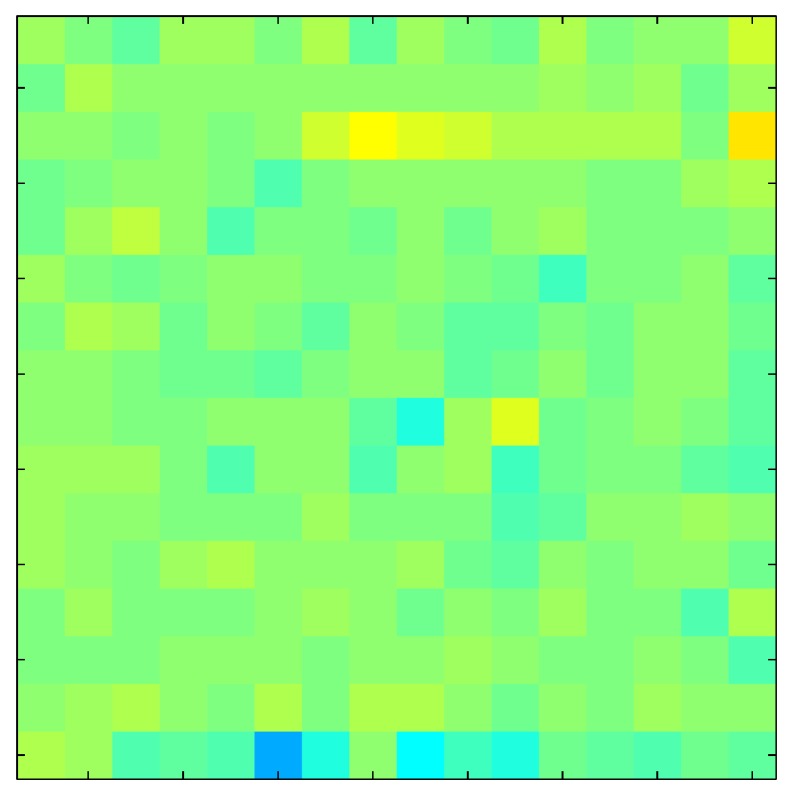	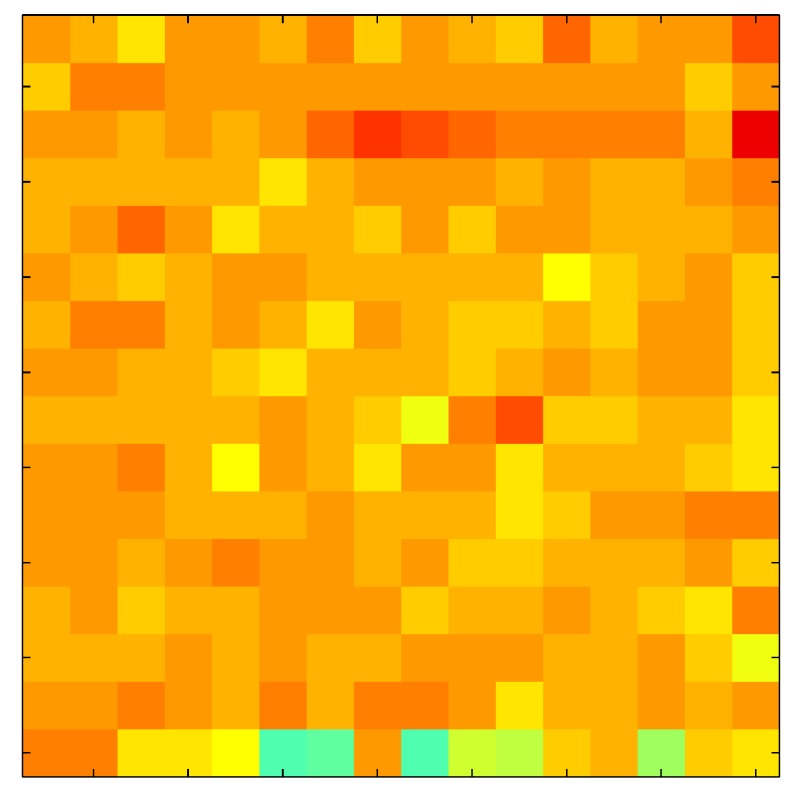	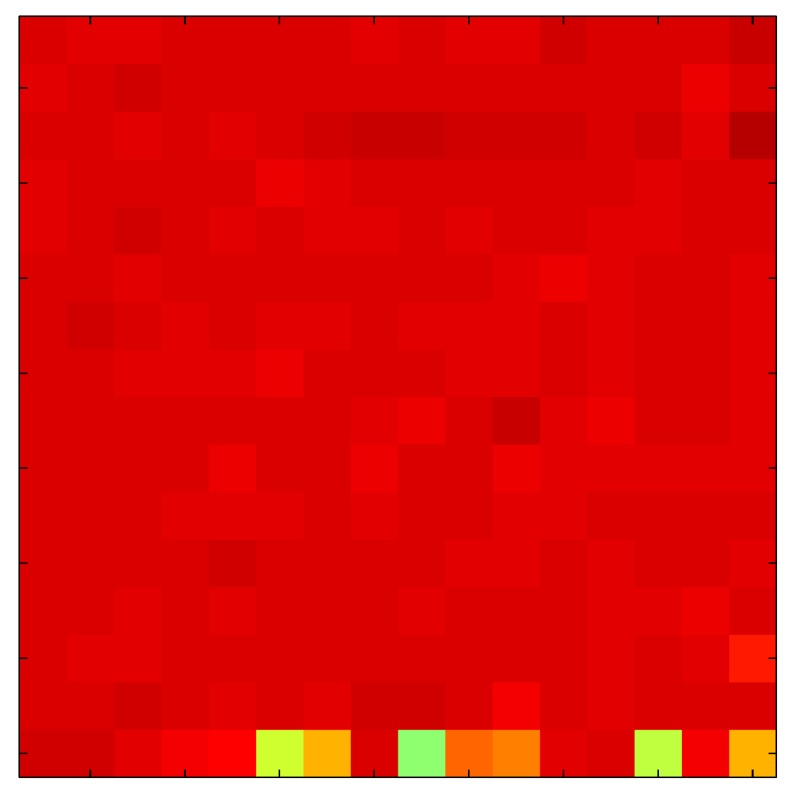	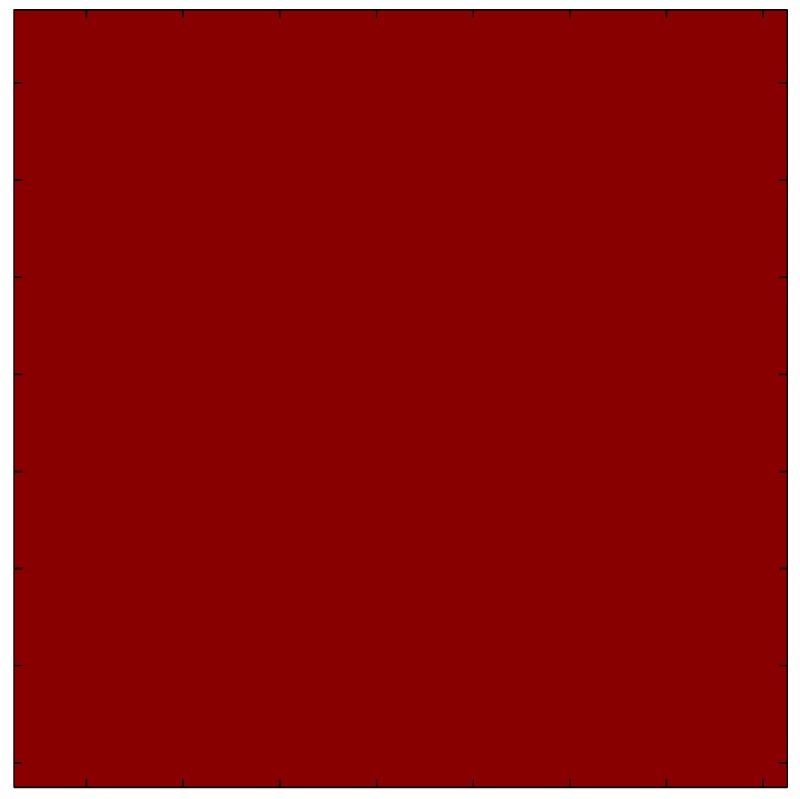
std(x)/FS:	0.02	0.03	0.04	0.03	0.03	0.04	
DOWN ➔	4.01 PSI	10.05 PSI	19.86 PSI	30.01 PSI	40.02 PSI	49.59 PSI	
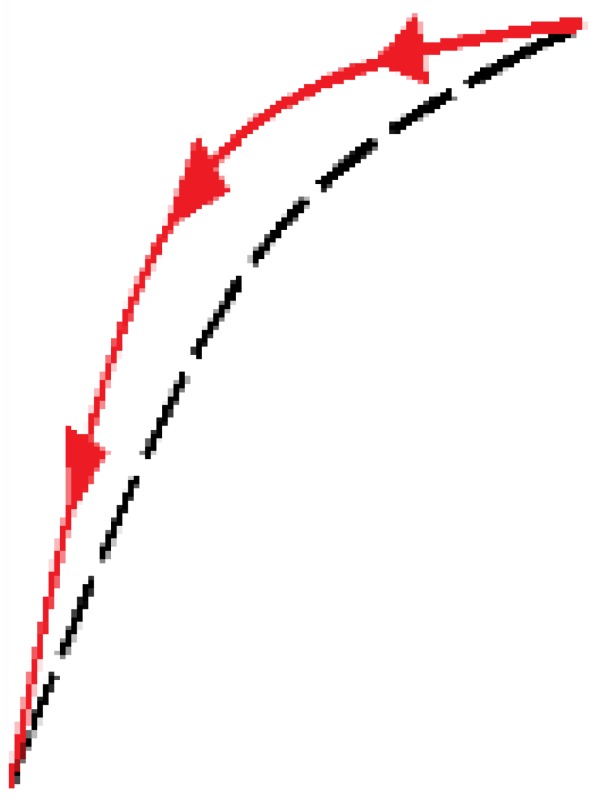	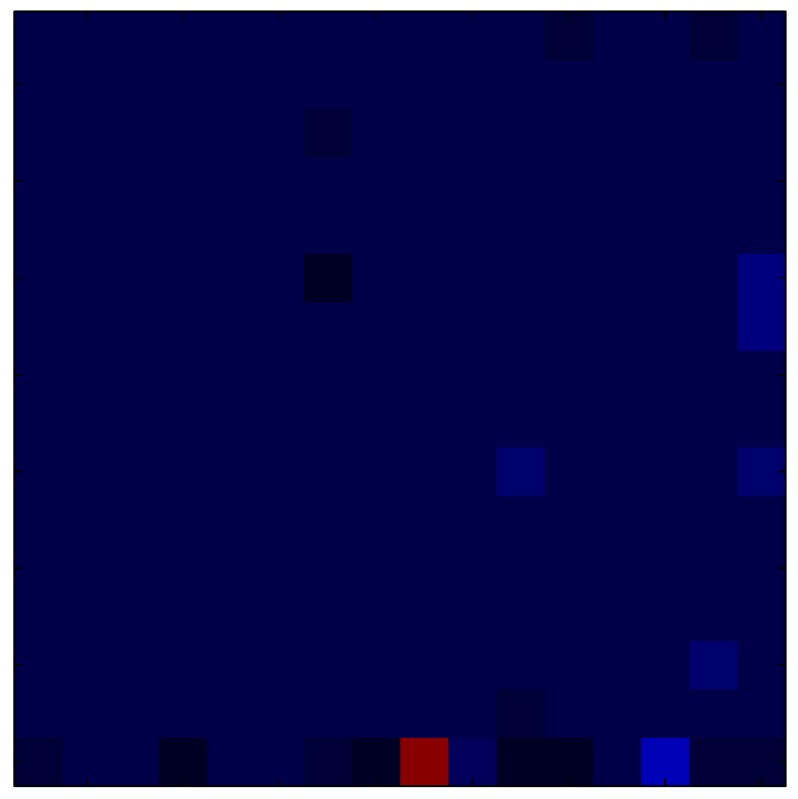	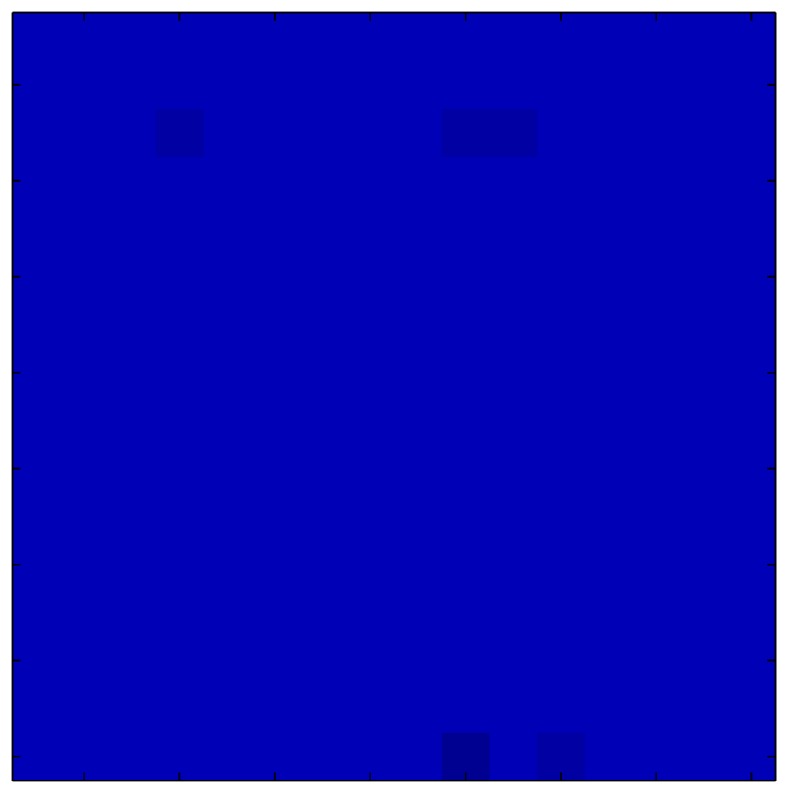	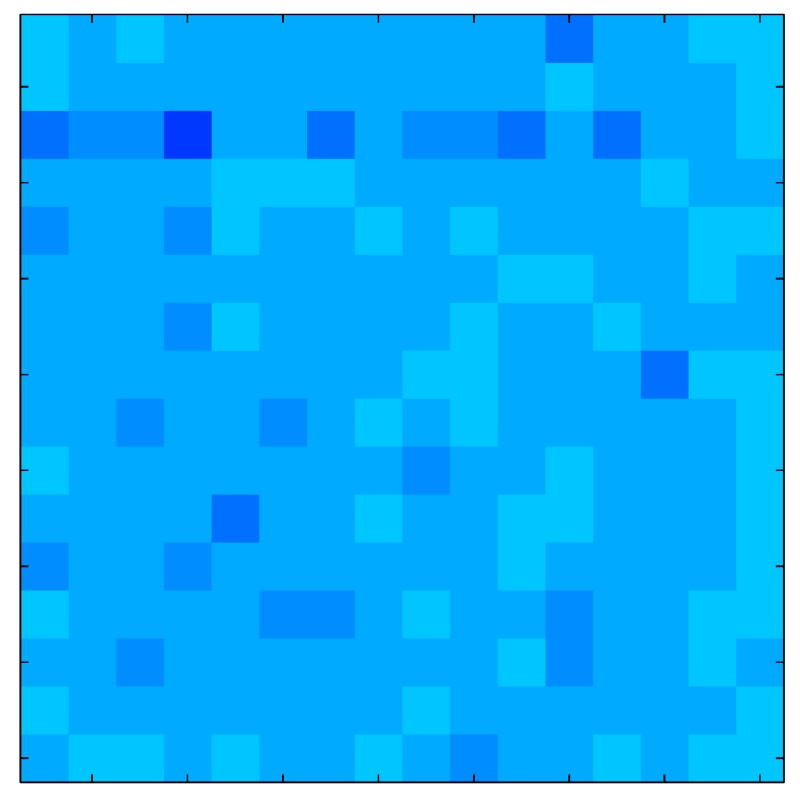	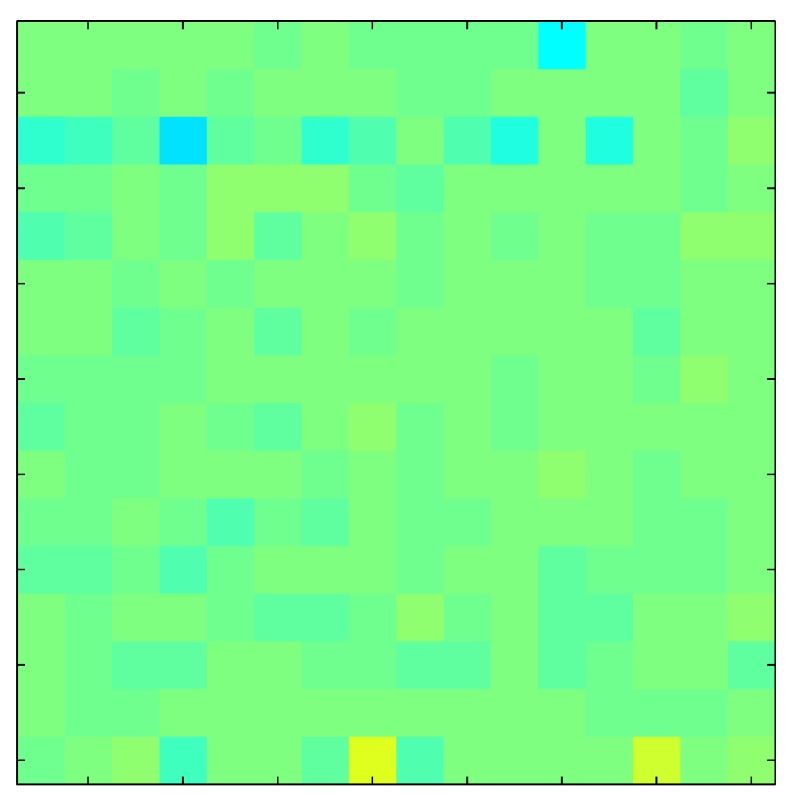	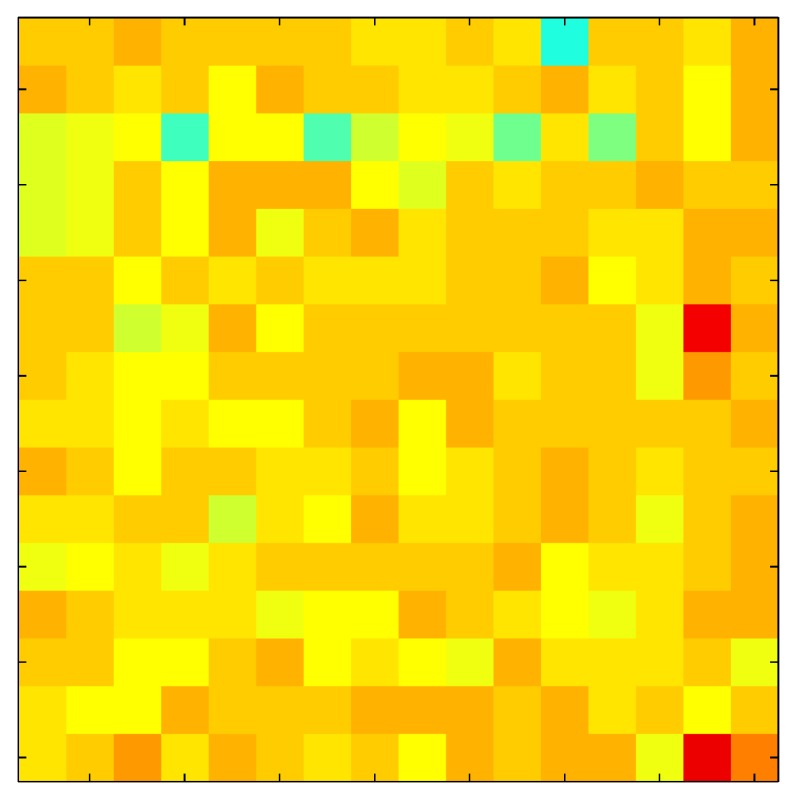	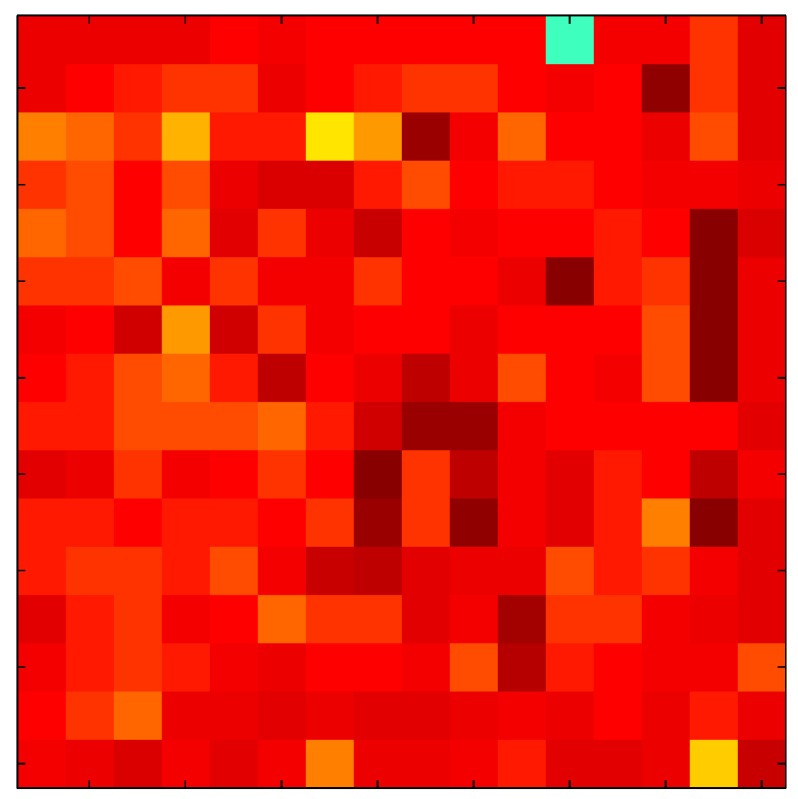	
std(x)/FS:	0.06	0	0.01	0.02	0.04	0.06	0
Absolute Distance	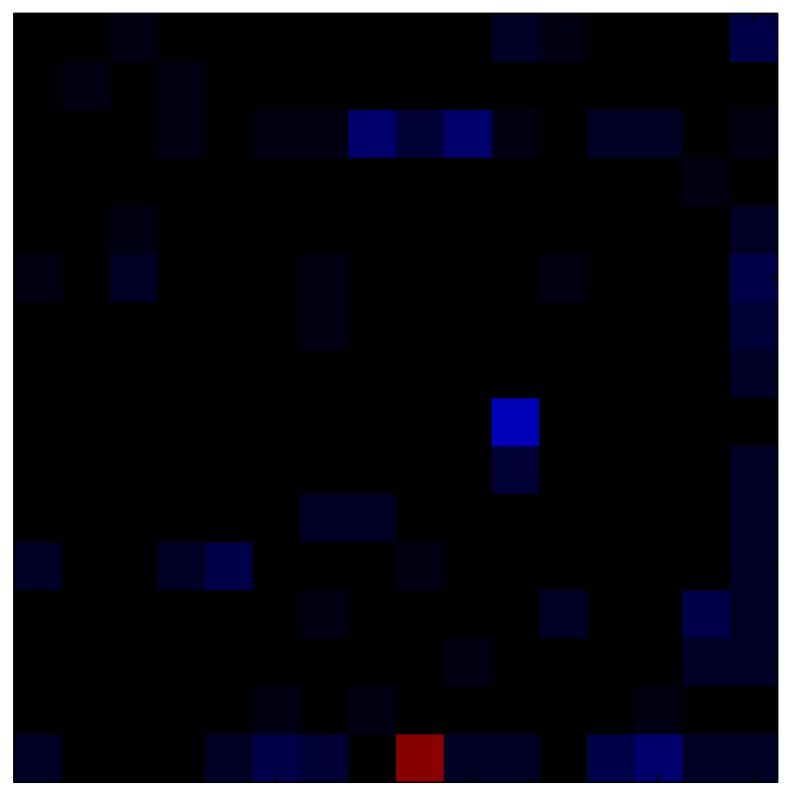	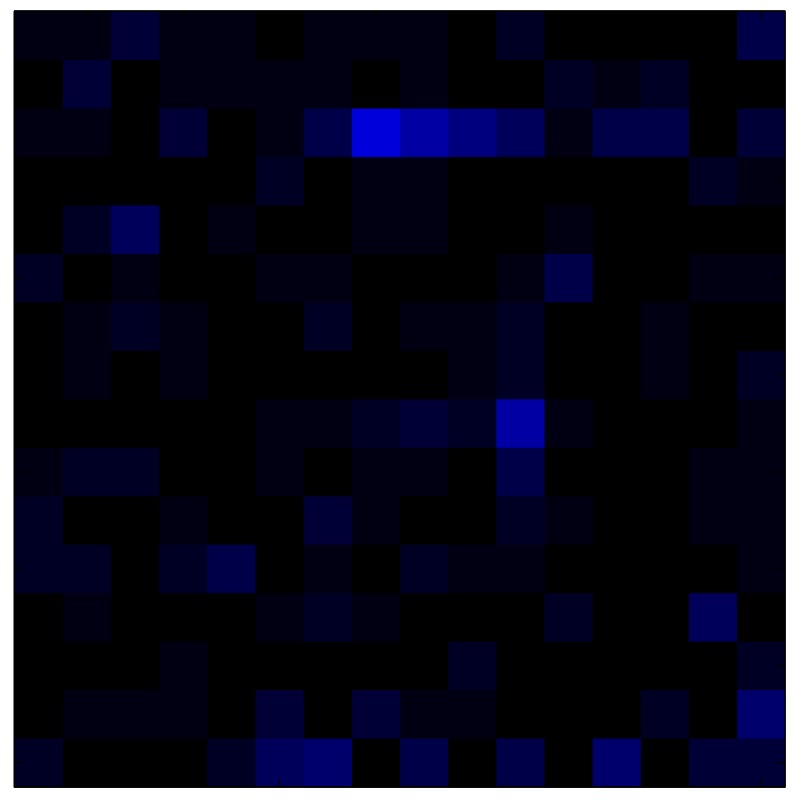	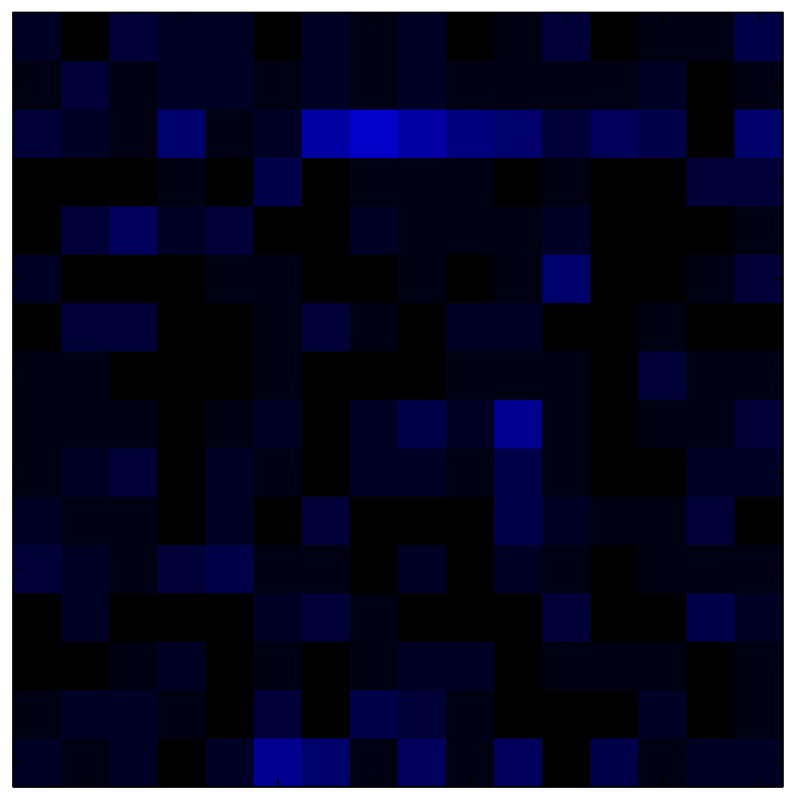	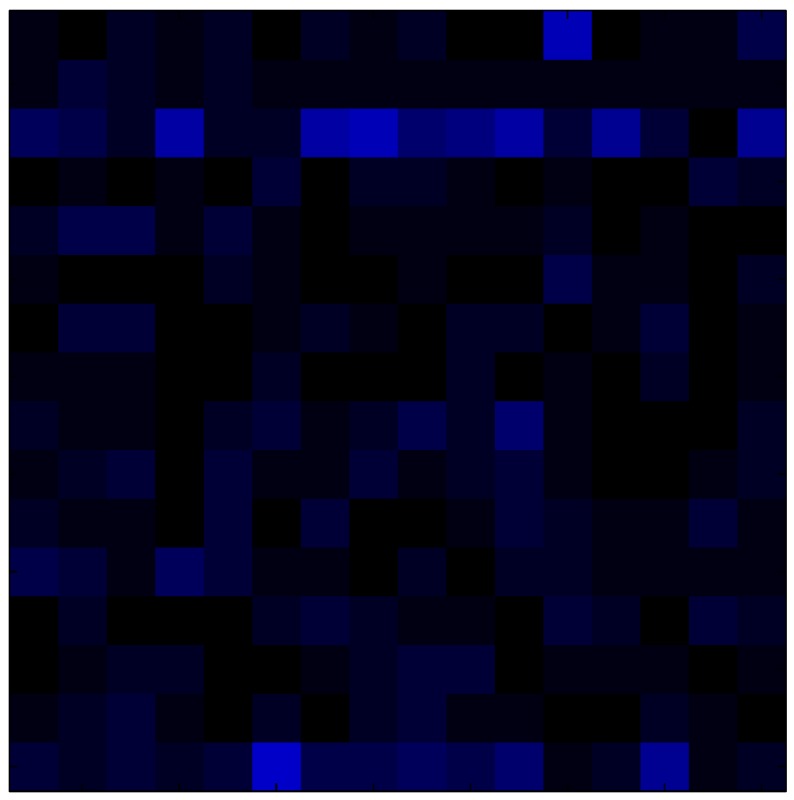	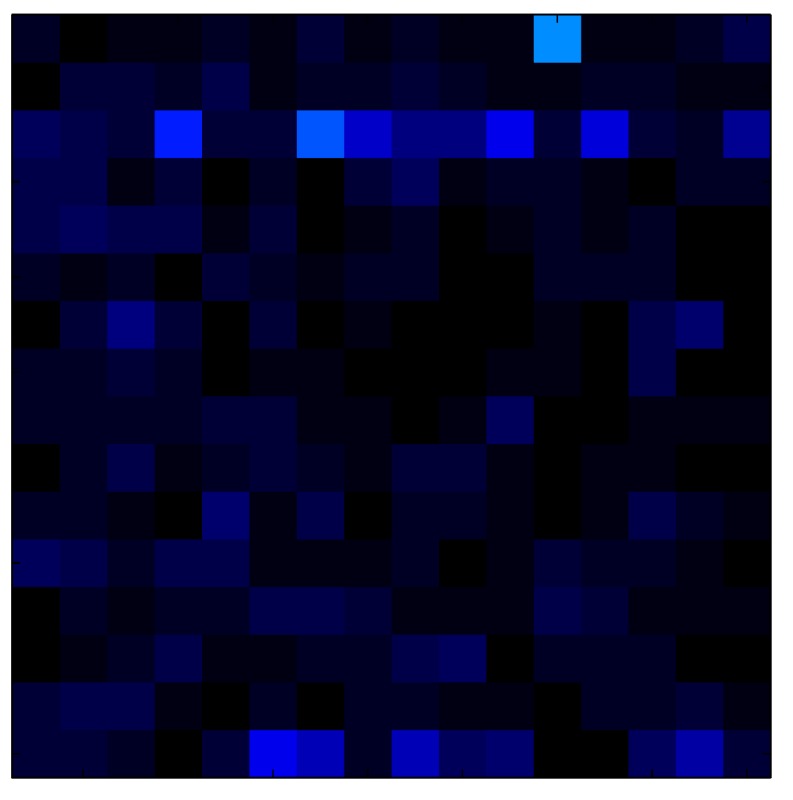	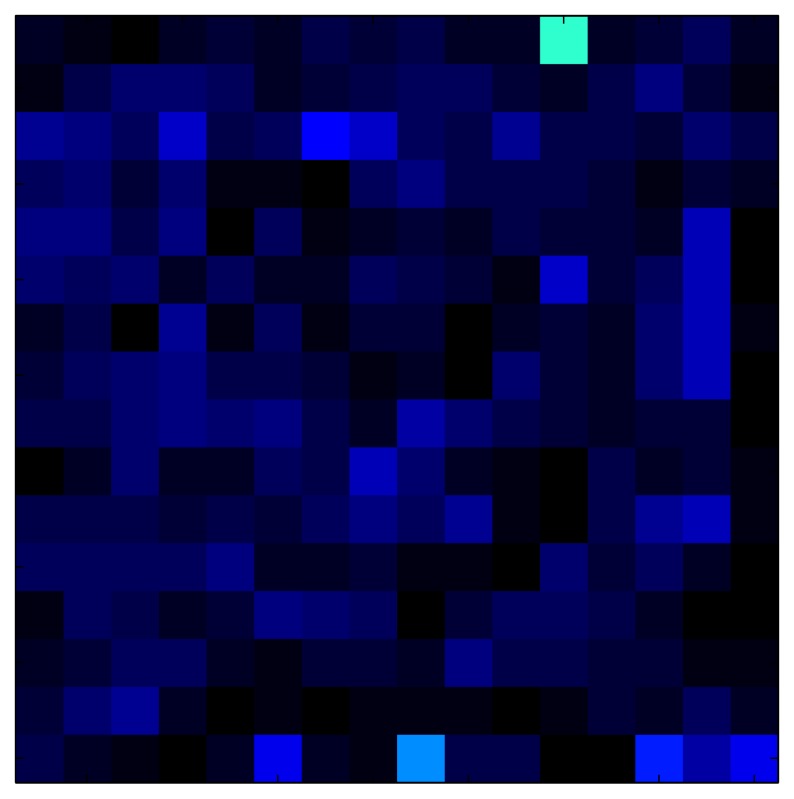	
:$\bar{x}/\mathrm{FS}$	0.02	0.02	0.03	0.03	0.04	0.07	

In addition, Table 8 and Table 9 show the sensor output when the force is exerted with a ring-shaped object. Table 8 shows the frames corresponding to the measured sensor output, while Table 9 shows the compensated frames with the ELAM method.

**Table 8:** Hysteresis loop frames measured before compensation with Ring object.

UP ➔	2.08 N	5.99 N	9.91 N	19.85 N	29.82 N
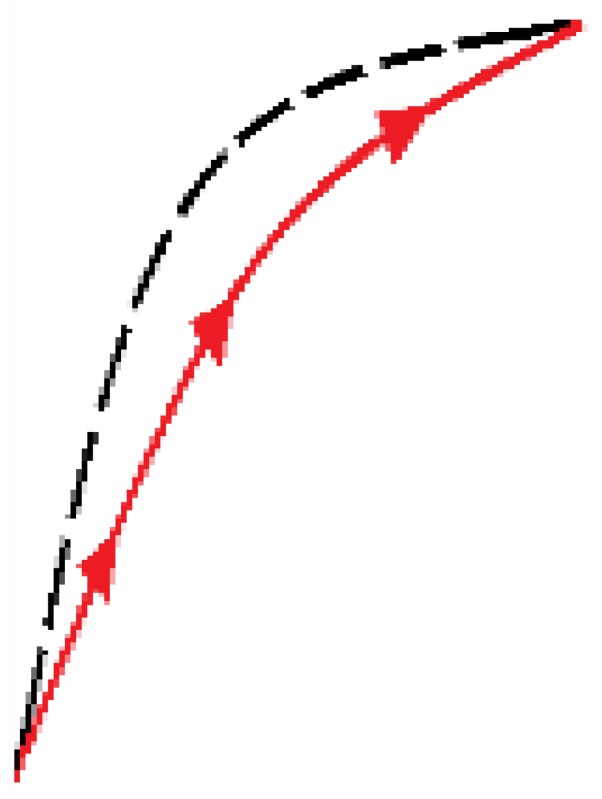	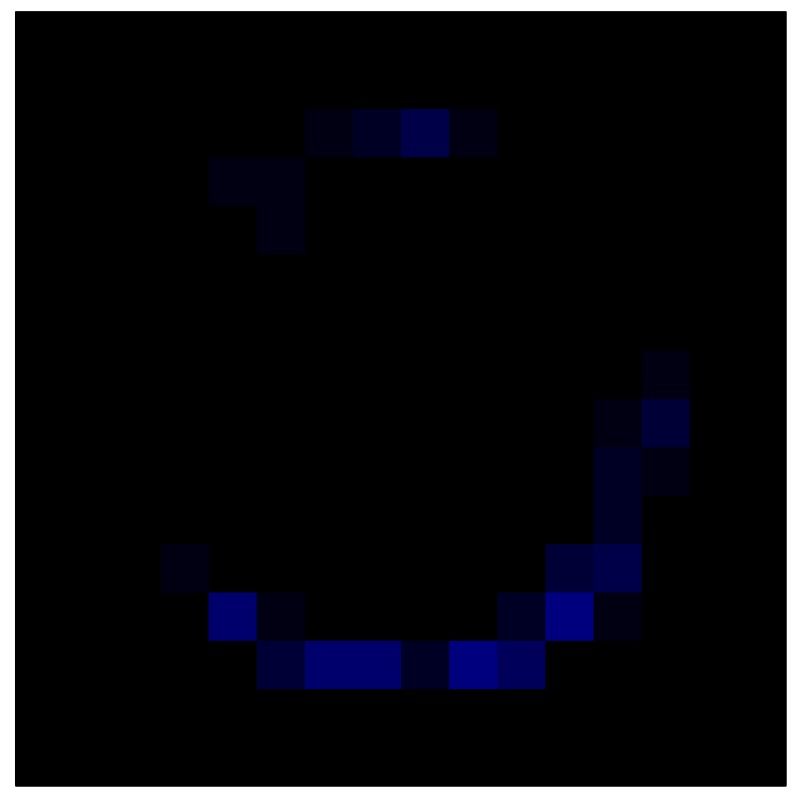	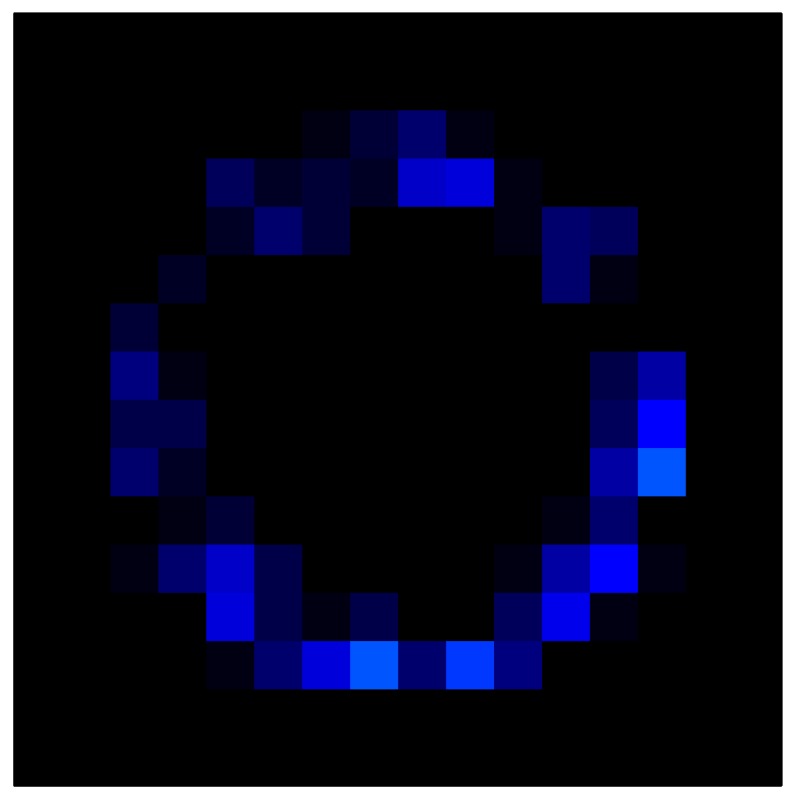	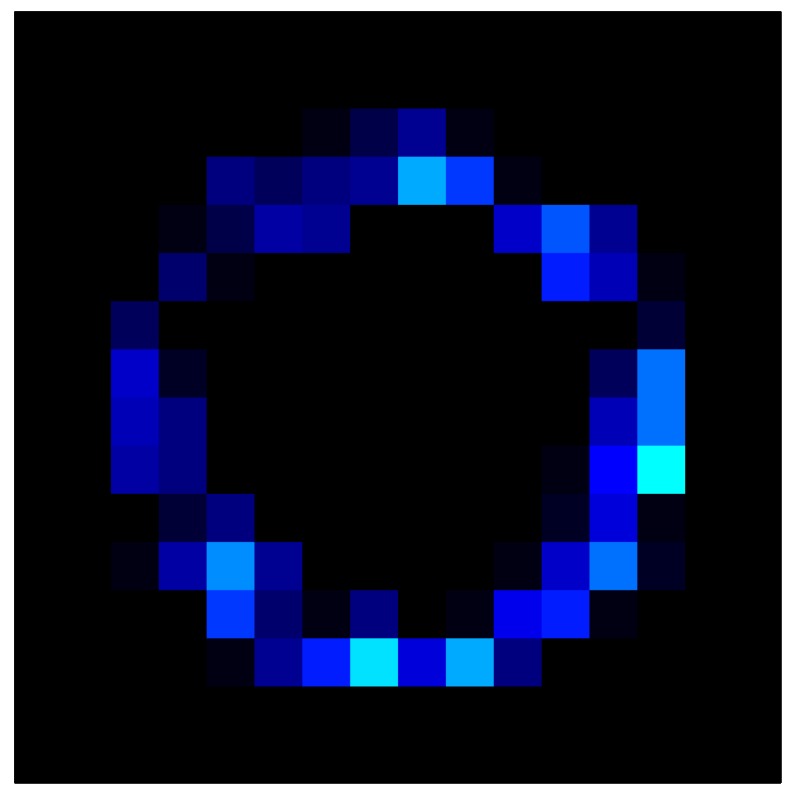	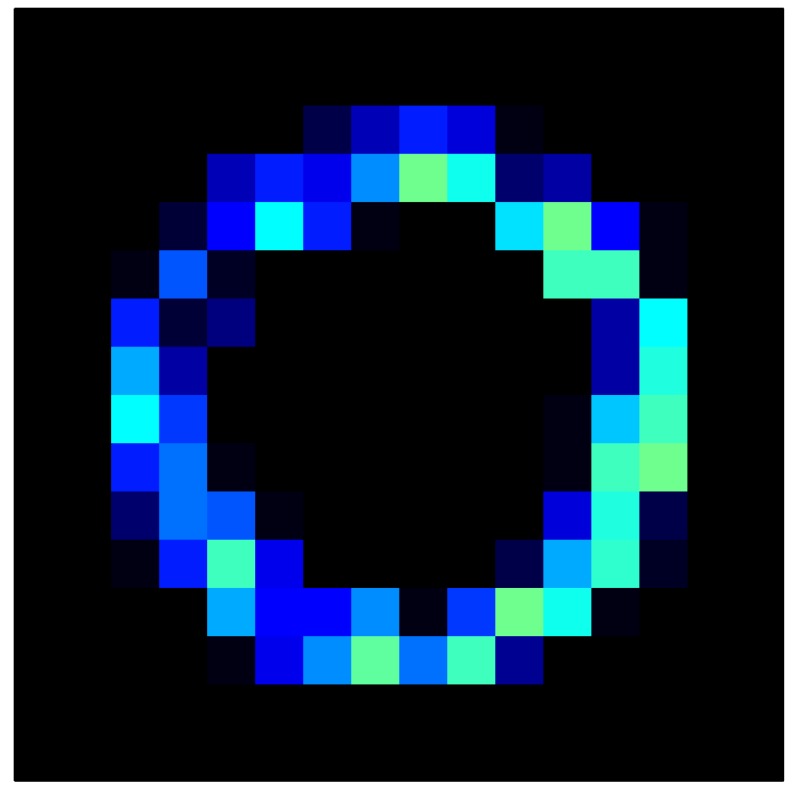	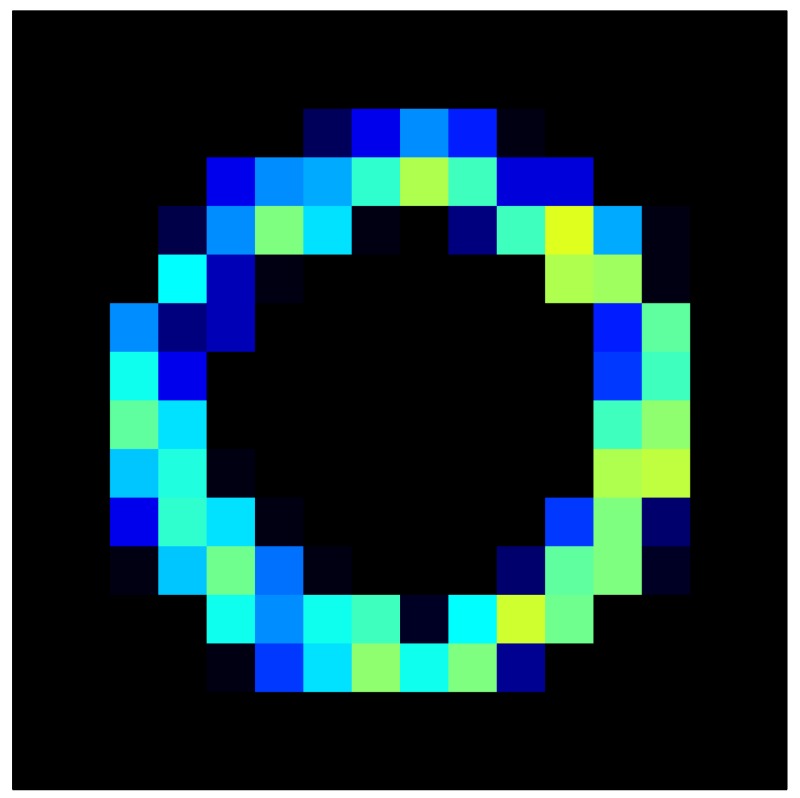
std(x)/FS:	0.05	0.11	0.16	0.25	0.31
DOWN ➔	2.03 N	6.00 N	10.04 N	20.04 N	30.09 N
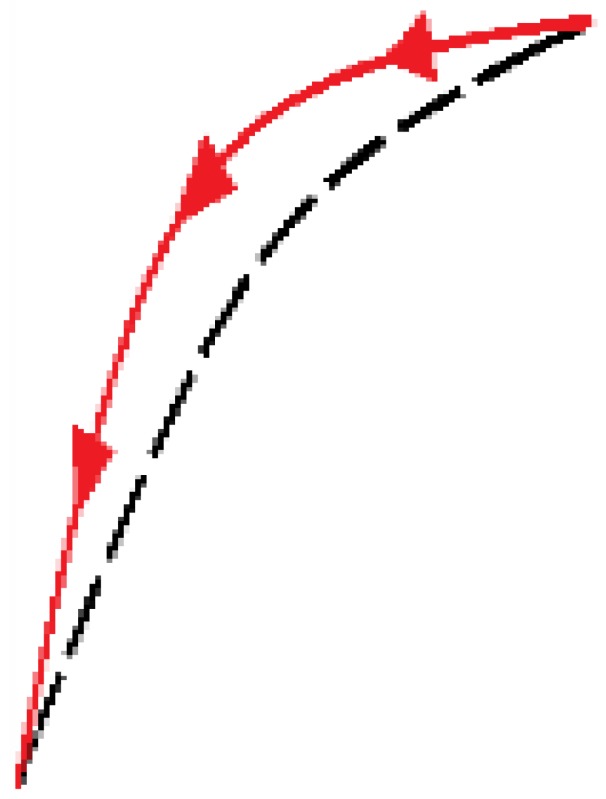	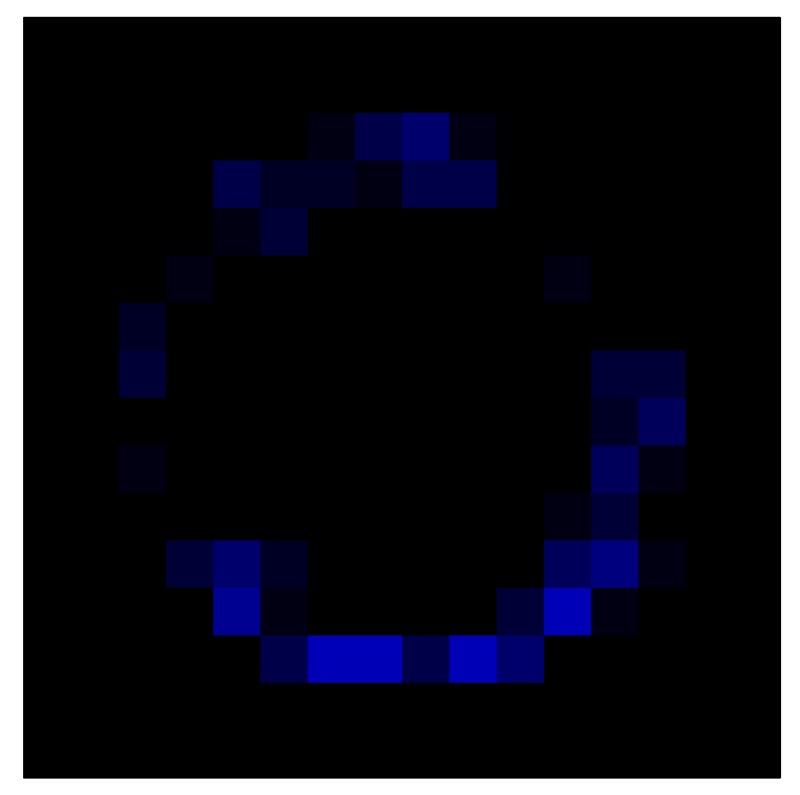	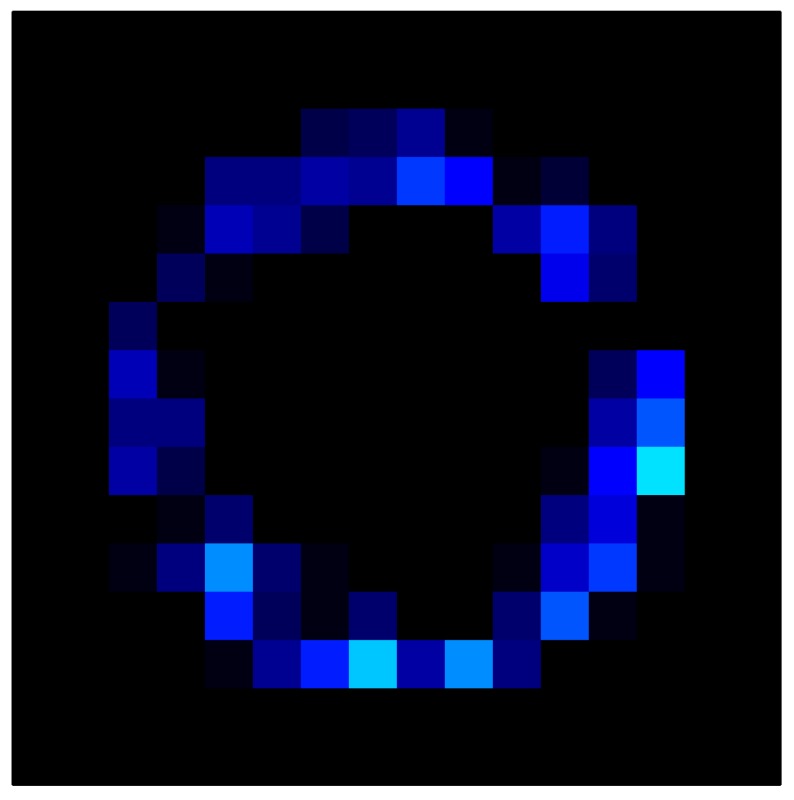	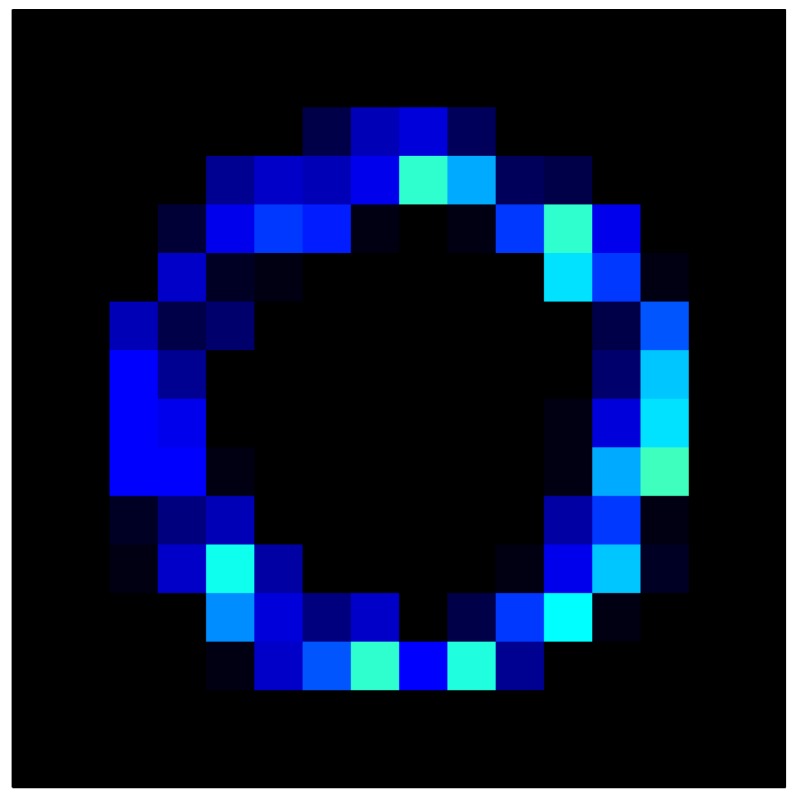	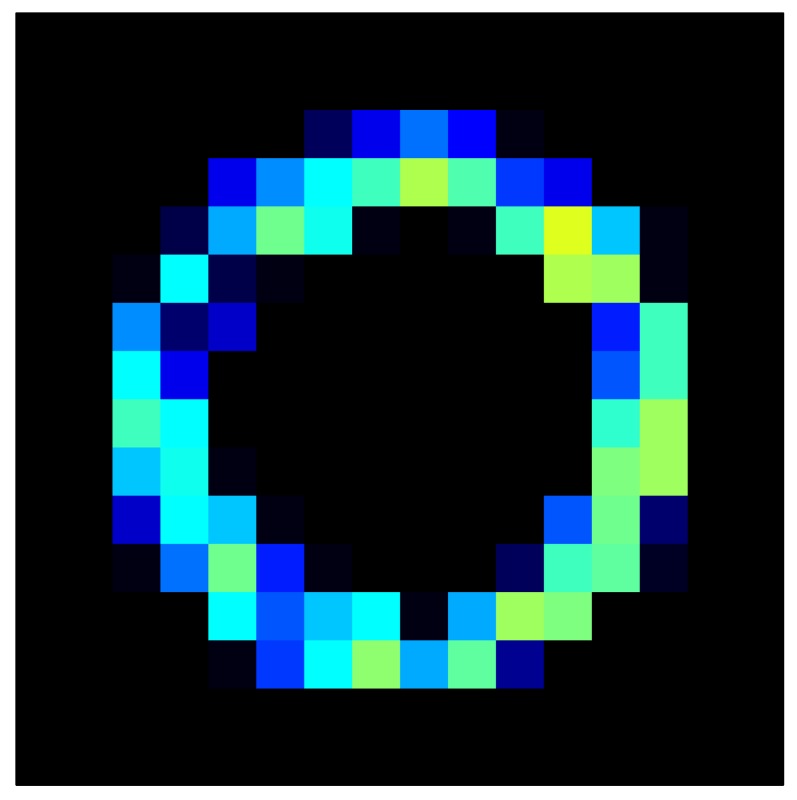	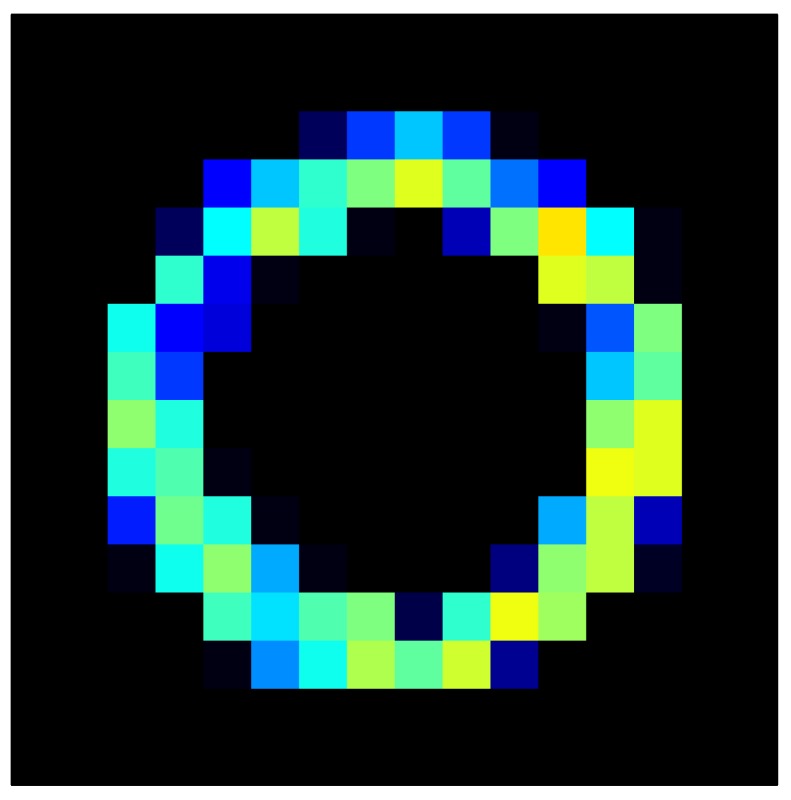
std(x)/FS:	0.07	0.14	0.20	0.30	0.34
Absolute Distance	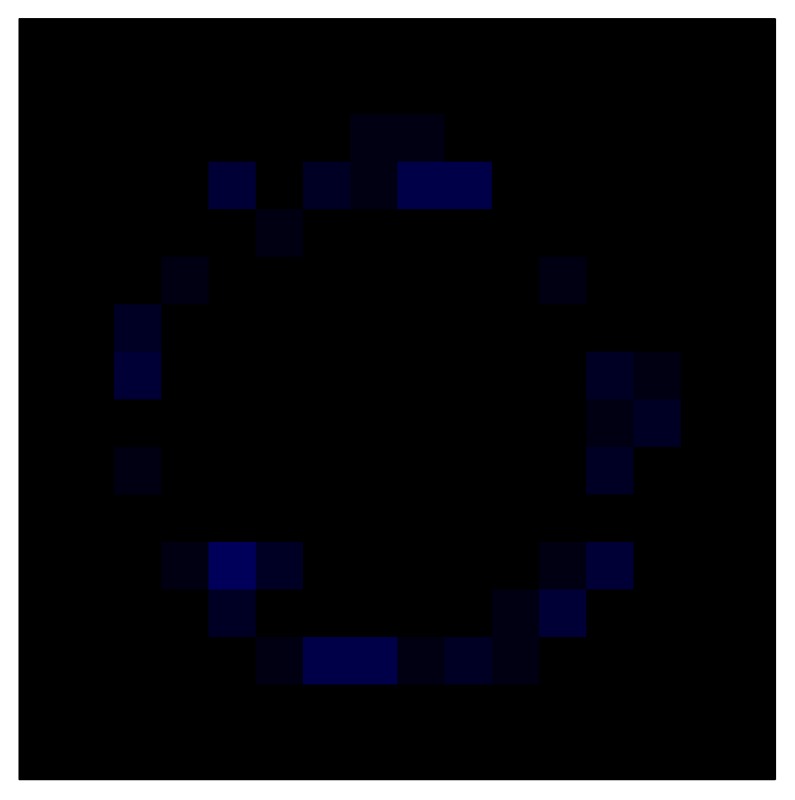	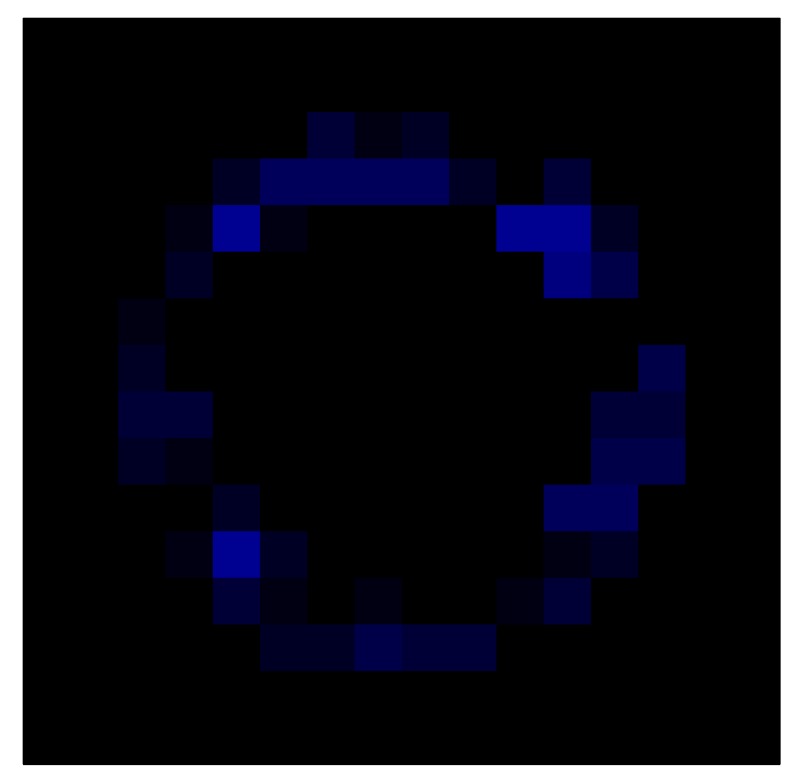	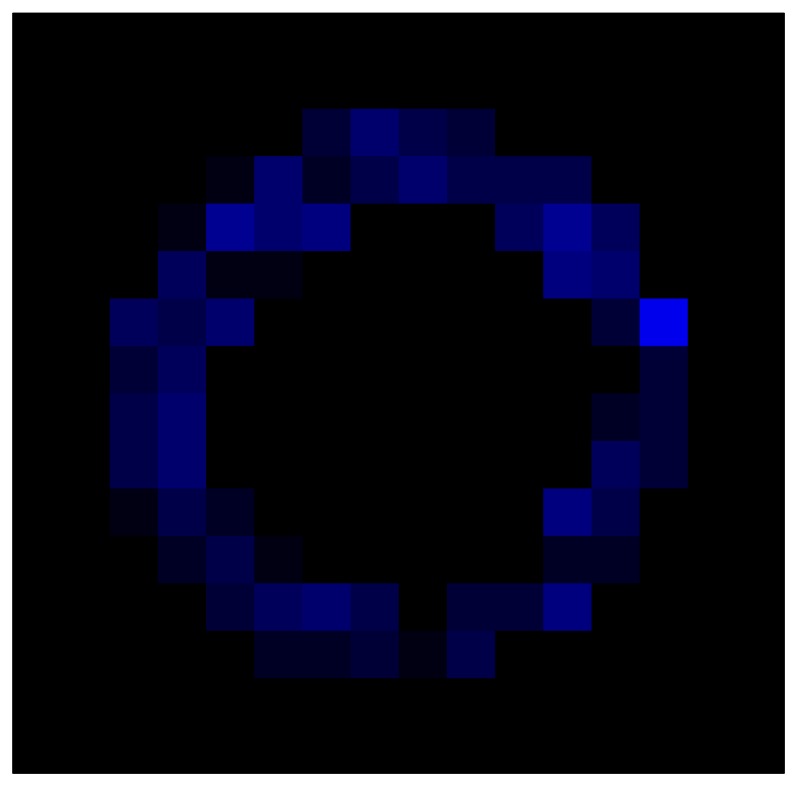	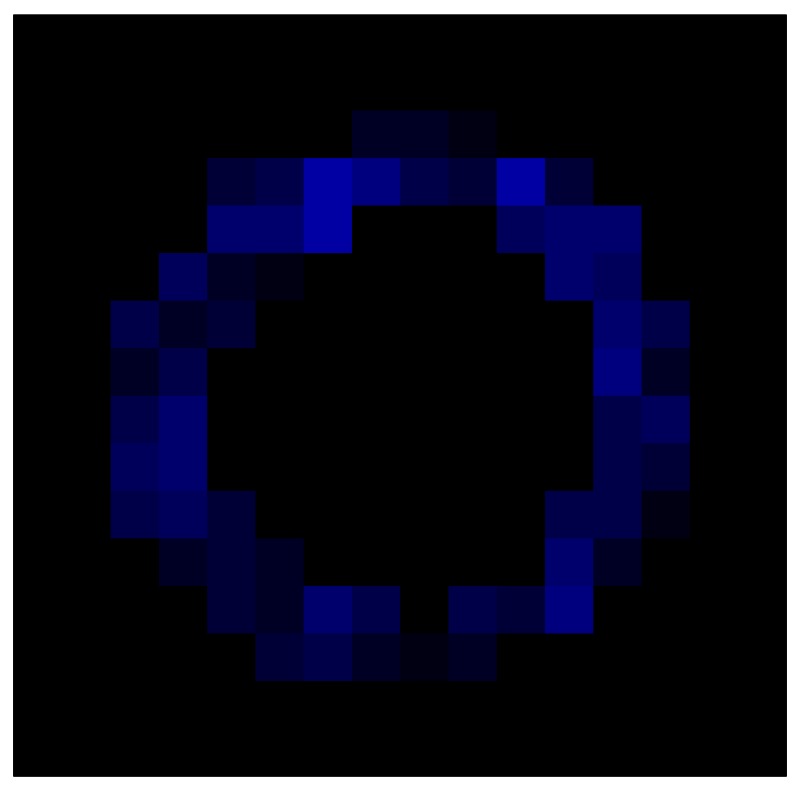	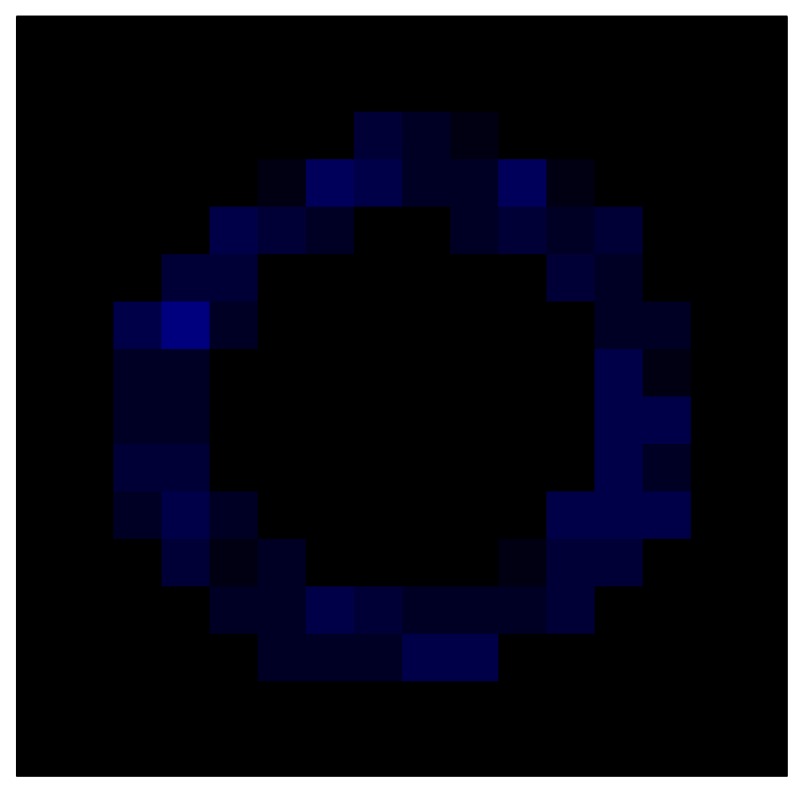
:$\bar{x}/\mathrm{FS}$	0.04	0.07	0.09	0.09	0.07

**Table 9:** Hysteresis loop frames with Ring object after compensation with ELAM method.

UP ➔	2.08 N	5.99 N	9.91 N	19.85 N	29.82 N
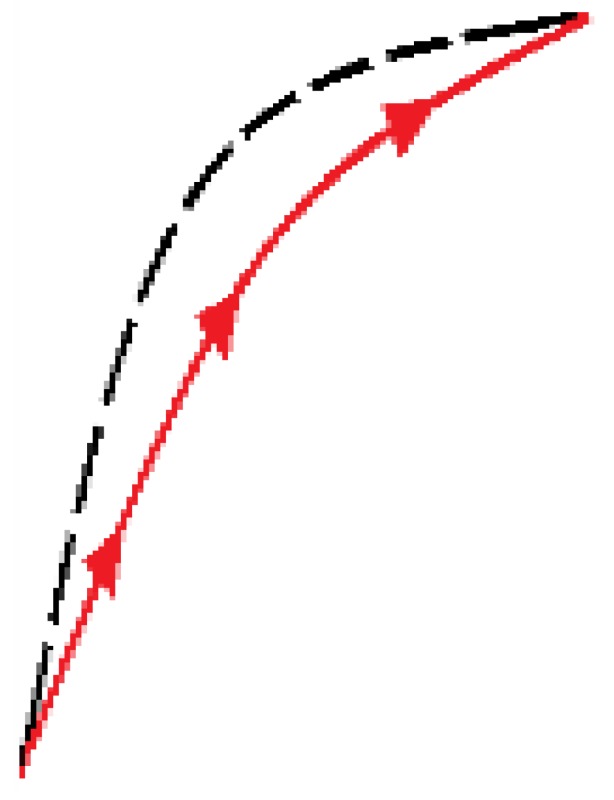	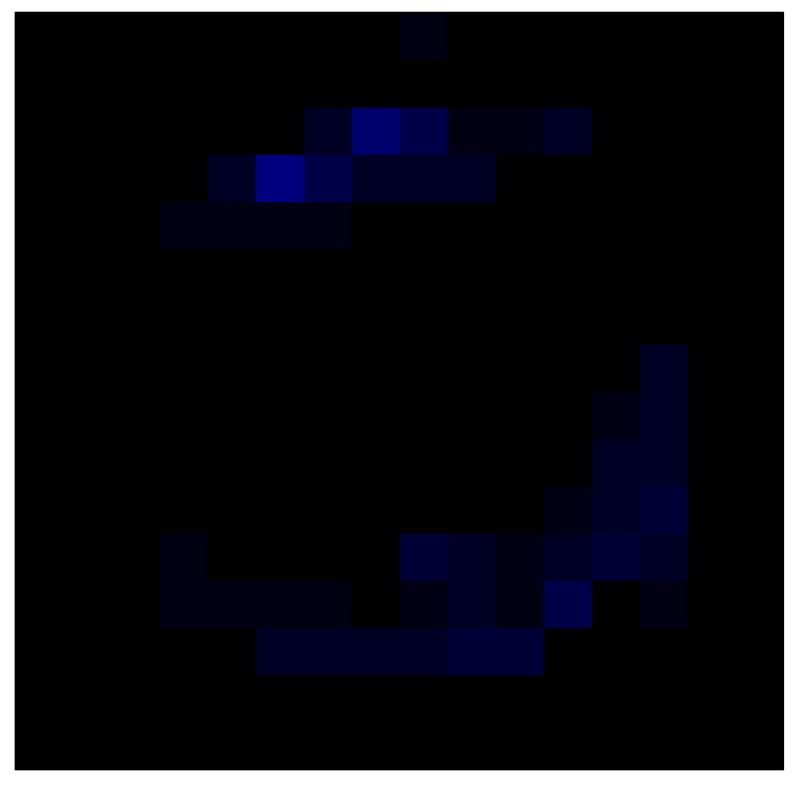	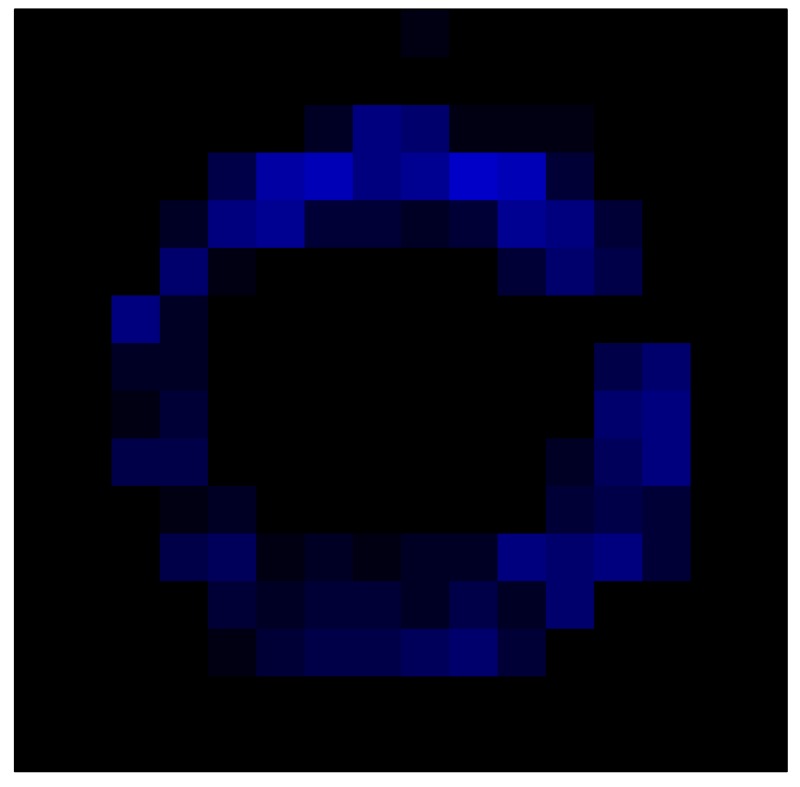	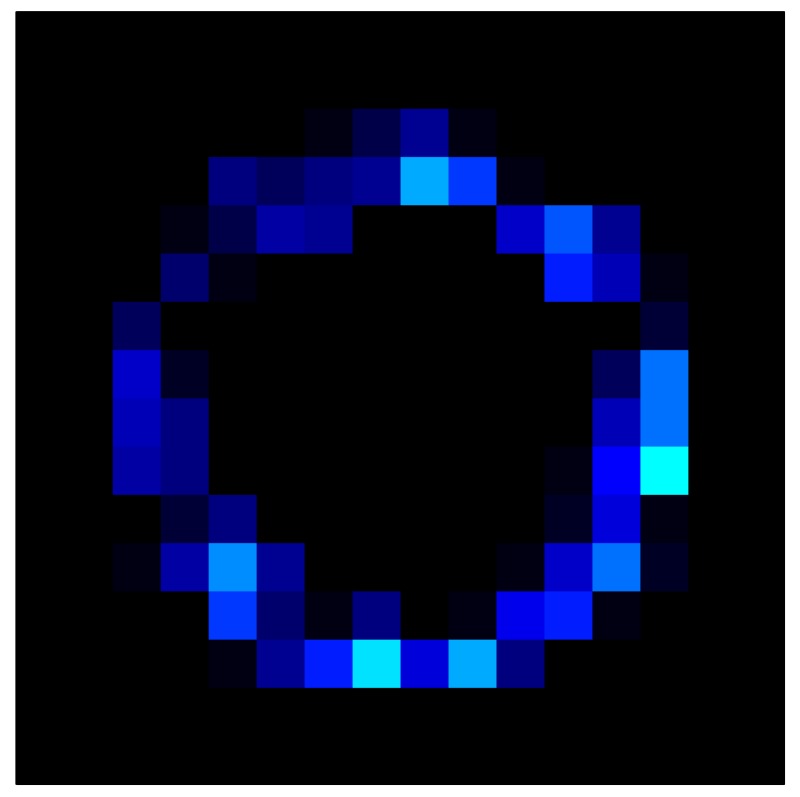	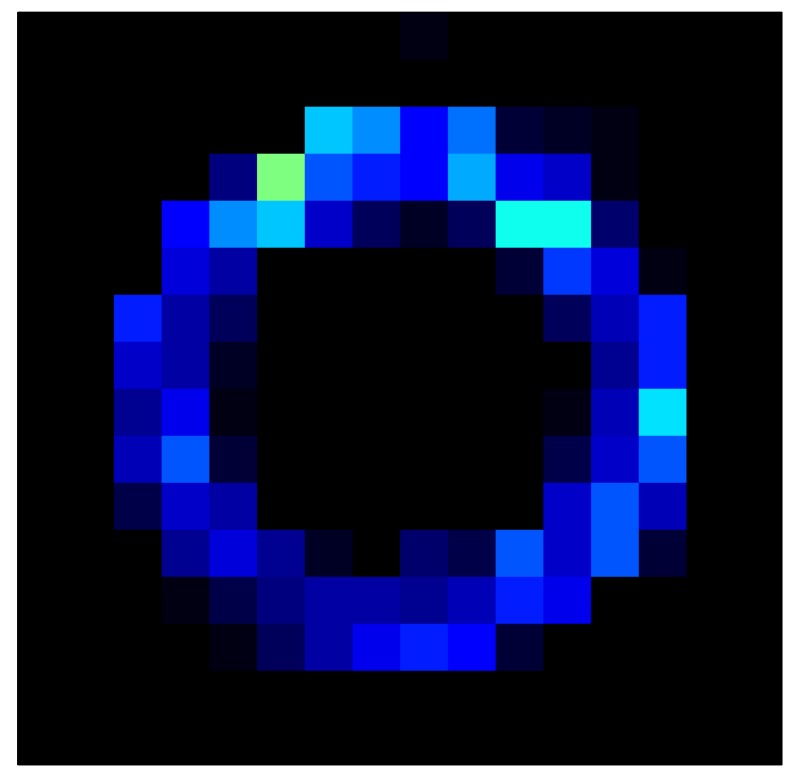	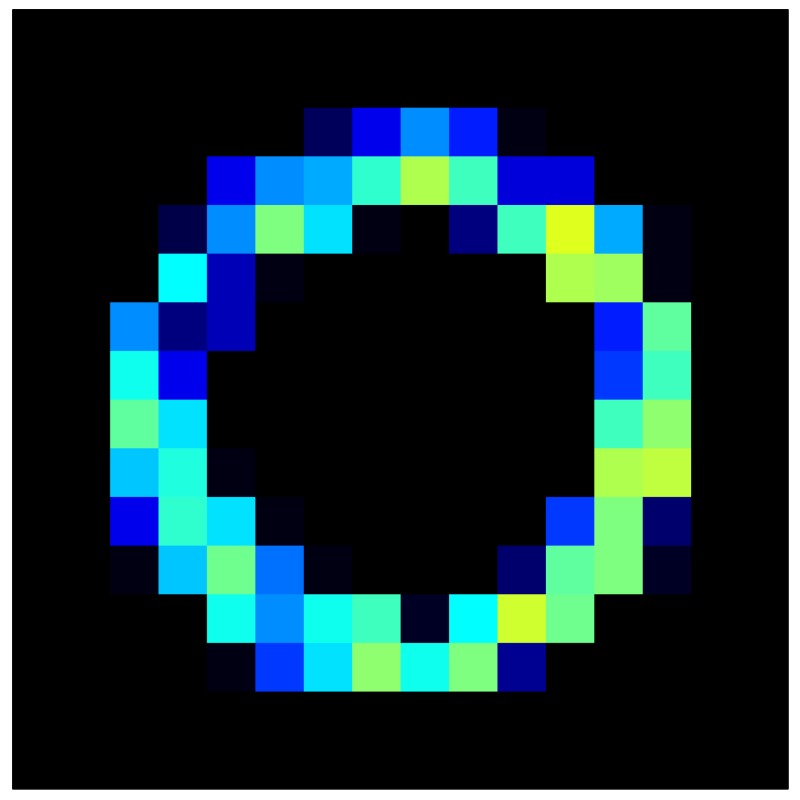
std(x)/FS:	0.03	0.06	0.09	0.16	0.22
DOWN ➔	2.03 N	6.00 N	10.04 N	20.04 N	30.09 N
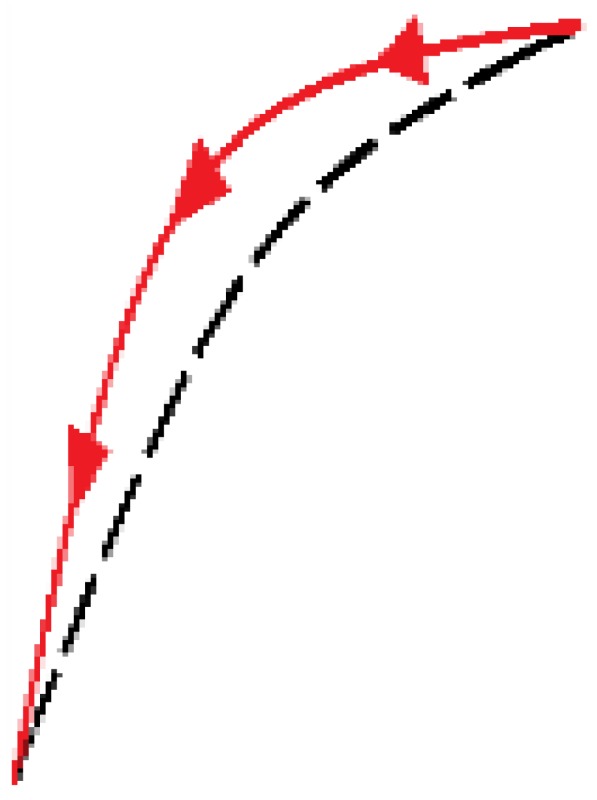	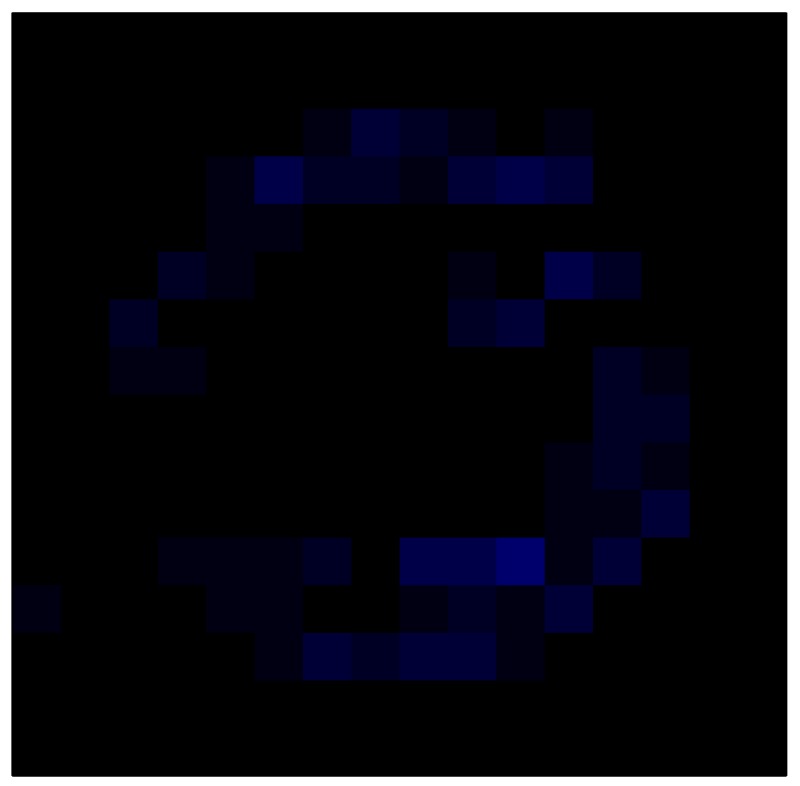	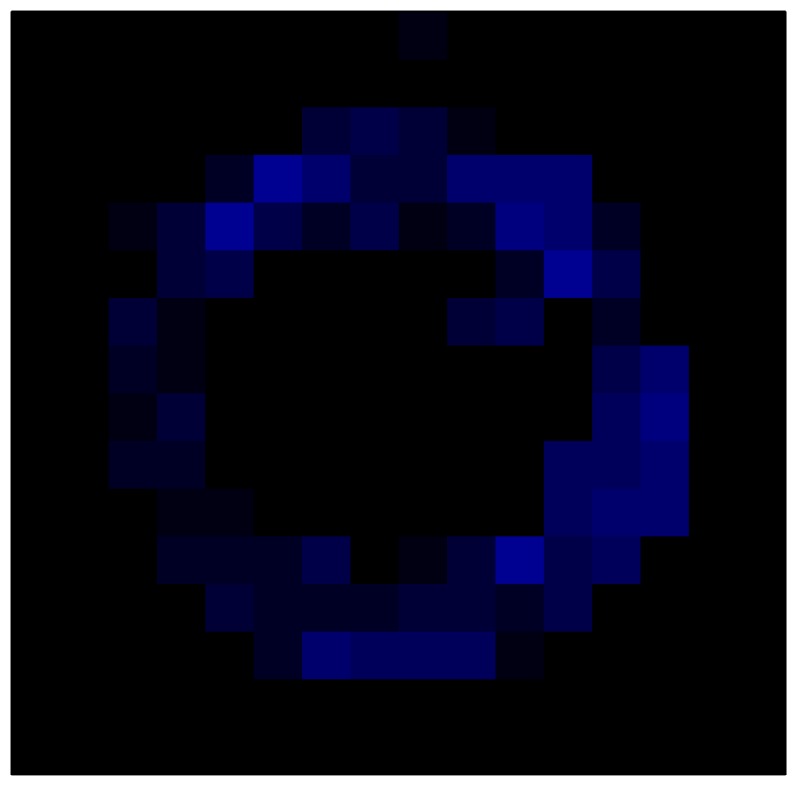	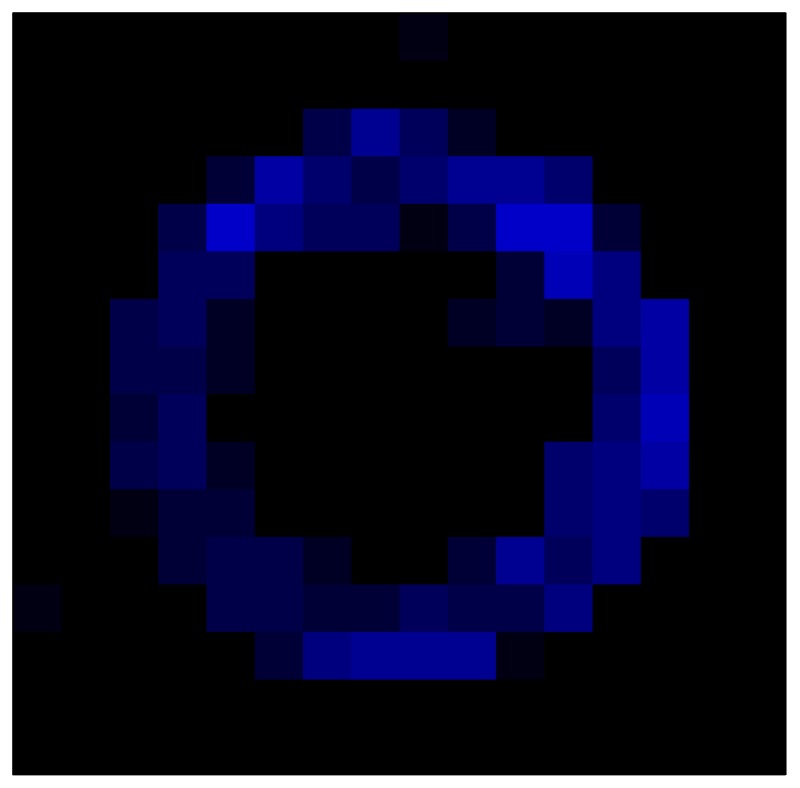	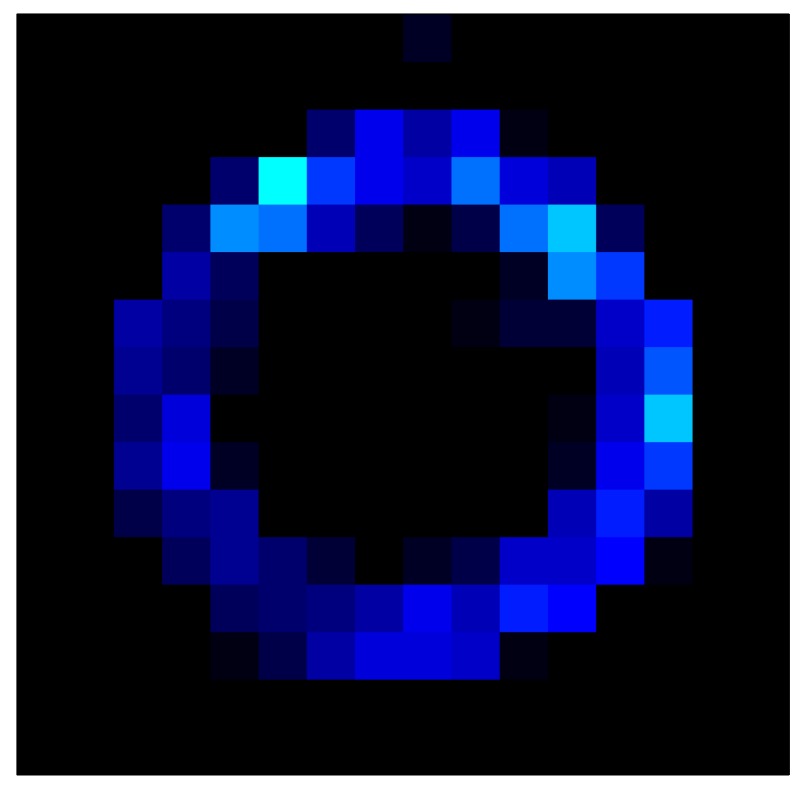	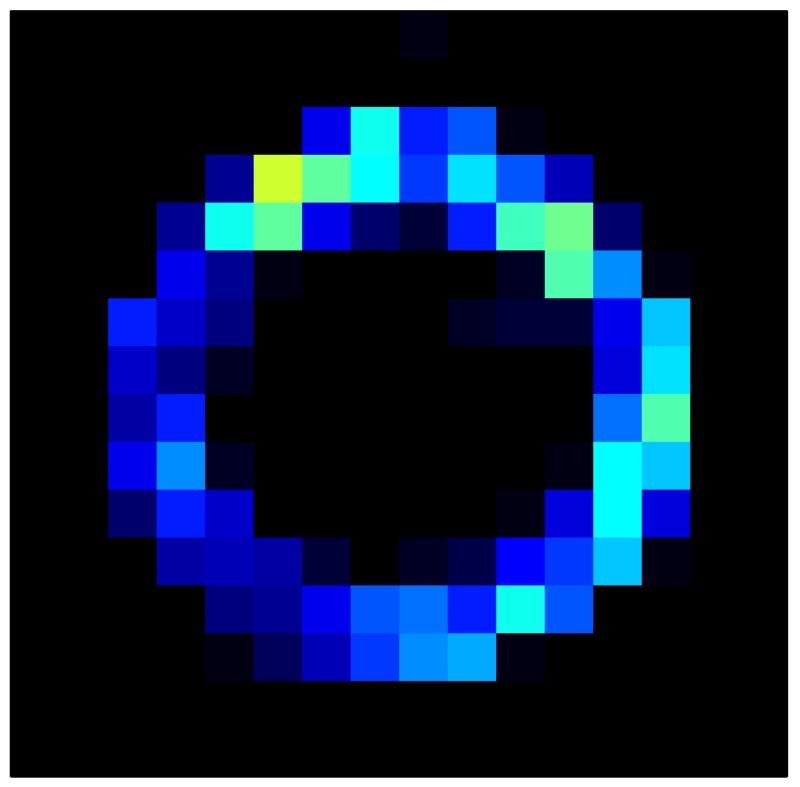
std(x)/FS:	0.03	0.05	0.07	0.14	0.21
Absolute Distance	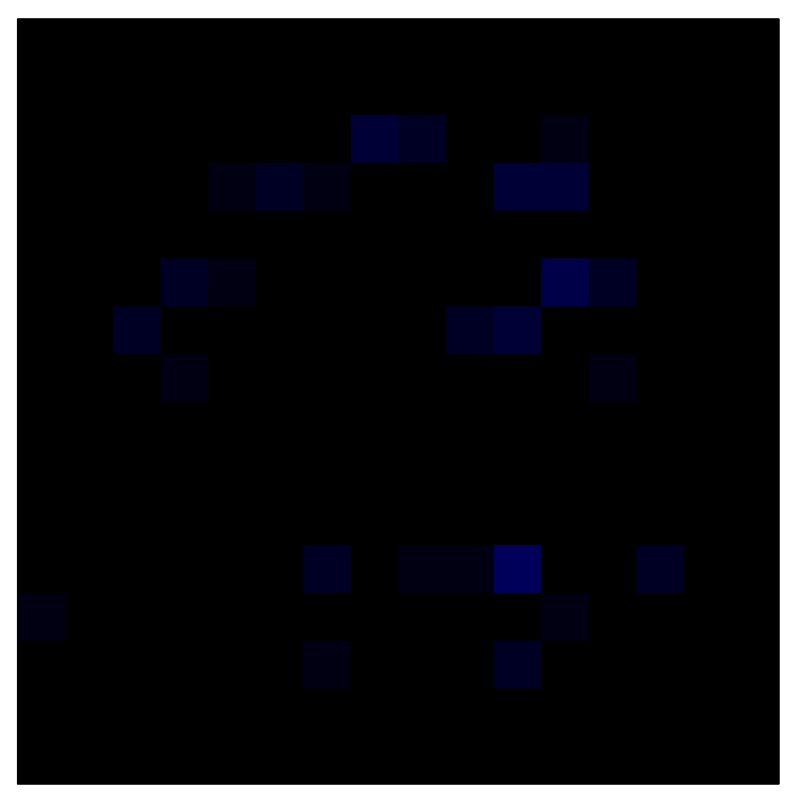	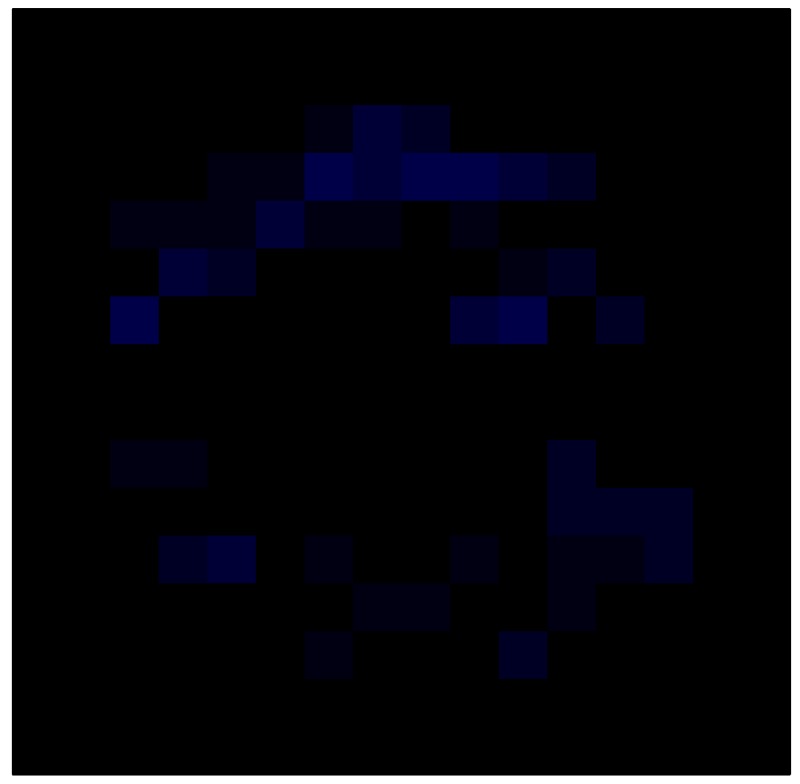	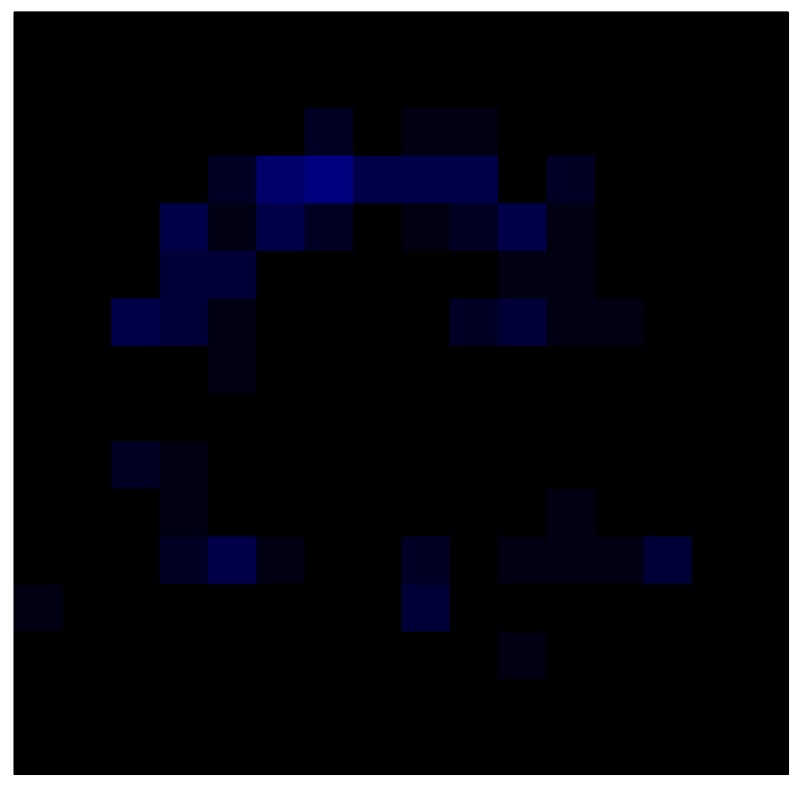	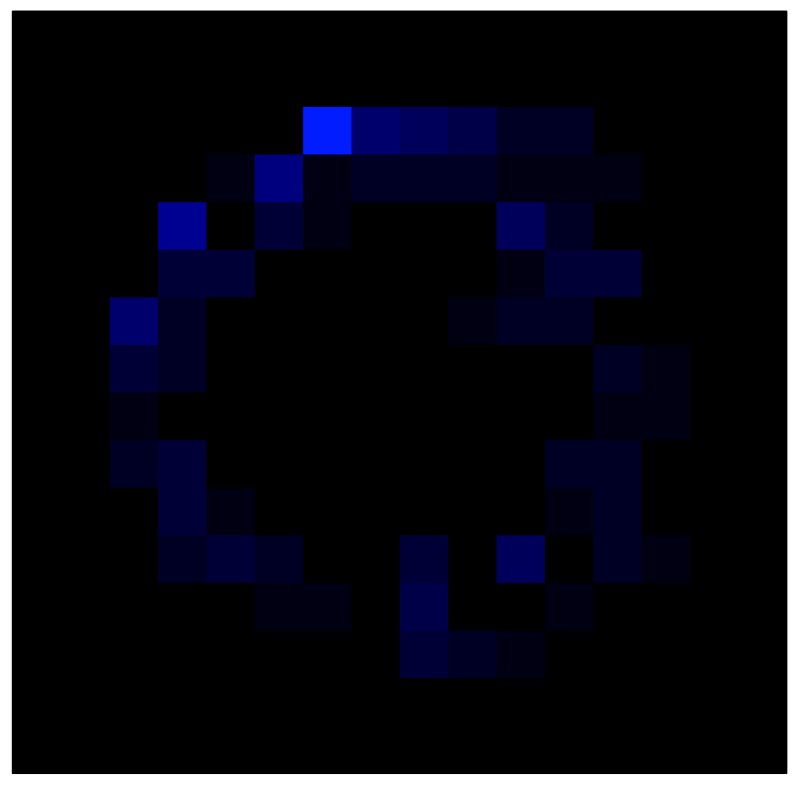	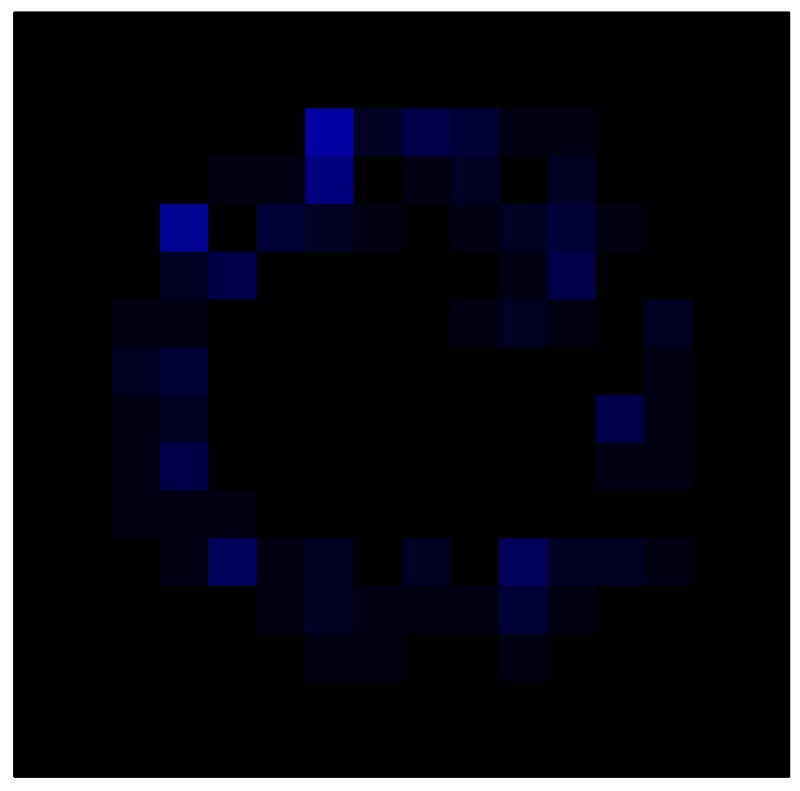
:$\bar{x}/\mathrm{FS}$	0.06	0.05	0.05	0.07	0.05

## 7. Conclusions

This paper presents a novel method to compensate for hysteresis nonlinearities observed in the response of a tactile sensor. The sensor shows a very pronounced hysteresis with high nonlinearity, and a large mismatching between the responses of different taxels. The proposed method builds a model that accurately fits the experimental data obtained in the characterization process. This so called ELAM method carries out a linear mapping of the external curves of the measured hysteresis loop to the curves of the inner cycles. Its main feature is the introduction of a split point in the curves to produce a different mapping to the left and right of this point, whose location is provided by an error minimization algorithm. The ELAM model is compared with the models obtained from three other approaches, the generalized Prandtl-Ishlinskii model (GPI), the modified Prandtl-Ishlisnkii model (MPI), and a model based on dominant curves built with polynomials (POLY). The results show that the ELAM model fits the measured data more accurately than the other three methods, especially in the ascending curves, where the other methods perform worse. Another very remarkable advantage of the ELAM method *versus* the other three methods is that the involved mathematical operations are simpler, so they can be implemented more easily in FPGA devices in order to cope with real-time applications. For instance, the ELAM method does uses neither play operators nor exponential functions that may require the use of additional computational resources. Moreover, the number of parameters to be identified by the error minimization algorithm is higher in the other methods, and they also require a prior selection of the appropriate functions to build the model. The performance of the proposed method is shown with data obtained from measurements of the sensor output when a uniform pressure is exerted on the entire matrix, and also when the force is exerted by objects with different shapes. The output of each taxel is compensated with its own ELAM hysteresis model and a significant reduction of the hysteresis, nonlinearity and mismatching errors, is observed. The ELAM method fits very well to complex and asymmetrical hysteresis curves, which cannot be characterized by mathematical functions in a direct way. This allows the application of the strategy followed by the ELAM method to other types of sensors or actuators. Moreover, it is a flexible method, since more split points can be added to divide the curves into a larger number of segments, so different mappings can be done for each segment and a good fitting can be achieved despite the complexity of the hysteresis loops.
